# Benzyl methyl morpholinium hydroxide (BMMorph)OH: a new basic ionic liquid for *N*-alkylation of bioactive N-heterocycles

**DOI:** 10.1039/d5ra10042a

**Published:** 2026-02-09

**Authors:** Mohammad Navid Soltani Rad, Somayeh Behrouz, Hamid Reza Mohammadnia Afroozi

**Affiliations:** a Department of Chemistry, Shiraz University of Technology Shiraz 71555-313 Iran soltani@sutech.ac.ir behrouz@sutech.ac.ir +98 71 3735 4520 +98 71 3735 4500; b Medicinal Chemistry Research Laboratory, Novel Technology for Health Research Center, Shiraz University of Technology Shiraz 71555-313 Iran

## Abstract

We report benzyl methyl morpholinium hydroxide ([BMMorph]OH) as a novel, stable basic ionic liquid that exhibits dual functionality as both an efficient reaction medium and a strong base for the *N*-alkylation of bioactive N-heterocycles. [BMMorph]OH was synthesized through a simple two-step process: quaternization of 4-methylmorpholine with benzyl bromide, followed by anion exchange with potassium hydroxide. Comprehensive characterization by TGA, IR, and NMR confirmed good thermal stability (up to ∼150 °C) and broad solubility in common polar solvents. Under optimized neat conditions (100 °C, 0.4 mol% [BMMorph]OH), diverse heterocycles—including purines (adenine, theophylline), pyrimidines (uracil, thymine), and azoles (imidazoles, benzimidazoles)—were efficiently alkylated with carbon electrophiles such as alkyl halides, epoxides, and Michael acceptors, affording good to excellent isolated yields. Comparative studies demonstrated that [BMMorph]OH outperformed conventional hydroxide ionic liquids (*e.g.*, imidazolium and quaternary ammonium hydroxides). Notably, adenine alkylation showed exceptional regioselectivity, favoring the N9 isomer over N7, attributed to hydrogen-bonding interactions with the morpholinium oxygen. The ionic liquid was recyclable for at least five cycles, with only gradual activity loss due to atmospheric CO_2_ absorption. Beyond its catalytic efficiency, [BMMorph]OH offers a sustainable alternative for nucleobase alkylation in carboacyclic nucleoside synthesis, reducing reliance on toxic solvents such as DMF and DMSO. These findings establish [BMMorph]OH as a promising, reusable basic ionic liquid for green C–N bond formation in heterocyclic chemistry.

## Abstract 2

We report benzyl methyl morpholinium hydroxide ([BMMorph]OH) as a novel, stable basic ionic liquid that exhibits dual functionality as both an efficient reaction medium and a strong base for the *N*-alkylation of bioactive N-heterocycles.

## Graphical abstract

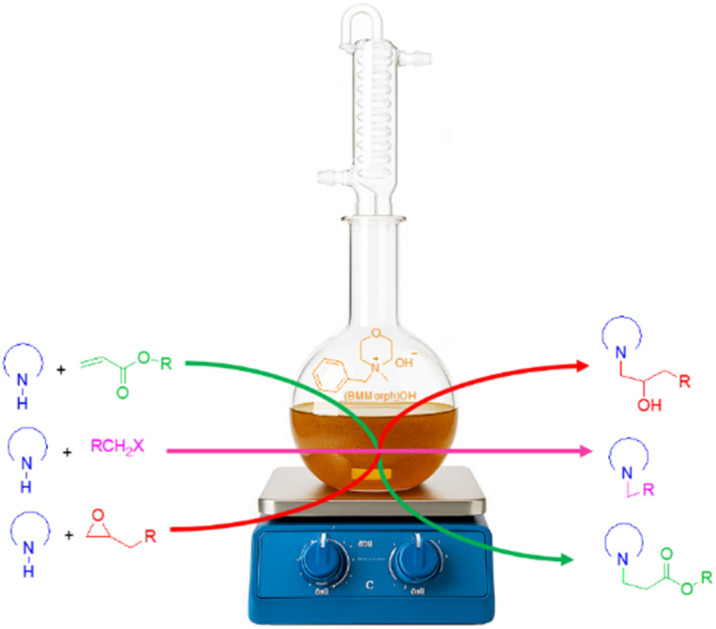

## 1. Introduction

Ionic liquids (ILs) have garnered significant attention in recent decades as innovative, eco-friendly solvents that serve as sustainable alternatives to traditional organic solvents.^1^ Their versatility enables widespread use in pharmaceuticals, catalysis, extraction and separation processes, nanomaterial synthesis, and electrochemistry.^2^ A defining characteristic of ILs is their unique chemical structure, consisting of loosely bound positively and negatively charged ions. This structure grants them high polarity, making them excellent solvents for a broad spectrum of inorganic and organic compounds.^3^ Among their most notable advantages are their negligible vapor pressure which minimizes inhalation risks, non-flammability, and tunable chemical properties. Unlike conventional organic solvents, ILs can be structurally modified by altering their cationic head groups, substituents, and anions, allowing for tailored applications.^4^ A subset of ILs, known as room-temperature ionic liquids (RTILs), remain liquid at or near ambient temperatures. Due to their low volatility and reduced environmental impact, RTILs have emerged as promising replacements for hazardous and volatile organic solvents, finding utility across diverse scientific and industrial fields.^5^

Historically, research on ionic liquids (ILs) has predominantly centered on conventional cation classes such as imidazolium, pyridinium, piperidinium, pyrrolidinium, tetraalkylammonium, phosphonium, and sulfonium, paired with various anions.^6^ Despite the extensive development and evaluation of ILs for diverse applications, significant limitations persist, hindering their broad industrial adoption. These include high production costs, toxicity and environmental risks (notably poor biodegradability), elevated viscosity, sensitivity to moisture, corrosive tendencies, challenges in recycling and recovery, limited commercial scalability, performance trade-offs, and ambiguous regulatory frameworks. Consequently, the design of novel ILs remains a critical endeavor to address these inherent drawbacks.^7^

In this context, morpholinium-based ILs have emerged as a promising alternative due to their exceptional properties and practical advantages.^8^ Their synthesis is straightforward, involving a concise reaction pathway, and the low cost of the precursor 4-methylmorpholine ensures affordability.^9^ These ILs exhibit versatile utility, serving as reaction media,^10^ organic synthesis catalysts,^11^ corrosion inhibitors,^12^ electrolytes,^13^ gel-electrolytes,^14^ micellization agents,^15^ lubricant oil stabilizers (*e.g.*, as antioxidants or heat stabilizers),^16^ and solvents for aromatic/paraffin separation.^17^ They also facilitate specialized applications such as size-controlled palladium nanoparticle synthesis *via* electrochemistry,^18^ superoxide ion generation,^19^ cellulose dissolution,^20^ and ‘Click’ Huisgen cycloaddition reactions.^21^

A key structural feature of morpholinium ILs, the oxygen atom within the morpholinium ring enhances their ability to dissociate mineral salts, particularly lithium salts,^22^ improving their performance in electrochemical systems. Furthermore, they demonstrate comparatively lower toxicity than many conventional ILs, positioning them as a more sustainable choice for industrial and academic applications.^23^

From a pH perspective, ionic liquids (ILs) can be classified according to their acidity or basicity, which greatly affects their properties and applications. They fall into four main categories: neutral ionic liquids (pH ∼7), such as those containing anions like [BF_4_]^−^, [PF_6_]^−^, or [Tf_2_N]^−^ paired with cations like imidazolium, pyridinium, or ammonium; acidic ionic liquids (pH < 7), which include Brønsted or Lewis acidic groups in the cation, anion, or both (*e.g.*, –SO_3_H, –COOH, AlCl_4_^−^, FeCl_4_^−^); basic ionic liquids (pH > 7), characterized by alkaline anions or functionalized cations, such as OH^−^, acetate, or amine groups; and amphoteric ionic liquids, which can act as either acids or bases depending on the conditions, such as amino acid-based ILs.^24^

Despite the widespread synthesis and application of ionic liquids (ILs) with neutral or acidic properties, only a limited number of basic ionic liquids containing hydroxide anions have been developed so far.^25^ The most well-known and commonly used hydroxide-based ILs include imidazolium hydroxides (*e.g.*, [BMIm][OH], [HMIm][OH], [OMIm][OH]), quaternary ammonium hydroxides (*e.g.*, TBAH, [Ch][OH], Triton B), phosphonium hydroxides (*e.g.*, [P_4444_][OH], [P_66614_][OH]), and pyrrolidinium hydroxides (*e.g.*, [BMPyrr][OH]).^26^

Although these basic ILs possess desirable properties that make them effective as both reaction media and bases in various organic transformations, their practical use is often hindered by inherent instability under basic conditions. For instance, imidazolium cations frequently studied in early IL research are highly susceptible to deprotonation at the C2 position.^27^ Similarly, acyclic ammonium cations may undergo Hofmann elimination,^28^ while pyridinium cations can degrade *via* ring-opening reactions.^29^ Phosphonium-based ILs exhibit moderate base stability but may decompose through the formation of phosphine oxides or phosphorus ylides in the presence of hydroxide ions.^30^

In this regard, the synthesis of new, task-specific basic ionic liquids (ILs) with enhanced stability is of great importance. Among the hydroxide-based ILs reported so far, only a few morpholinium-derived hydroxide ILs have been synthesized, and their application in organic transformations remains rare. These ILs, such as *N*-methylmorpholinium hydroxide ([NMM][OH]) and *N*-ethylmorpholinium hydroxide ([NEM][OH]), are typically prepared by solvating *N*-alkyl morpholine in water and are often isolated in aqueous solution form.^31^ To obtain the neat (solvent-free) form of morpholinium hydroxide ILs, anion exchange reactions-starting from *N*,*N*-dialkyl morpholinium chloride or bromide—represent a viable synthetic route.^32^

The formation of carbon–nitrogen (C–N) bonds is of paramount importance in organic chemistry and biochemistry, as it underpins the structure and function of a wide range of essential molecules. C–N bonds are fundamental components of amino acids, which are the building blocks of proteins, and nucleotides, which constitute DNA and RNA. These bonds contribute to the vast diversity of organic compounds, enabling the synthesis of pharmaceuticals, agrochemicals, dyes, and polymers. Efficient methods for forming C–N bonds allow chemists to construct complex molecules with nitrogen-containing functional groups such as amines, amides, and heterocycles, which are crucial for biological activity and industrial applications. Consequently, developing new strategies for C–N bond formation not only advances synthetic chemistry but also facilitates innovations in medicine, materials science, and biotechnology.^33^

In the realm of bioactive N-heterocycles such as purines, pyrimidines, and azoles, C–N bond formation plays a crucial role in generating diverse bioactive compounds. For instance, *N*-alkylation of purine or pyrimidine bases yields an important class of nucleosides known as acyclic nucleosides.^34^

Traditionally, this transformation is achieved by reacting N-heterocycles with carbon electrophiles such as epoxides, alkyl halides, or Michael acceptors.^35^ However, due to the high polarity of many bioactive N-heterocycles, particularly purine and pyrimidine nucleobases, their poor solubility in common organic solvents often necessitates the use of toxic solvents like DMF or DMSO.^36^ Thus, identifying safer alternative solvents for dissolving nucleobases and facilitating *N*-alkylation remains an important challenge.

**Scheme 1:**
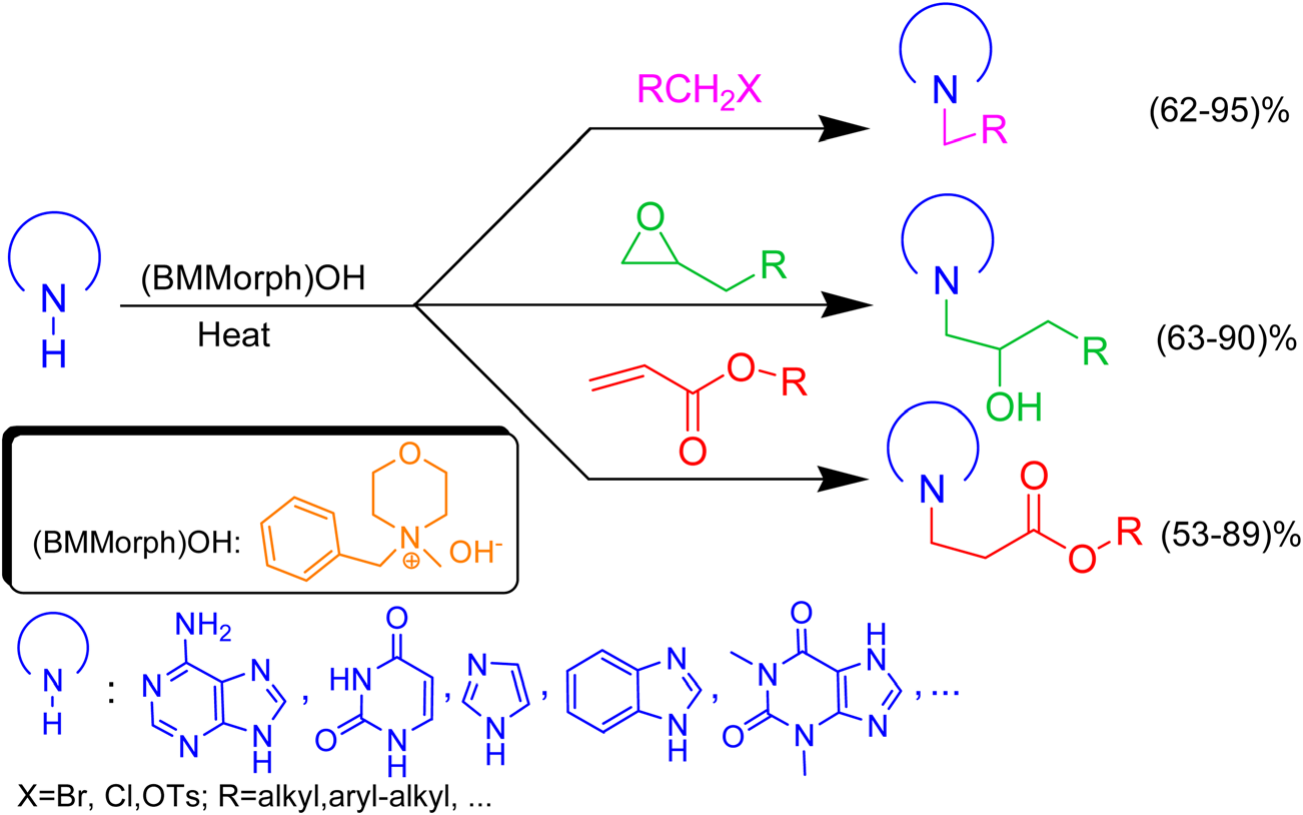
(BMMorph)OH mediated the *N*-alkylation of bioactive N-heterocycles.

In this context, we report the synthesis, characterization, and application of benzyl methyl morpholinium hydroxide ([BMMorph]OH) as a novel ionic liquid. This compound successfully serves as both a solvent and a base for the *N*-alkylation of various bioactive N-heterocycles (Scheme 1).

## 2. Results and discussion

**Scheme 2:**
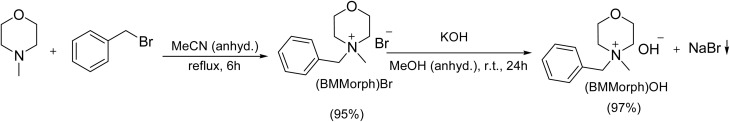
The procedure for synthesis of (BMMorph)OH.

**Fig. 1:**
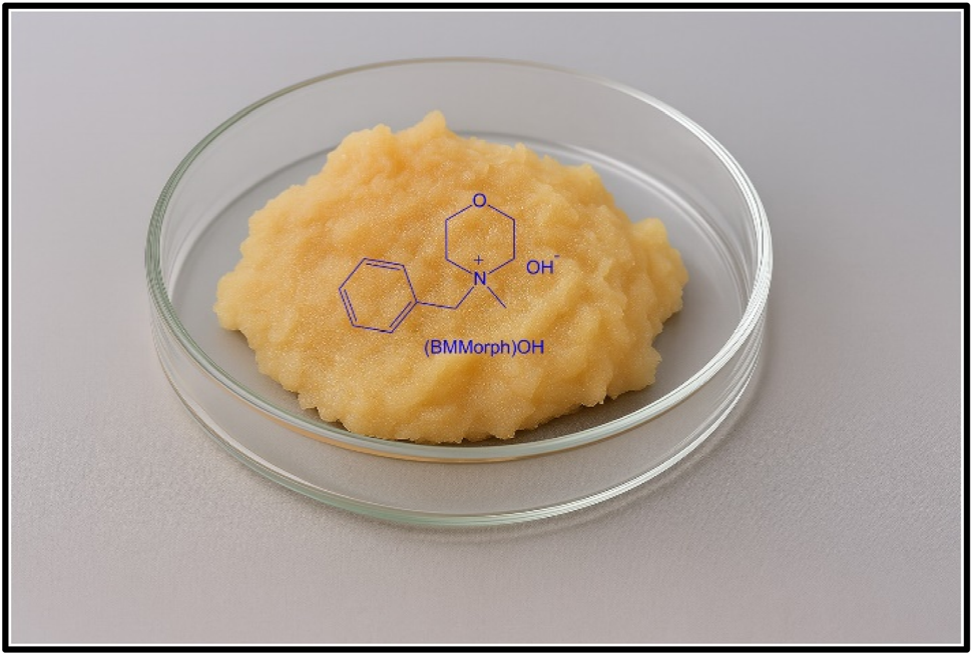
The physical status of semi-solid (BMMorph)OH.

To evaluate *N*-alkylation of bioactive N-heterocycles, we first prepared benzyl methylmorpholinium hydroxide (Scheme 2). Commercial 4-methylmorpholine was alkylated with benzyl bromide in anhydrous acetonitrile to afford 4-benzylmethylmorpholinium bromide ((BMMorph)Br) as a white solid (95%). The bromide salt was converted to the hydroxide by anion exchange with KOH in anhydrous methanol at room temperature. After removal of the precipitated KBr by filtration and solvent evaporation *in vacuo*, a yellow-brown semi-solid (BMMorph)OH was obtained (97%) (Fig. 1). The product exhibited a melting range of 88–95 °C.

**Fig. 2:**
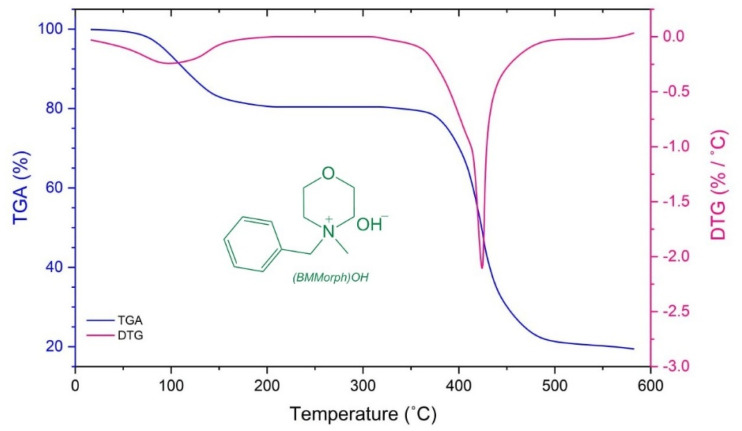
The thermogravimetric analysis of (BMMorph)OH.

Thermogravimetric analysis (TGA) of (BMMorph)OH was performed using a sample heated at a rate of 10 °C min^−1^ under a nitrogen atmosphere, across a temperature range of 20–600 °C. The resulting thermogram, presented in Fig. 2, reveals that (BMMorph)OH remains thermally stable up to approximately 150 °C, with an initial mass loss of around 20%, attributed to the evaporation of adsorbed water. Thermal decomposition begins above 370 °C, as indicated by a significant decline in mass. The endothermic events observed between 370 °C and 600 °C correspond to the progressive degradation of the compound. According to the derivative thermogravimetry (DTG) curve, the peak decomposition rate occurs at 425 °C. Overall, (BMMorph)OH exhibits a total mass loss of nearly 82% by the end of the heating cycle at 600 °C.

**Fig. 3:**
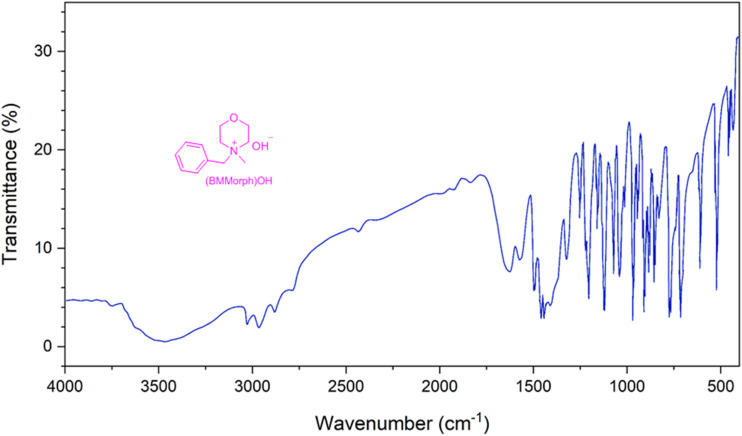
IR spectrum of (BMMorph)OH.

The IR spectrum of (BMMorph)OH is presented in Fig. 3. A broad absorption band observed in the range of 3200–3700 cm^−1^ corresponds to the presence of hydroxide ions. A medium-to-sharp peak between 3050–3100 cm^−1^ is attributed to the stretching vibrations of aromatic =C–H bonds. The complex absorption region around 2800–3000 cm^−1^ is associated with aliphatic C–H stretching, indicating the presence of CH_2_ and CH_3_ groups within the (BMMorph)OH structure. Additionally, medium to strong bands appearing in the ranges of 1600–1650 cm^−1^ and 1000–1350 cm^−1^ are assigned to C=C (aryl), C–O, and/or C–N stretching vibrations.

A set of conventional organic solvents was examined to evaluate the solubility of (BMMorph)OH (Table 1). This ionic liquid is fully soluble in protic solvents such as water, methanol, PEG200 and ethanol. Under similar conditions, it also exhibits complete solubility in solvents with high dielectric constants, including DMSO, DMF, and MeCN, whereas its solubility in acetone is moderate. In chlorinated solvents such as dichloromethane (DCM) and chloroform, the ionic liquid shows moderate solubility, while in carbon tetrachloride (CCl_4_) it is only slightly soluble. It is weakly soluble in etheric solvents such as diethyl ether and THF, and remains insoluble in non-polar or weakly polar solvents such as hexane and toluene.

**Table 1:** The solubility of (BMMorph)OH at ambient temperature (25 °C)

Entry	Solvent	Solubility^a^	Entry	Solvent	Solubility^a^
1	H_2_O	+++	9	CHCl_3_	++
2	EtOH	+++	10	CCl_4_	+
3	MeOH	+++	11	EtOAc	+
4	DMSO	+++	12	Me_2_CO	++
5	PEG 200	+++	13	PhMe	−
6	DMF	+++	14	Et_2_O	+
7	MeCN	+++	15	THF	+
8	CH_2_Cl_2_	++	16	*n*-Hexane	−

a “+++”, fully miscible or highly soluble; “++”, moderately soluble; “+”, slightly soluble; “−”, insoluble.

To assess the *N*-alkylation of bioactive N-heterocycles *via* diverse carbon electrophiles comprising alkyl halides, epoxides and Michael acceptors, the *N*-alkylation reactions of adenine with cinnamyl bromide, 2-(phenoxymethyl)oxirane and butyl acrylate was investigated as a sample reactions at different temperatures (Table 2). The reactions were carried out at different temperatures below and the above (BMMorph)OH melting point. The results are depicted in Table 2.

**Table 2:** Effect of temperature (*T* °C) on progress of sample reactions^a^

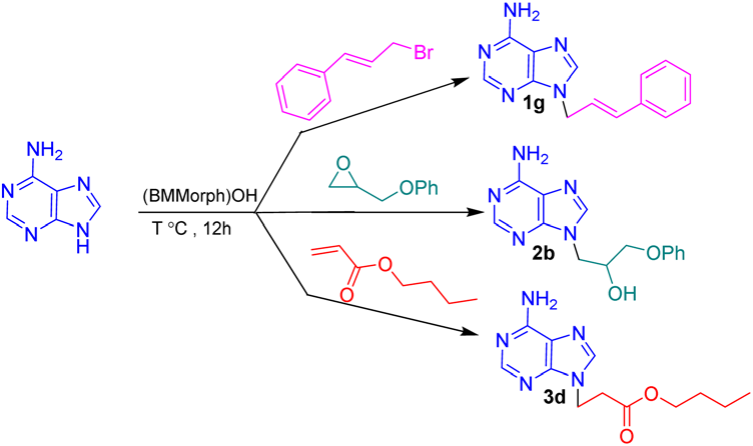				
Entry	*T* (°C)	1g^b^ (yield%)	2b^b^ (yield%)	3d^b^ (yield%)
1	30	NR^c^	NR^c^	NR^c^
2	40	NR^c^	NR^c^	NR^c^
3	50	41	32	NR^c^
4	60	52	49	33
5	70	58	54	48
6	80	63	60	52
7	90	69	65	61
8	100	76	77	74
9	110	76	77	74
10	120	76	75	72

a Reaction condition: adenine (1 mmol), cinnamyl bromide or 2-(phenoxymethyl)oxirane and/or butyl acrylate (1.2 mmol), and (BMMorph)OH (4 mol%).

b Isolated yield.

c No reaction.

As shown in Table 2, no reactions occurred between adenine and any of the carbon electrophiles below 40 °C (entries 1 and 2). Reactions with alkyl bromides and epoxides proceeded at temperatures above 50 °C, although butyl acrylate failed to react at this temperature (entry 3). When the temperature was increased further, the reaction yields improved gradually, reaching a maximum at 100 °C. No significant change in yield was observed above this temperature (entries 9 and 10). Therefore, 100 °C was selected as the optimal temperature, which also coincided with the complete melting of the (BMMorph)OH ionic liquid.

The (BMMorph)OH is not only act as a base for activation of N-heterocycles to couple with carbon electrophiles but also progress the reaction as a reaction media. Therefore, the influence of applied amount of (BMMorph)OH was assessed which the results are depicted in Table 3.

**Table 3:** Effect of IL's amount on progress of sample reactions^a^

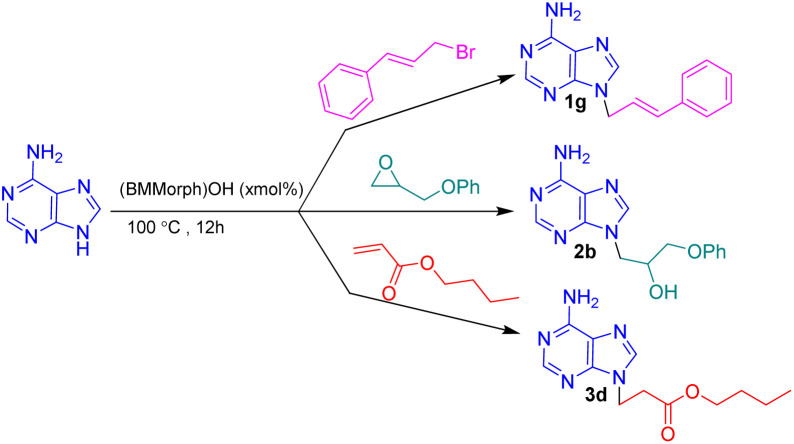				
Entry	(BMMorph)OH (mol%)	1g^b^ (yield%)	2b^b^ (yield%)	3d^a^^,^^b^ (yield%)
1	0.1	43	38	27
2	0.15	45	44	35
3	0.2	58	48	47
4	0.25	65	62	58
5	0.3	73	65	63
6	0.35	73	68	67
7	0.4	76	77	74
8	0.45	76	77	74
9	0.5	76	77	74

a Reaction condition: adenine (1 mmol), cinnamyl bromide or 2-(phenoxymethyl)oxirane and/or butyl acrylate (1.2 mmol), and (BMMorph)OH (4 mol%).

b Isolated yield.

As show in Table 3, the optimized amount of used (BMMorph)OH was also assigned to be 0.4 mol% (Table 3, entry 7). Using (BMMorph)OH less than 0.4 mol% defected in completion of all sample reactions (Table 3, entries 1–6) since the lack of homogeneity of media. Utilizing the amounts more than 0.4 mol% (BMMorph)OH has no distinguishable effect in progress of reaction (Table 3, entries 8 and 9).

A comparative evaluation of (BMMorph)[OH] was undertaken against a selection of established hydroxide ionic liquids (HILs). The study encompassed quaternary ammonium-based hydroxide ILs, including choline hydroxide ([Ch][OH]), tetrabutylammonium hydroxide (TBAH), and benzyltrimethylammonium hydroxide ([BTMA][OH], Triton B), as well as imidazolium-based hydroxide ILs such as 1-butyl-3-methylimidazolium hydroxide ([BMIm][OH]), 1-hexyl-3-methylimidazolium hydroxide ([HMIm][OH]), and 1-octyl-3-methylimidazolium hydroxide ([OMIm][OH]) (Table 4). This systematic comparison was designed to benchmark the reactivity and efficiency of (BMMorph)[OH] relative to widely available commercial alternatives.

**Table 4:** Effect of some several hydroxide ionic liquids (HILs) on progress of sample reactions^b^

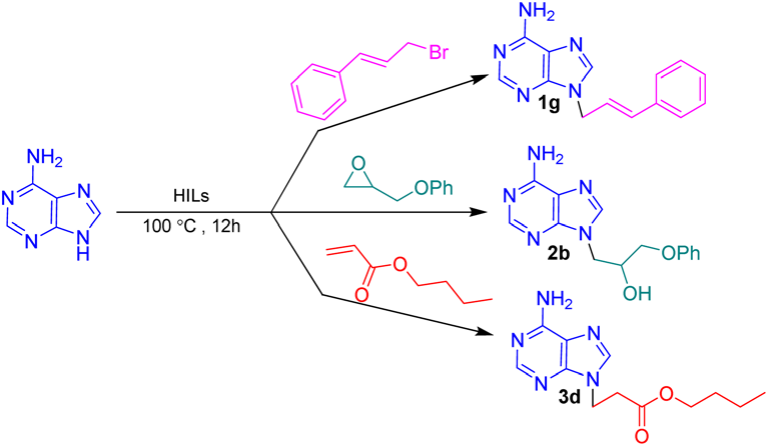				
Entry	HILs^a^	1g^c^ (yield%)	2b^c^ (yield%)	3d^c^ (yield%)
1	[Ch][OH]^d^	42	36	40
2	TBAH^d^	51	53	38
3	[BTMA][OH]^d^	68	59	44
4	[BMIm][OH]^d^	65	57	51
5	[HMIm][OH]^e^	69	61	60
6	[OMIm][OH]^e^	75	72	70
7	[BMMorph][OH]^e^	76	77	74

a HILs: 
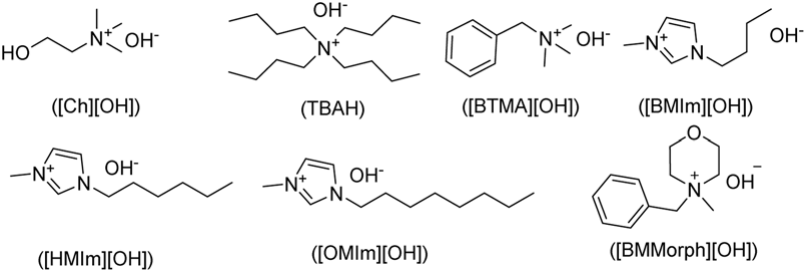
.

b Reaction condition: adenine (1 mmol), cinnamyl bromide or 2-(phenoxymethyl)oxirane and/or butyl acrylate (1.2 mmol), and HILs (4 mmol).

c Isolated yield.

d These ILs were bought and used in solution forms ([Ch][OH]: 45 wt% in MeOH; TBAH: 40 wt% in H_2_O, [BTM][OH]: 40 wt% in MeOH; [BMIm][OH]: 20wt% in EtOH).

e These ILs were used in a neat condition.

As shown in Table 4, a range of commercially available hydroxyl ionic liquids (HILs) were employed for the *N*-alkylation of adenine. Among the quaternary ammonium-based hydroxide ILs tested-namely [Ch][OH], TBAH, and [BTMA][OH] (Table 4, entries 1–3), the most favorable outcomes were consistently obtained with [BTMA][OH] across all examined carbon electrophiles. Similarly, within the series of imidazolium-based hydroxide ILs, [OMIm][OH] delivered superior performance compared to its counterparts (Table 4, entries 4–6). In general, conducting the reaction under neat conditions afforded higher yields than when the commercial HILs were diluted in a matrix (Table 4, entries 1–4). Overall, across all tested HILs, [BMMorph][OH] exhibited the most effective results for every type of carbon electrophile investigated (Table 4, entry 7).

The recovery of ionic liquids used as reaction media is critical for developing sustainable and cost-effective processes, as it reduces raw material costs and minimizes environmental impact by limiting solvent waste. This study evaluated the recovery and reusability of [BMMorph][OH] as a media over five consecutive cycles. Following each reaction, the mixture was diluted with demineralized water and extracted multiple times with chloroform in a separatory funnel. The aqueous phase was then evaporated under vacuum, and the resulting syrupy residue was dissolved in absolute ethanol, flash-filtered, and concentrated under vacuum. The recovered [BMMorph][OH] was used directly in the subsequent reaction without further purification. The results for the tested reactions are presented in Table 5.

**Table 5:** Effect of temperature on progress of sample reactions^a^

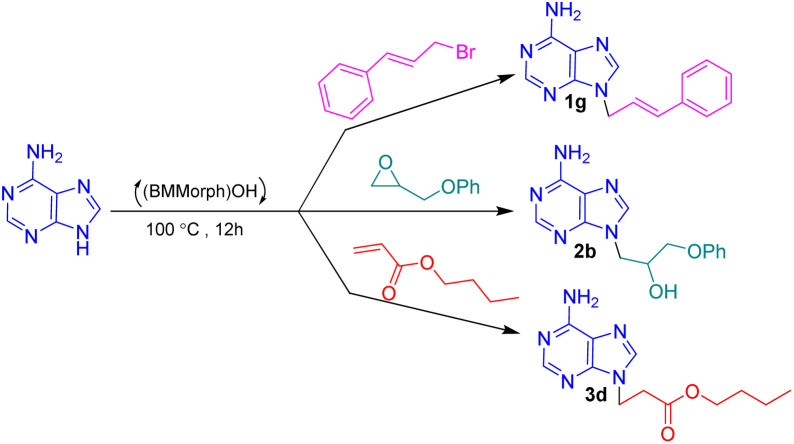				
Entry	Recovery run	1g^b^ (yield%)	2b^b^ (yield%)	3d^b^ (yield%)
1	1	76	77	74
2	2	72	74	69
3	3	68	66	61
4	4	60	61	55
5	5	57	54	50

a Reaction condition: adenine (1 mmol), cinnamyl bromide or 2-(phenoxymethyl)oxirane and/or butyl acrylate (1.2 mmol), and recycled (BMMorph)OH (4 mmol).

b Isolated yield.

As shown in Table 5, the isolated yield of the examined reactions gradually decreased with each cycle, with a more pronounced decline observed after the fourth run. This decrease is not due to structural degradation of the ionic liquid, as seen in imidazolium-based analogues. Imidazolium ionic liquids are unstable under these conditions, as the acidic C2 proton on the ring makes them susceptible to decomposition *via* N-heterocyclic carbene (NHC) formation. In contrast, [BMMorph][OH] demonstrated high thermal stability at 100 °C, which was confirmed by IR and NMR analysis of the recycled material. Instead, the yield reduction is attributed primarily to the absorption of CO_2_ from the atmosphere. This issue could be mitigated or prevented in future applications by conducting the reaction and recovery processes under an inert atmosphere.

**Fig. 4:**
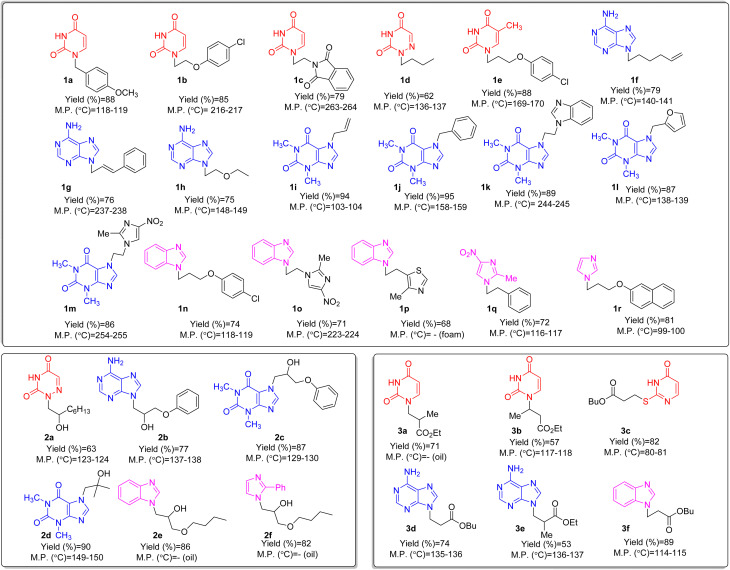
The structure of *N*-alkylated bioactive N-heterocycles comprising pyrimidine, purine and azole-based N-heterocycles *via* alkyl halides (1a–1r), epoxides (2a–2f) and Michael acceptors (3a–3f) in [BMMorph][OH].

After synthesis, characterization, and identification of the optimized reaction conditions, these conditions were applied to a broad range of bioactive N-heterocycles, including azoles (imidazole, 2-methyl-4(5)-nitroimidazole, 2-phenylimidazole, and benzimidazole), pyrimidines (uracil, thymine, azauracil, and 2-mercaptouracil), and purines (adenine and theophylline). In addition, diverse carbon electrophiles such as alkyl halides (1a–1r), epoxides (2a–2f), and Michael acceptors (3a–3f) were employed. The structures of the resulting adducts are presented in Fig. 4. As demonstrated, [BMMorph][OH] serves effectively both as the reaction medium and as the base for *N*-alkylation of bioactive N-heterocyclic compounds. Overall, reactions with carbon electrophiles such as alkyl halides and epoxides proceeded more efficiently than those with Michael acceptors under these conditions.

A longstanding challenge in nucleoside chemistry is controlling the isomeric ratio in the *N*-alkylation of adenine, which yields a mixture of N9 and N7 regioisomers due to a dynamic tautomeric equilibrium. The selective formation of one isomer is highly significant, as it would greatly simplify the separation process. It is well-established that factors such as steric and electronic effects, solvent, and counterion influence the N9/N7 ratio. Interestingly, the *N*-alkylation of adenine using [BMMorph][OH] demonstrated a strong preference for forming the thermodynamically more stable N9 isomer over the N7 isomer. To quantify this effect, we compared the N9/N7 ratios for the adduct of adenine and cinnamyl bromide synthesized *via* a traditional method (DMF/K_2_CO_3_) with those of reactions conducted in [Ch][OH] and [BMMorph][OH]. The ratios were determined using liquid chromatography (HPLC), as summarized in Table 6.

**Table 6:** N9/N7 isomeric ratio in *N*-alkylation of adenine^a^

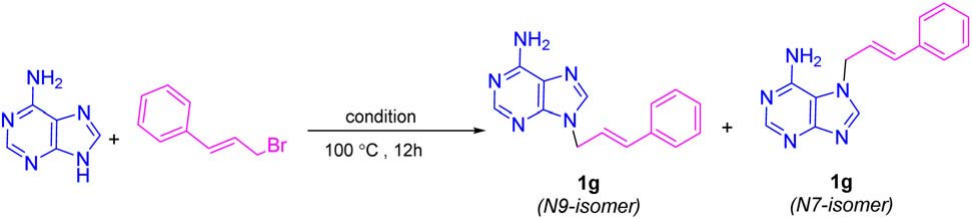		
Entry	Condition	N9/N7 (%)
1	1	95/5
2	2	72/28
3	3	61/39

a Condition 1: adenine (1 mmol), cinnamyl bromide (1.2 mmol), and (BMMorph)OH (4 mmol). Condition 2: adenine (1 mmol), cinnamyl bromide (1.2 mmol), and [Ch][OH] (45 wt% in MeOH) (4 mmol). Condition 3: adenine (1 mmol), cinnamyl bromide (1.2 mmol), K_2_CO_3_ (2 mmol), and anhydrous DMF (15 mL).

As shown in Table 6, the use of [BMMorph][OH] resulted in an excellent N9/N7 selectivity ratio, which was higher than that achieved with [Ch][OH] or the conventional DMF/K_2_CO_3_ system.

**Fig. 5:**
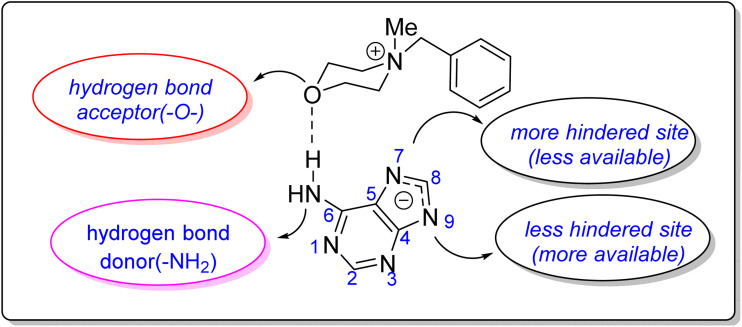
The potential effect of morpholinium's oxygen atom on N9/N7 alkylation ratio.

The high N9/N7 regioselectivity is attributed to the key role of the oxygen atom in the morpholinium cation. This oxygen is known to act as a hydrogen bond acceptor, and we propose it interacts with the NH_2_ group of adenine. This interaction stabilizes a specific transition state and sterically blocks the N7 position, making the N9 site more accessible for alkylation (Fig. 5). This proposed mechanism is consistent with previous literature, which establishes the oxygen in morpholinium-based ionic liquids not as a passive spectator, but as an active participant that can direct regioselectivity through hydrogen bonding and the stabilization of transition states.^37–40^

## 3. Conclusion

This work successfully demonstrates the synthesis and application of benzyl methyl morpholinium hydroxide ([BMMorph]OH) as a new, stable basic ionic liquid. [BMMorph]OH serves as a dual-purpose base and solvent for the efficient and regioselective *N*-alkylation of various bioactive N-heterocycles, including purines, pyrimidines, and azoles with alkyl halides, epoxides, and Michael acceptors. Notably, it exhibits superior performance compared to conventional hydroxide ionic liquids and provides excellent N9 selectivity in adenine alkylation, attributed to hydrogen-bonding interactions involving the morpholinium oxygen. The ionic liquid is recyclable for multiple runs, highlighting its potential as a sustainable and practical alternative to traditional basic media in heterocyclic chemistry particularly, in nucleoside chemistry.

## 4. Experimental

### 4.1. General chemistry

All chemicals, of analytical grade, were obtained from Merck (Germany) or Sigma-Aldrich (USA) and used as received. Reaction progress was monitored by thin-layer chromatography (TLC) on SILG/UV 254 silica-gel plates. Products were purified by flash column chromatography using silica gel 60 (70–230 mesh). Melting points were determined on an Electrothermal IA 9000 apparatus in open capillaries and are reported without correction. Elemental analysis was performed on a PerkinElmer 240-B microanalyzer. GC/MS and IR spectra were acquired using a Shimadzu GC/MS-QP 1000-EX system and a Shimadzu FT-IR-8300 spectrophotometer, respectively. NMR spectra (^1^H at 250 MHz, ^13^C at 62.5 MHz) were recorded on a Brüker Avance-DPX-250 spectrometer. Chemical shifts (*δ*) are reported relative to tetramethylsilane (TMS) as an internal standard, and coupling constants (*J*) are given in Hz. Standard abbreviations are used for NMR signal multiplicity: s (singlet), d (doublet), t (triplet), q (quartet), m (multiplet), br (broad).

#### 4.1.1. Synthesis of 4-benzylmethylmorpholinium bromide ((BMMorph)Br)

In a 50 mL round-bottom flask, *N*-methylmorpholine (1.01 g, 10 mmol) was combined with benzyl bromide (1.88 g, 11 mmol) in anhydrous acetonitrile (10 mL). The reaction mixture was stirred and refluxed for up to 6 h, with progress monitored by TLC. Upon completion, the solvent was removed under reduced pressure, and the resulting residue was diluted with distilled water (100 mL). The aqueous phase was extracted with chloroform (3 × 50 mL). The remaining aqueous layer was evaporated under vacuum to afford crude 4-benzyl-4-methylmorpholin-4-ium bromide ((BMMorph)Br). The solid was recrystallized from hot methanol to yield pure (BMMorph)Br as a white crystalline solid (2.57 g, 95%), m. p. 180–185 °C.

#### 4.1.2. Synthesis of 4-benzylmethylmorpholinium hydroxide ((BMMorph)OH)

A mixture of (BMMorph)Br (2.72 g, 10 mmol) and powdered potassium hydroxide (0.56 g, 10 mmol) in anhydrous methanol (15 mL) was vigorously stirred at room temperature in a 50 mL round-bottom flask for 24 h. The precipitated potassium bromide was removed by rapid filtration through a sintered glass funnel. The filtrate was concentrated under reduced pressure using a rotary evaporator to afford the product (BMMorph)OH as a pale yellow semisolid (2.03 g, 97%). The product was stored in a corked flask within a vacuum desiccator at ambient temperature; m. p. 88–95 °C. IR (KBr): 3464, 3035, 2976, 1625, 1463, 1129 cm^−1^. ^1^H NMR (CDCl_3_, 400 MHz) *δ*_ppm_ = 2.45 (s, 1H, OH), 3.33 (t, *J* = 5.2 Hz, 4H, 2NCH_2_), 3.40 (s, 3H, NCH_3_), 3.43 (t, *J* = 5.2 Hz, 4H, 2OCH_2_), 3.80 (s, 2H, PhCH_2_), 6.90–7.00 (m, 3H, aryl), 7.28–7.34 (m, 2H, aryl). ^13^C NMR (CDCl_3_, 100 MHz) *δ*_ppm_ = 50.01, 59.11, 62.18, 67.31, 125.65, 129.33, 130.78, 132.87. Anal. calc. for C_12_H_19_NO_2_: C, 68.87; H, 9.15; N, 6.69; found: C, 69.05; H, 9.31; N, 6.50.

#### 4.1.3. General procedure for *N*-alkylation of bioactive N-heterocycles

In a 50 mL round-bottom flask, a mixture of the bioactive N-heterocycle (1.0 mmol), the carbon electrophile (1.2 mmol; alkyl halide or epoxide, and/or Michael acceptor), and the ionic liquid (BMMorph)OH (0.84 g, 4.0 mmol) was stirred and heated at 100 °C in an oil bath for 12 h. The reaction progress was monitored by thin-layer chromatography (TLC). Upon completion, the crude mixture was dissolved in chloroform (150 mL) and rapidly filtered through a sintered glass funnel to remove insoluble solids. The filtrate was washed with deionized water (3 × 100 mL). The organic layer was dried over anhydrous sodium sulfate (10 g), filtered, and concentrated under reduced pressure. The resulting crude product was purified by flash column chromatography on silica gel using the eluent system specified for each compound.

#### 4.1.4. Recovery of (BMMorph)OH

The aqueous phase from the work-up procedure was concentrated by heating on a stirrer-hotplate to a volume of approximately 50 mL. The remaining solution was transferred and evaporated to dryness under reduced pressure using a rotary evaporator. The resulting viscous oil was dried overnight in a vacuum oven at 100 °C. The dried product was then dissolved in absolute methanol (50 mL). Powdered potassium hydroxide (0.56 g, 10 mmol) was added, and the mixture was stirred at room temperature for 24 h to regenerate the hydroxide form. The resulting precipitate, containing potassium bromide (in the case of using alkyl bromides), carbonates and/or any unreacted KOH, was removed by filtration through a sintered glass funnel. The filtrate was concentrated under reduced pressure to yield recovered (BMMorph)OH. The identity and purity of the recovered ionic liquid were confirmed by FT-IR and ^1^H NMR spectroscopy.

#### 4.1.5. Data of the synthesized compounds

##### 4.1.5.1 1-(4-Methoxybenzyl) pyrimidine-2,4(1*H*,3*H*)-dione (1a)^41^

Column chromatography on silica gel eluted with hexane/EtOAc (1 : 1) afforded pure product as white solid (88%); m. p. 118–119 °C. IR (KBr): 3250, 3100, 2895, 1728, 1715, 1456, 1248 cm^−1^. ^1^H NMR (DMSO-*d*_6_, 250 MHz) *δ*_ppm_ = 3.70 (s, 3H, OCH_3_), 4.76 (s, 2H, NCH_2_), 5.58 (d, *J* = 7.8 Hz, 1H, C(5)–H of uracil), 6.84 (d, *J* = 8.6 Hz, 2H, aryl), 7.14 (d, *J* = 8.6 Hz, 2H, aryl), 7.72 (d, *J* = 7.8 Hz, 1H, C(6)–H of uracil), 11.17 (s, 1H, NH). ^13^C NMR (DMSO-*d*_6_, 62.5 MHz) *δ*_ppm_ = 49.66, 54.99, 101.19, 113.55, 128.67, 129.12, 145.38, 150.95, 158.79, 163.62. Anal. calc. for C_12_H_12_N_2_O_3_: C, 62.06; H, 5.21; N, 12.06; found: C, 62.14; H, 5.34; N, 12.21.

##### 4.1.5.2 1-(2-(4-Chlorophenoxy)ethyl)pyrimidine-2,4(1*H*,3*H*)-dione (1b)^41^

Column chromatography on silica gel eluted with hexane/EtOAc (1 : 1) afforded pure product as white solid (85%); m. p. 216–217 °C. IR (KBr): 3200, 3041, 2949, 2871, 1725, 1712, 1492, 1236, 1039 cm^−1^. ^1^H NMR (DMSO-*d*_6_, 250 MHz) *δ*_ppm_ = 4.08 (t, *J* = 4.8 Hz, 2H, NCH_2_), 4.20 (t, *J* = 4.8 Hz, 2H, OCH_2_), 5.57 (d, *J* = 7.8 Hz, 1H, C(5)–H of uracil), 6.96 (d, *J* = 8.9 Hz, 2H, aryl), 7.32 (d, *J* = 8.9 Hz, 2H, aryl), 7.71 (d, *J* = 7.8 Hz, 1H, C(6)–H of uracil), 11.34 (s, 1H, NH). ^13^C NMR (DMSO-*d*_6_, 62.5 MHz) *δ*_ppm_ = 46.78, 65.54, 100.61, 116.18, 124.62, 129.19, 146.15, 150.90, 156.77, 163.67. Anal. calc. for C_12_H_11_ClN_2_O_3_: C, 54.05; H, 4.16; N, 10.50; found: C, 53.87; H, 4.28; N, 10.31.

##### 4.1.5.3 2-(2-(2,4-Dioxo-3,4-dihydropyrimidin-1(2*H*)-yl)ethyl)isoindoline-1,3-dione (1c)^42^

Column chromatography on silica gel with hexane/EtOAc (2 : 3) afforded pure product as white solid (79%); m. p. = 263–264 °C. IR (KBr): 3200, 3210, 2895, 1730, 1710, 1449 cm^−1^. ^1^H NMR (DMSO-*d*_6_, 250 MHz) *δ*_ppm_ = 3.83–3.87 (m, 4H, 2 NCH_2_), 5.45 (d, *J* = 7.8 Hz, 1H, C(5)–H of uracil), 7.52 (d, *J* = 7.8 Hz, 1H, C(6)–H of uracil), 7.78–7.86 (m, 4H, aryl), 11.15 (s, 1H, NH). ^13^C NMR (DMSO-*d*_6_, 62.5 MHz) *δ*_ppm_ = 36.44, 46.48, 101.09, 123.08, 131.43, 134.39, 145.47, 151.18, 163.63, 167.67. Anal. calc. for C_14_H_11_N_3_O_4_: C, 58.96; H, 3.89; N, 14.73; found: C, 59.07; H, 3.70; N, 14.85.

##### 4.1.5.4 2-Butyl-1,2,4-triazine-3,5(2*H*,4*H*)-dione (1d)^42^

Column chromatography on silica gel with hexane/EtOAc (2 : 3) afforded pure product as white solid (62%); m. p. = 136–137 °C. IR (KBr): 3200, 3210, 2985, 2895, 1725, 1715 cm^−1^. ^1^H NMR (DMSO-*d*_6_, 250 MHz) *δ*_ppm_ = 0.63 (t, *J* = 6.2 Hz, 3H, CH_3_), 1.01–1.04 (m, 2H, C*H*_2_CH_3_), 1.26–1.30 (m, 2H, NCH_2_C*H*_2_), 3.48 (t, *J* = 6.5 Hz, 2H, NCH_2_), 7.20 (s, 1H, C(5)–H of azauracil), 12.29 (s, 1H, NH). ^13^C NMR (DMSO-*d*_6_, 62.5 MHz) *δ*_ppm_ = 13.41, 19.47, 28.67, 38.32, 134.53, 148.99, 156.10. Anal. calc. for C_7_H_11_N_3_O_2_: C, 49.70; H, 6.55; N, 24.84; found: C, 49.83; H, 6.41; N, 24.97.

##### 4.1.5.5 1-(3-(4-Chlorophenoxy)propyl)-5-methylpyrimidine-2,4(1*H*,3*H*)-dione (1e)^41^

Column chromatography on silica gel eluted with hexane/EtOAc (1 : 1) afforded pure product as white solid (88%); m. p. 169–170 °C. IR (KBr): 3151, 3075, 2867, 2806, 1730, 1718, 1456, 1247, 1052 cm^−1^. ^1^H NMR (DMSO-*d*_6_, 250 MHz) *δ*_ppm_ = 1.73 (s, 3H, CH_3_), 2.03–2.10 (m, 2H, OCH_2_C*H*_2_), 3.82 (t, *J* = 6.7 Hz, 2H, NCH_2_), 4.00 (t, *J* = 5.8 Hz, 2H, OCH_2_), 6.94 (d, *J* = 8.9 Hz, 2H, aryl), 7.33 (d, *J* = 8.9 Hz, 2H, aryl), 7.62 (s, 1H, C(6)–H of thymine), 11.24 (s, 1H, NH). ^13^C NMR (DMSO-*d*_6_, 62.5 MHz) *δ*_ppm_ = 11.83, 27.83, 44.94, 65.36, 108.34, 116.05, 124.21, 129.09, 141.49, 150.87, 157.14, 164.26. Anal. calc. for C_14_H_15_ClN_2_O_3_: C, 57.05; H, 5.13; N, 9.50; found: C, 57.18; H, 5.29; N, 9.71.

##### 4.1.5.6 9-(Hex-5-enyl)-9*H*-purin-6-amine (1f)^41^

Column chromatography on silica gel eluted with EtOAc afforded pure product as white solid (79%); m. p. 140–141 °C. IR (KBr): 3330, 3115, 2948, 1435 cm^−1^. ^1^H NMR (DMSO-*d*_6_, 250 MHz) *δ*_ppm_ = 1.31–1.40 (m, 2H, CH_2_), 1.81–1.90 (m, 2H, CH_2_), 2.00–2.09 (m, 2H, CH_2_), 4.20 (t, *J* = 7.0 Hz, 2H, NCH_2_), 4.93 (dd, *J* = 1.3, 9.2 Hz, 2H, =CH_2_), 5.69–5.85 (m, 1H, =CH), 7.32 (br s, 2H, NH_2_), 8.22 (s, 1H, C(2)–H of adenine), 8.25 (s, 1H, C(8)–H of adenine). ^13^C NMR (DMSO-*d*_6_, 62.5 MHz) *δ*_ppm_ = 25.18, 29.07, 32.45, 42.64, 114.85, 118.68, 138.15, 140.76, 149.48, 152.31, 155.90. Anal. calc. for C_11_H_15_N_5_: C, 60.81; H, 6.96; N, 32.23; found: C, 60.94; H, 7.08; N, 32.10.

##### 4.1.5.7 (*E*)-9-Cinnamyl-9*H*-purin-6-amine (1g)^41^

Column chromatography on silica gel eluted with EtOAc afforded pure product as pale yellow solid (76%); m. p. 237–238 °C. IR (KBr): 3355, 3130, 2950, 1483 cm^−1^. ^1^H NMR (DMSO-*d*_6_, 250 MHz) *δ*_ppm_ = 4.93 (d, *J* = 3.8 Hz, 2H, NCH_2_), 6.43 (d, *J* = 16.4 Hz, 1H, =C*H*Ph), 7.21–7.31 (complex, 6H, CH_2_C*H*, aryl), 7.37 (s, 1H, C(2)–H, adenine), 7.40 (s, 2H, NH_2_), 8.15 (s, 1H, C(8)–H, adenine). ^13^C NMR (DMSO-*d*_6_, 62.5 MHz) *δ*_ppm_ = 44.50, 118.63, 124.56, 126.38, 127.85, 128.57, 132.31, 135.80, 140.58, 149.35, 152.49, 155.93. Anal. calc. for C_14_H_13_N_5_: C, 66.92; H, 5.21; N, 27.87; found: C, 66.80; H, 5.34; N, 27.98.

##### 4.1.5.8 9-(2-Ethoxyethyl)-9*H*-purin-6-amine (1h)^42^

Column chromatography on silica gel eluted with EtOAc afforded pure product as with solid (75%); m. p. 148–149 °C. IR (KBr): 3310, 3160, 2985, 1462, 1228 cm^−1^. ^1^H NMR (DMSO-*d*_6_, 250 MHz) *δ*_ppm_ = 0.98 (t, *J* = 6.9 Hz, 3H, CH_3_), 3.36 (q, *J* = 6.9 Hz, 2H, CH_3_C*H*_2_), 3.69 (t, *J* = 5.2 Hz, 2H, NCH_2_), 4.27 (t, *J* = 5.2 Hz, 2H, OCH_2_), 7.24 (s, 2H, NH_2_), 8.06 (s, 1H, C(2)–H, adenine), 8.13 (s, 1H, C(8)–H, adenine). ^13^C NMR (DMSO-*d*_6_, 62.5 MHz) *δ*_ppm_ = 14.82, 42.77, 65.29, 67.55, 118.51, 141.12, 149.44, 152.31, 155.86. Anal. calc. for C_9_H_13_N_5_O: C, 52.16; H, 6.32; N, 33.79; found: C, 52.29; H, 6.20; N, 33.86.

##### 4.1.5.9 7-Allyl-1,3-dimethyl-1*H*-purine-2,6(3*H*,7*H*)-dione (1i)^41^

Column chromatography on silica gel eluted with hexane/EtOAc (1 : 1) afforded pure product as white solid (94%); m. p. 103–104 °C. IR (KBr): 3050, 2987, 2890, 1725, 1708, 1473 cm^−1^. ^1^H NMR (DMSO-*d*_6_, 250 MHz) *δ*_ppm_ = 3.17 (s, 3H, N(3)–CH_3_), 3.36 (s, 3H, N(1)–CH_3_), 4.73 (d, *J* = 5.2 Hz, 2H, NCH_2_), 5.00–5.13 (dd, *J* = 11.5, 16.4 Hz, 2H, =CH_2_), 5.77–5.93 (m, 1H, =CH), 7.40 (s, 1H, C(8)–H of theophylline). ^13^C NMR (DMSO-*d*_6_, 62.5 MHz) *δ*_ppm_ = 27.80, 29.61, 48.83, 106.71, 119.18, 132.06, 140.71, 148.64, 151.50, 154.95. Anal. calc. for C_10_H_12_N_4_O_2_: C, 54.54; H, 5.49; N, 25.44; found: C, 54.68; H, 5.58; N, 25.30.

##### 4.1.5.10 7-Benzyl-1,3-dimethyl-1*H*-purine-2,6(3*H*,7*H*)-dione (1j)^41^

Column chromatography on silica gel eluted with hexane/EtOAc (1 : 1) afforded pure product as white solid (95%); m. p. 158–159 °C. IR (KBr): 3100, 2980, 2895, 1720, 1705, 1471 cm^−1^. ^1^H NMR (DMSO-*d*_6_, 250 MHz) *δ*_ppm_ = 3.31 (s, 3H, N(3)–CH_3_), 3.49 (s, 3H, N(1)–CH_3_), 5.42 (s, 2H, NCH_2_), 7.12–7.31 (m, 5H, aryl), 7.54 (s, 1H, C(8)–H of theophylline). ^13^C NMR (DMSO-*d*_6_, 62.5 MHz) *δ*_ppm_ = 27.92, 29.68, 50.16, 106.87, 127.37, 127.88, 128.68, 135.39, 140.89, 148.77, 151.55, 155.14. Anal. calc. for C_14_H_14_N_4_O_2_: C, 62.21; H, 5.22; N, 20.73; found: C, 62.35; H, 5.31; N, 20.61.

##### 4.1.5.11 7-(2-(1*H*-Benzo[*d*]imidazole-1-yl)ethyl)-1,3-dimethyl-1*H*-purine-2,6(3*H*,7*H*)-dione (1k)^42^

Column chromatography on silica gel eluted with MeOH/EtOAc (1 : 14) afforded pure product as pale yellow solid (89%); m. p. 244–245 °C. IR (KBr): 3094, 2952, 1718, 1702, 1479 cm^−1^. ^1^H NMR (CDCl_3_, 250 MHz) *δ*_ppm_ = 3.44 (s, 3H, N(3)–CH_3_), 3.54 (s, 3H, N(1)–CH_3_), 4.67–4.71 (m, 4H, NCH_2_CH_2_N), 6.98 (s, 1H, C(2)–H, benzimidazole), 7.28–7.42 (m, 4H, aryl), 7.59 (s, 1H, C(8)–H, theophylline). ^13^C NMR (CDCl_3_, 62.5 MHz) *δ*_ppm_ = 29.49, 31.45, 50.31, 56.20, 105.97, 115.15, 117.16, 124.34, 126.26, 133.06, 136.13, 142.67, 146.17, 149.65, 152.05, 155.74. Anal. calc. for C_16_H_16_N_6_O_2_: C, 59.25; H, 4.97; N, 25.91; found: C, 59.37; H, 5.06; N, 25.98.

##### 4.1.5.12 7-(Furan-2-ylmethyl)-1,3-dimethyl-1*H*-purine-2,6(3*H*,7*H*)-dione (1l)^42^

Column chromatography on silica gel eluted with hexane/EtOAc (3 : 1) afforded pure product as bright brown solid (87%); m. p. 138–139 °C. IR (KBr): 3086, 2957, 1717, 1701, 1546, 1473 cm^−1^. ^1^H NMR (CDCl_3_, 250 MHz) *δ*_ppm_ = 3.31 (s, 3H, N(3)–CH_3_), 3.47 (s, 3H, N(1)–CH_3_), 5.42 (s, 2H, NCH_2_), 6.26 (br s, 1H, C(3)–H of furan), 6.43 (br s, 1H, C(4)–H of furan), 7.31 (br s, 1H, C(5)–H of furan), 7.56 (s, 1H, C(8)–H of theophylline). ^13^C NMR (CDCl_3_, 62.5 MHz) *δ*_ppm_ = 29.26, 32.21, 44.74, 105.12, 107.58, 112.09, 141.97, 143.85, 148.68, 150.64, 156.24, 158.01. Anal. calc. for C_12_H_12_N_4_O_3_: C, 55.38; H, 4.65; N, 21.53; found: C, 55.47; H, 4.76; N, 21.62.

##### 4.1.5.13 1,3-Dimethyl-7-(2-(2-methyl-4-nitro-1*H*-imidazol-1-yl)ethyl)-1*H*-purine-2,6(3*H*,7*H*)-dione (1m)^42^

Recrystallization from MeOH/EtOAc afforded pure product as white solid (86%); m. p. 254–255 °C. IR (KBr): 3109, 2954, 1720, 1706, 1542, 1473, 1336 cm^−1^. ^1^H NMR (DMSO-*d*_6_, 250 MHz) *δ*_ppm_ = 2.19 (s, 3H, C(2)–CH_3_, imidazole), 3.19 (s, 3H, N(3)–CH_3_), 3.38 (s, 3H, N(1)–CH_3_), 4.54–4.59 (m, 4H, NCH_2_CH_2_N), 7.81 (s, 1H, C(5)–H of imidazole), 8.04 (s, 1H, C(8)–H of theophylline). ^13^C NMR (DMSO-*d*_6_, 62.5 MHz) *δ*_ppm_ = 15.11, 27.66, 31.61, 49.62, 52.08, 105.30, 121.11, 140.19, 146.76, 149.11, 151.06, 153.20, 156.29. Anal. calc. for C_13_H_15_N_7_O_4_: C, 46.85; H, 4.54; N, 29.42; found: C, 46.98; H, 4.67; N, 29.58.

##### 4.1.5.14 1-(3-(4-Chloro phenoxy)propyl)-1*H*-benzo[*d*]imidazole (1n)^42^

Column chromatography on silica gel eluted with EtOAc afforded pure product as pale yellow solid (74%); m. p. 118–119 °C. IR (KBr): 3160, 3100, 2985, 2890, 1460, 774 cm^−1^. ^1^H NMR (CDCl_3_, 250 MHz) *δ*_ppm_ = 2.25–2.28 (m, 2H, CH_2_), 3.79 (t, *J* = 6.1 Hz, 2H, NCH_2_), 4.35 (t, *J* = 6.1 Hz, 2H, OCH_2_), 6.73 (d, *J* = 7.7 Hz, 2H, aryl), 7.20 (d, *J* = 7.7 Hz, 2H, aryl), 7.21–7.35 (m, 4H, aryl), 7.81 (s, 1H, C(2)–H of benzimidazole). ^13^C NMR (CDCl_3_, 62.5 MHz) *δ*_ppm_ = 29.33, 41.35, 64.10, 109.53, 115.68, 120.43, 122.16, 122.99, 125.97, 129.42, 133.70, 143.16, 143.88, 156.97. Anal. calc. for C_16_H_15_ClN_2_O: C, 67.02; H, 5.27; N, 9.77; found: C, 67.17; H, 5.41; N, 9.85.

##### 4.1.5.15 1-(2-(2-Methyl-4-nitro-1*H*-imidazol-1-yl) ethyl)-1*H*-benzo[*d*]imidazole (1o)^42^

Column chromatography on silica gel eluted with EtOAc afforded pure product as pale yellow solid (71%); m. p. 223–224 °C. IR (KBr): 3210, 3100, 2980, 2895, 1532, 1472, 1357 cm^−1^. ^1^H NMR (DMSO-*d*_6_, 250 MHz) *δ*_ppm_ = 1.82 (s, 3H, CH_3_), 4.43 (t, *J* = 6.2 Hz, 2H, NCH_2_), 4.68 (t, *J* = 6.2 Hz, 2H, NCH_2_), 7.17–7.20 (m, 2H, aryl), 7.49 (d, *J* = 7.5 Hz, 1H, aryl), 7.65 (d, *J* = 7.5 Hz, 1H, aryl), 8.01 (s, 1H, C(5)–H of imidazole), 8.16 (s, 1H, C(2)–H of benzimidazole). ^13^C NMR (DMSO-*d*_6_, 62.5 MHz) *δ*_ppm_ = 12.03, 44.03, 46.19, 109.83, 119.48, 121.98, 122.19, 122.52, 133.51, 143.15, 143.84, 145.01, 145.46. Anal. calc. for C_13_H_13_N_5_O_2_: C, 57.56; H, 4.83; N, 25.82; found: C, 57.70; H, 4.94; N, 25.98.

##### 4.1.5.16 5-(2-(1*H*-Benzo[*d*]imidazol-1-yl)ethyl)-4-methylthiazole (1p)^42^

Column chromatography on silica gel eluted with MeOH/EtOAc (1 : 14) afforded pure product as bright brown foam (68%). IR (liquid film): 3076, 2984, 1698, 1459, 1282 cm^−1^. ^1^H NMR (CDCl_3_, 400 MHz) *δ*_ppm_ = 1.97 (s, 3H, CH_3_), 3.15 (t, *J* = 6.4 Hz, 2H, NCH_2_C*H*_2_), 4.22 (t, *J* = 6.4 Hz, 2H, NCH_2_), 7.17–7.22 (m, 3H, aryl) 7.55 (s, 1H, C(2)–H of benzimidazole), 7.68–7.70 (m, 1H, aryl), 8.44 (s, 1H, C(2)–H of thiazole). ^13^C NMR (CDCl_3_, 100 MHz) *δ*_ppm_ = 15.59, 27.81, 53.09, 108.92, 120.32, 122.53, 123.91, 128.39, 133.93, 144.20, 144.49, 148.08, 151.97. Anal. calc. for C_13_H_13_N_3_S: C, 64.17; H, 5.39; N, 17.27; found: C, 64.25; H, 5.47; N, 17.19.

##### 4.1.5.17 2-Methyl-4-nitro-1-phenethyl-1*H*-imidazole (1q)^42^

Column chromatography on silica gel eluted with EtOAc afforded pure product as white solid (72%); m. p. 116–117 °C. IR (KBr): 3135, 2950, 2878, 1552, 1485, 1354 cm^−1^. ^1^H NMR (CDCl_3_, 250 MHz) *δ*_ppm_ = 2.05 (s, 3H, CH_3_), 2.99 (t, *J* = 6.0 Hz, 2H, PhCH_2_), 4.10 (t, *J* = 6.0 Hz, 2H, NCH_2_), 6.95–7.16 (m, 5H, aryl), 7.52 (s, 1H, C(5)–H of imidazole). ^13^C NMR (CDCl_3_, 62.5 MHz) *δ*_ppm_ = 12.64, 36.88, 48.66, 119.71, 127.39, 128.62, 128.98, 136.29, 145.01, 146.23. Anal. calc. for C_12_H_13_N_3_O_2_: C, 62.33; H, 5.67; N, 18.17; found: C, 62.46; H, 5.79; N, 18.28.

##### 4.1.5.18 1-(3-(Naphthalen-2-yloxy)propyl)-1*H*-imidazole (1r)^41^

Column chromatography on silica gel eluted with EtOAc afforded pure product as white solid (81%); m. p. 99–100 °C. IR (KBr): 3150, 2948, 2887, 1456, 1228 cm^−1^. ^1^H NMR (CDCl_3_, 250 MHz) *δ*_ppm_ = 2.15–2.25 (m, 2H, CH_2_), 3.93 (t, *J* = 5.6 Hz, 2H, NCH_2_), 4.11 (t, *J* = 5.6 Hz, 2H, OCH_2_), 6.89 (s, 1H, C(5)–H of imidazole), 7.06 (s, 1H, C(4)–H of imidazole), 7.12–7.15 (m, 2H, aryl), 7.30–7.40 (m, 2H, aryl), 7.46 (s, 1H, C(2)–H of imidazole), 7.68 (s, 1H, aryl), 7.72–7.76 (m, 2H, aryl). ^13^C NMR (CDCl_3_, 62.5 MHz) *δ*_ppm_ = 30.71, 43.45, 63.74, 106.74, 118.59, 119.02, 123.84, 126.51, 126.77, 127.66, 129.10, 129.57, 129.63, 134.46, 137.31, 156.37. Anal. calc. for C_16_H_16_N_2_O: C, 76.16; H, 6.39; N, 11.10; found: C, 76.05; H, 6.50; N, 11.21.

##### 4.1.5.19 2-(2-Hydroxyoctyl)-1,2,4-triazine-3,5(2*H*,4*H*)-dione (2a)^42^

Column chromatography on silica gel eluted with hexane/EtOAc (6 : 4) afforded pure product as white solid (63%); m. p. 123–124 °C. IR (KBr): 3600–3200, 3100, 2895, 1730, 1700, 1650, 1250 cm^−1^. ^1^H NMR (DMSO-*d*_6_, 250 MHz) *δ*_ppm_ = 0.85 (t, *J* = 6.3 Hz, 3H, CH_3_), 1.24–1.34 (m, 10H, (CH_2_)_5_), 3.56 (dd, *J* = 2.5, 11.3 Hz, 2H, NCH_2_), 3.79–3.87 (m, 1H, C*H*OH), 4.75 (d, *J* = 4.4 Hz, 1H, OH), 7.46 (s, 1H, C(5)–H of azauracil), 12.48 (s, 1H, NH). ^13^C NMR (DMSO-*d*_6_, 62.5 MHz) *δ*_ppm_ = 14.93, 22.18, 25.04, 28.89, 32.68, 35.19, 45.09, 66.39, 135.06, 150.00, 156.39. Anal. calc. for C_11_H_19_N_3_O_3_: C, 54.76; H, 7.94; N, 17.41; found: C, 54.89; H, 8.05; N, 17.56.

##### 4.1.5.20 1-(6-Amino-9*H*-purin-9-yl)-3-phenoxypropan-2-ol (2b)^41^

Column chromatography on silica gel eluted with hexane/EtOAc (2 : 1) afforded pure product as white solid (77%); m. p. 137–138 °C. IR (KBr): 3500, 3350, 3090, 2974, 1467, 1238 cm^−1^. ^1^H NMR (DMSO-*d*_6_, 250 MHz) *δ*_ppm_ = 4.14 (dd, *J* = 6.2, 10.2 Hz, 1H, NC*H*_A_H_B_), 4.30 (dd, *J* = 3.7, 10.2 Hz, 1H, NCH_A_*H*_B_), 4.38–4.44 (m, 1H, C*H*OH), 4.47 (s, 1H, OH), 4.60–4.65 (m, 2H, OCH_2_), 7.14 (br s, 2H, NH_2_), 7.45–7.51 (m, 5H, aryl), 8.30 (s, 1H, C(2)–H of adenine), 8.42 (s, 1H, C(8)–H of adenine). ^13^C NMR (DMSO-*d*_6_, 62.5 MHz) *δ*_ppm_ = 46.72, 69.48, 70.07, 114.64, 119.09, 121.11, 129.85, 141.92, 150.05, 152.76, 156.32, 158.56. Anal. calc. for C_14_H_15_N_5_O_2_: C, 58.94; H, 5.30; N, 24.55; found: C, 59.06; H, 5.19; N, 24.69.

##### 4.1.5.21 7-(2-Hydroxy-3-phenoxypropyl)-1,3-dimethyl-1*H*-purine-2,6(3*H*,7*H*)-dione (2c)^41^

Column chromatography on silica gel eluted with hexane/EtOAc (2 : 1) afforded pure product as white solid (87%); m. p. 129–130 °C. IR (KBr): 3500, 3100, 2943, 1725, 1710, 1462, 1303, 1055 cm^−1^. ^1^H NMR (CDCl_3_, 250 MHz) *δ*_ppm_ = 3.37 (s, 3H, N(3)–CH_3_), 3.56 (s, 3H, N(1)–CH_3_), 4.07 (dd, *J* = 3.5, 13.4 Hz, 2H, NCH_2_), 4.18 (s, 1H, OH), 4.41–4.51 (complex, 2H, OC*H*_A_H_B_, C*H*OH), 4.65 (dd, *J* = 2.5, 13.4 Hz, 1H, OCH_A_*H*_B_), 6.87–7.01 (m, 3H, aryl), 7.25–7.32 (m, 2H, aryl), 7.66 (s, 1H, C(8)–H of theophylline). ^13^C NMR (CDCl_3_, 62.5 MHz) *δ*_ppm_ = 27.96, 29.76, 49.65, 68.68, 68.82, 106.97, 114.35, 121.36, 129.49, 142.66, 148.75, 151.29, 155.59, 158.04. Anal. calc. for C_16_H_18_N_4_O_4_: C, 58.17; H, 5.49; N, 16.96; found: C, 58.29; H, 5.36; N, 17.08.

##### 4.1.5.22 7-(2-Hydroxy-2-methylpropyl)-1,3-dimethyl-1*H*-purine-2,6(3*H*,7*H*)-dione (2d)

Column chromatography on silica gel eluted with hexane/EtOAc (1 : 1) afforded pure product as white solid (90%); m. p. 149–150 °C. IR (KBr): 3450, 3075, 2968, 1710, 1698, 1494, 1283, 1050 cm^−1^. ^1^H NMR (CDCl_3_, 300 MHz) *δ*_ppm_ = 1.05 (s, 6H, C(CH_3_)_2_), 3.18 (s, 3H, N(3)–CH_3_), 3.39 (s, 3H, N(1)–CH_3_), 4.20 (s, 2H, NCH_2_), 4.86 (s, 1H, OH), 7.87 (s, 1H, C(8)–H of theophylline). ^13^C NMR (CDCl_3_, 75 MHz) *δ*_ppm_ = 27.77, 29.92, 31.43, 65.88, 70.13, 106.59, 142.02, 149.40, 152.60, 155.84. Anal. calc. for C_11_H_16_N_4_O_3_: C, 52.37; H, 6.39; N, 22.21; found: C, 52.51; H, 6.56; N, 22.37.

##### 4.1.5.23 1-(1*H*-Benzo[*d*]imidazol-1-yl)-3-butoxypropan-2-ol (2e)

Column chromatography on silica gel eluted with hexane/EtOAc (1 : 3) afforded pure product as yellow oil (86%). IR (liquid film): 3400, 3050, 2959, 1473, 1295, 1081 cm^−1^. ^1^H NMR (CDCl_3_, 300 MHz) *δ*_ppm_ = 0.90 (t, *J* = 7.5 Hz, 3H, CH_3_), 1.35–1.45 (m, 2H, *CH*_2_CH_3_), 1.54–1.63 (m, 2H, OCH_2_*CH*_2_), 3.42–3.47 (m, 4H, CH_2_OCH_2_), 3.79 (s, 1H, OH), 4.11–4.17 (m, 2H, NCH_2_), 4.27–4.34 (m, 1H, *CH*OH), 7.08–7.50 (complex, 4H, aryl), 7.73 (s, 1H, C(2)–H of benzimidazole). ^13^C NMR (CDCl_3_, 75 MHz) *δ*_ppm_ = 15.23, 20.86, 32.37, 57.01, 68.04, 69.45, 73.22, 111.49, 119.46, 123.45, 124.36, 134.93, 144.79, 145.51. Anal. calc. for C_14_H_20_N_2_O_2_: C, 67.71; H, 8.12; N, 11.28; found: C, 67.56; H, 8.01; N, 11.20.

##### 4.1.5.24 1-Butoxy-3-(2-phenyl-1*H*-imidazol-1-yl)propan-2-ol (2f)

Column chromatography on silica gel eluted with hexane/EtOAc (1 : 3) afforded pure product as yellow oil (82%). IR (liquid film): 3425, 3068, 2947, 1480, 1273, 1078 cm^−1^. ^1^H NMR (CDCl_3_, 300 MHz) *δ*_ppm_ = 0.83 (t, *J* = 7.2 Hz, 3H, CH_3_), 1.22–1.32 (m, 2H, *CH*_2_CH_3_), 1.39–1.48 (m, 2H, OCH_2_*CH*_2_), 3.23–3.31 (complex, 4H, CH_2_OCH_2_), 3.90–3.96 (m, 2H, NCH_2_), 4.07–4.16 (m, 1H, *CH*OH), 5.20 (s, 1H, OH), 6.94 (d, *J* = 6.9 Hz, 1H, C(5)–H of imidazole), 7.06 (d, *J* = 7.2 Hz, 1H, C(4)–H of imidazole), 7.33–7.35 (m, 3H, aryl), 7.51–7.52 (m, 2H, aryl). ^13^C NMR (CDCl_3_, 75 MHz) *δ*_ppm_ = 15.63, 20.15, 33.34, 51.16, 69.15, 70.78, 74.49, 152.17, 127.39, 128.28, 129.04, 130.94, 131.91, 154.31. Anal. calc. for C_16_H_22_N_2_O_2_: C, 70.04; H, 8.08; N, 10.21; found: C, 70.21; H, 8.23; N, 10.09.

##### 4.1.5.25 3-(2,4-Dioxo-3,4-dihydro-2*H*-pyrimidine-1-yl)-2-methyl-propionic acid ethyl ester (3a)^43^

Column chromatography on silica gel eluted with hexane/EtOAc (1 : 2) afforded pure product as yellow oil (71%). IR (liquid film): 3179, 3060, 2953, 1732, 1686, 1641 cm^−1^. ^1^H NMR (CDCl_3_, 250 MHz) *δ*_ppm_ = 1.14–1.19 (complex, 6H, CH_2_C*H*_3_ and CHC*H*_3_), 2.92–3.00 (m, 1H, C*H*CH_3_), 3.67 (dd, *J* = 9.5, 13.4 Hz, 1H, NC*H*_A_H_B_), 3.87 (dd, *J* = 4.5, 13.4 Hz, 1H, NCH_A_*H*_B_), 4.10 (q, *J* = 6.9 Hz, 2H, C*H*_2_CH_3_), 5.61 (d, *J* = 7.9 Hz, 1H, C(5)–H of uracil), 7.25 (d, *J* = 7.9 Hz, 1H, C(6)–H of uracil), 10.23 (s, 1H, NH). ^13^C NMR (CDCl_3_, 62.5 MHz) *δ*_ppm_ = 14.44, 15.47, 39.01, 51.98, 61.48, 102.00, 145.00, 151.59, 164.81, 174.89. Anal. calc. for C_10_H_14_N_2_O_4_: C, 53.09; H, 6.24; N, 12.38; found: C, 53.20; H, 6.37; N, 12.21.

##### 4.1.5.26 3-(2,4-Dioxo-3,4-dihydro-2*H*-pyrimidine-1-yl)-butyric acid ethyl ester (3b)^43^

Column chromatography on silica gel eluted with hexane/EtOAc (1 : 2) afforded pure product as white solid (57%); m. p. 117–118 °C. IR (KBr): 3149, 3092, 2934, 1730, 1686, 1654 cm^−1^. ^1^H NMR (CDCl_3_, 250 MHz) *δ*_ppm_ = 1.19 (t, *J* = 7.0 Hz, 3H, CH_2_C*H*_3_), 1.43 (d, *J* = 6.9 Hz, 3H, CHC*H*_3_), 2.63 (dd, *J* = 5.8, 16.2 Hz, 1H, C*H*_A_H_B_CO_2_), 2.85 (dd, *J* = 7.9, 16.2 Hz, 1H, CH_A_*H*_B_CO_2_), 4.07 (q, *J* = 7.0 Hz, 2H, C*H*_2_CH_3_), 4.68–4.82 (m, 1H, CH_3_C*H*), 5.67 (d, *J* = 7.9 Hz, 1H, C(5)–H of uracil), 7.23 (d, *J* = 7.9 Hz, 1H, C(6)–H of uracil), 9.70 (s, 1H, NH). ^13^C NMR (CDCl_3_, 62.5 MHz) *δ*_ppm_ = 14.47, 19.21, 39.32, 51.66, 61.40, 102.49, 142.77, 151.18, 164.06, 170.64. Anal. calc. for C_10_H_14_N_2_O_4_: C, 53.09; H, 6.24; N, 12.38; found: C, 52.97; H, 6.31; N, 12.50.

##### 4.1.5.27 Butyl 3-(6-oxo-1,6-dihydropyrimidin-2-ylthio)propanoate (3c)^41^

Column chromatography on silica gel eluted with hexane/EtOAc (1 : 1) afforded pure product as white solid (82%); m. p. 80–81 °C. IR (KBr): 3200, 3050, 2895, 1750, 1725, 1710, 1300, 1240 cm^−1^. ^1^H NMR (DMSO-*d*_6_, 250 MHz) *δ*_ppm_ = 0.83 (t, *J* = 7.2 Hz, 3H, CH_3_), 1.25–1.34 (m, 2H, CH_3_C*H*_2_), 1.47–1.55 (m, 2H, OCH_2_C*H*_2_), 2.71 (t, *J* = 6.6 Hz, 2H, SCH_2_C*H*_2_), 3.26 (t, *J* = 6.6 Hz, 2H, SCH_2_), 4.00 (t, *J* = 6.4 Hz, 2H, OCH_2_), 6.08 (d, *J* = 6.5 Hz, 1H, C(5)–H of thiouracil), 7.84 (d, *J* = 6.5 Hz, 1H, C(6)–H of thiouracil), 12.54 (s, 1H, NH). ^13^C NMR (DMSO-*d*_6_, 62.5 MHz) *δ*_ppm_ = 13.43, 18.52, 24.96, 30.07, 33.58, 63.82, 109.61, 146.44, 153.70, 171.21, 175.96. Anal. calc. for C_11_H_16_N_2_O_3_S: C, 51.54; H, 6.29; N, 10.93; found: C, 51.68; H, 6.45; N, 11.07.

##### 4.1.5.28 3-(6-Amino-purine-9-yl)-propionic acid butyl ester (3d)^43^

Column chromatography on silica gel eluted with hexane/EtOAc (1 : 3) afforded pure product as white solid (74%); m. p. 135–136 °C. IR (KBr): 3265, 3110, 2932, 1728 cm^−1^. ^1^H NMR (CDCl_3_, 250 MHz) *δ*_ppm_ = 0.92 (t, *J* = 6.7 Hz, 3H, CH_3_), 1.25–1.34 (m, 2H, C*H*_2_CH_3_), 1.53–1.58 (m, 2H, OCH_2_C*H*_2_), 2.96 (t, *J* = 5.8 Hz, 2H, NCH_2_C*H*_2_), 4.09 (t, *J* = 6.7 Hz, 2H, OCH_2_), 4.53 (t, 2H, *J* = 5.8 Hz, NCH_2_), 6.37 (s, 2H, NH_2_), 7.99 (s, 1H, C(2)–H of adenine), 8.42 (s, 1H, C(8)–H of adenine). ^13^C NMR (CDCl_3_, 62.5 MHz) *δ*_ppm_ = 14.32, 19.38, 30.96, 39.85, 46.44, 65.41, 121.67, 138.47, 150.24, 152.78, 156.11, 171.41. Anal. calc. for C_12_H_17_N_5_O_2_: C, 54.74; H, 6.51; N, 26.60; found: C, 54.50; H, 6.63; N, 26.48.

##### 4.1.5.29 3-(6-Amino-purine-9-yl)-2-methyl-propionic acid ethyl ester (3e)^43^

Column chromatography on silica gel eluted with hexane/EtOAc (1 : 3) afforded pure product as white solid (53%); m. p. 136–137 °C. IR (KBr): 3511, 3296, 3139, 2930, 1728 cm^−1^. ^1^H NMR (CDCl_3_, 250 MHz) *δ*_ppm_ = 1.04–1.20 (complex, 6H, CH_2_C*H*_3_, CHC*H*_3_), 3.08 (br s, 1H, C*H*CH_3_), 4.04 (q, *J* = 6.6 Hz, 2H, C*H*_2_CH_3_), 4.20 (dd, *J* = 8.3, 13.9 Hz, 1H, NCH_2_), 4.38 (dd, *J* = 8.6, 13.9 Hz, 1H, NCH_2_), 6.19 (s, 2H, NH_2_), 7.77 (s, 1H, C(2)–H of adenine), 8.28 (s, 1H, C(8)–H of adenine). ^13^C NMR (CDCl_3_, 62.5 MHz) *δ*_ppm_ = 14.37, 15.49, 39.05, 46.35, 61.42, 119.76, 141.45, 150.34, 153.22, 156.17, 174.45. Anal. calc. for C_11_H_15_N_5_O_2_: C, 53.00; H, 6.07; N, 28.10; found: C, 53.18; H, 6.21; N, 27.96.

##### 4.1.5.30 Butyl 3-(1*H*-benzo[*d*]imidazol-1-yl)propanoate (3f)^41^

Column chromatography on silica gel eluted with hexane/EtOAc (2 : 1) afforded pure product as white solid (89%); m. p. 114–115 °C. IR (KBr): 3088, 2960, 1735, 1493, 1245 cm^−1^. ^1^H NMR (CDCl_3_, 250 MHz) *δ*_ppm_ = 0.73 (t, *J* = 7.3 Hz, 3H, CH_3_), 1.09–1.23 (m, 2H, CH_3_C*H*_2_), 1.35–1.47 (m, 2H, OCH_2_C*H*_2_), 2.69 (t, *J* = 6.7 Hz, 2H, O=CCH_2_), 3.90 (t, *J* = 6.7 Hz, 2H, NCH_2_), 4.30 (t, *J* = 6.5 Hz, 2H, OCH_2_), 7.13–7.29 (m, 2H, aryl), 7.67–7.71 (m, 2H, aryl), 7.84 (s, 1H, C(2)–H of benzimidazole). ^13^C NMR (CDCl_3_, 62.5 MHz) *δ*_ppm_ = 13.55, 18.91, 30.36, 34.30, 40.26, 64.93, 109.32, 120.33, 122.11, 122.92, 133.32, 143.31, 143.73, 170.64. Anal. calc. for C_14_H_18_N_2_O_2_: C, 68.27; H, 7.37; N, 11.37; found: C, 68.42; H, 7.48; N, 11.26.

## Author contributions

Mohammad Navid Soltani Rad: writing – review & editing, supervision, project administration, funding acquisition, conceptualization. Somayeh Behrouz: writing – review & editing, project administration, funding acquisition, data curation, conceptualization. Hamid Reza Mohammadnia Afroozi: methodology, investigation.

## Conflicts of interest

The authors declare that they have no known competing financial interests or personal relationships that could have appeared to influence the work reported in this paper.

## Supplementary Material

RA-016-D5RA10042A-s001

## Data Availability

Data will be made available on request. Supplementary information (SI) is available. See DOI: https://doi.org/10.1039/d5ra10042a.
