# Supplementary material for: Benzyl methyl morpholinium hydroxide (BMMorph)OH: a new basic ionic liquid for N-alkylation of bioactive N-heterocycles
Source: RSC Adv. 2026 Feb 9;16(9):7923–35. doi: 10.1039/d5ra10042a (PMC12885059; doi:10.1039/d5ra10042a)

Supplementary Information (SI) for RSC Advances.
This journal is © The Royal Society of Chemistry 2026

RSC Advances

## (Electronic Supplementary Information)

# Benzyl Methyl Morpholinium Hydroxide (BMMorph)OH: A New Basic Ionic Liquid for *N*-Alkylation of Bioactive *N*-Heterocycles

**Mohammad Navid Soltani Rad, [a,b]* Somayeh Behrouz, [a,b]* Hamid Reza Mohammadnia Afroozi [a]**

[a]) *Department of Chemistry, Shiraz University of Technology, Shiraz 71555-313, Iran*

[b]) *Medicinal Chemistry Research Laboratory, Novel Technology for Health Research Center, Shiraz University of Technology, Shiraz 71555-313, Iran*

Tel.: +98 71 3735 4500; Fax: +98 71 3735 4520

E-mail address: soltani@sutech.ac.ir (M.N. Soltani Rad); behrouz@sutech.ac.ir (S. Behrouz)

# Table of Content


| **Content** | **Page** |
|---|---|
| $^{1}$HNMR and $^{13}$CNMR of (BMMorph)OH | 3 |
| $^{1}$HNMR and $^{13}$CNMR of **1a** | 4 |
| $^{1}$HNMR and $^{13}$CNMR of **1b** | 5 |
| $^{1}$HNMR and $^{13}$CNMR of **1c** | 6 |
| $^{1}$HNMR and $^{13}$CNMR of **1d** | 7 |
| $^{1}$HNMR and $^{13}$CNMR of **1e** | 8 |
| $^{1}$HNMR and $^{13}$CNMR of **1f** | 9 |
| $^{1}$HNMR and $^{13}$CNMR of **1g** | 10 |
| $^{1}$HNMR and $^{13}$CNMR of **1h** | 11 |
| $^{1}$HNMR and $^{13}$CNMR of **1i** | 12 |
| $^{1}$HNMR and $^{13}$CNMR of **1j** | 13 |
| $^{1}$HNMR and $^{13}$CNMR of **1k** | 14 |
| $^{1}$HNMR and $^{13}$CNMR of **1l** | 15 |
| $^{1}$HNMR and $^{13}$CNMR of **1m** | 16 |
| $^{1}$HNMR and $^{13}$CNMR of **1n** | 17 |
| $^{1}$HNMR and $^{13}$CNMR of **1o** | 18 |
| $^{1}$HNMR and $^{13}$CNMR of **1p** | 19 |
| $^{1}$HNMR and $^{13}$CNMR of **1q** | 20 |
| $^{1}$HNMR and $^{13}$CNMR of **1r** | 21 |
| $^{1}$HNMR and $^{13}$CNMR of **2a** | 22 |
| $^{1}$HNMR and $^{13}$CNMR of **2b** | 23 |
| $^{1}$HNMR and $^{13}$CNMR of **2c** | 24 |
| $^{1}$HNMR and $^{13}$CNMR of **2d** | 25 |
| $^{1}$HNMR and $^{13}$CNMR of **2e** | 26 |
| $^{1}$HNMR and $^{13}$CNMR of **2f** | 27 |
| $^{1}$HNMR and $^{13}$CNMR of **3a** | 28 |
| $^{1}$HNMR and $^{13}$CNMR of **3b** | 29 |
| $^{1}$HNMR and $^{13}$CNMR of **3c** | 30 |
| $^{1}$HNMR and $^{13}$CNMR of **3d** | 31 |
| $^{1}$HNMR and $^{13}$CNMR of **3e** | 32 |
| $^{1}$HNMR and $^{13}$CNMR of **3f** | 33 |

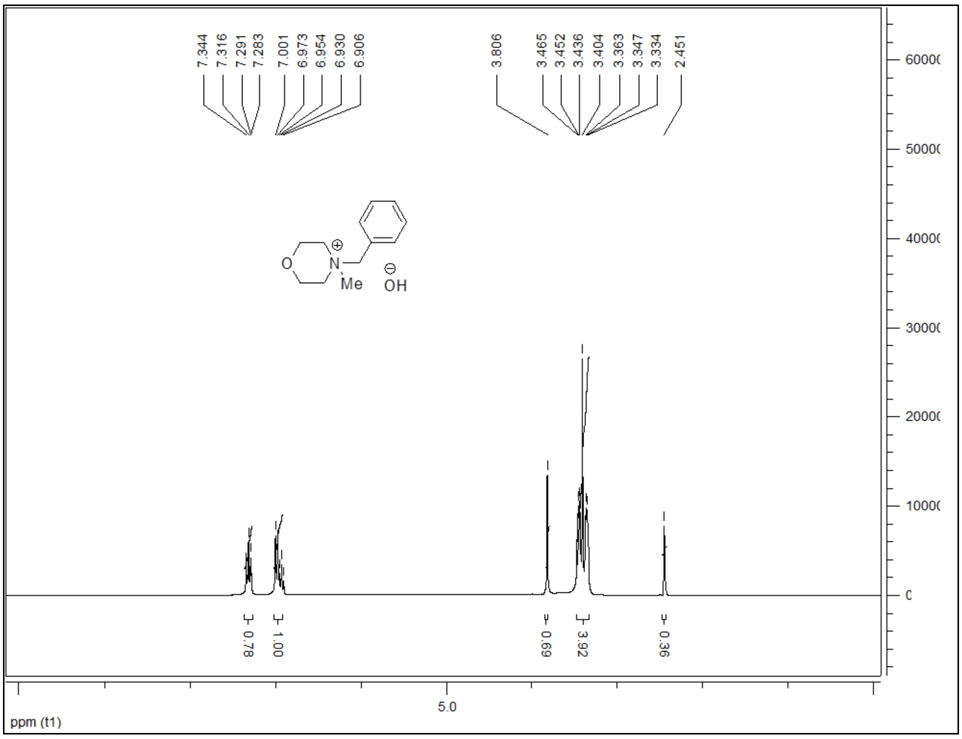
7.344
7.316
7.291
7.283
7.001
6.973
6.954
6.930
6.906
3.806
3.465
3.452
3.436
3.404
3.363
3.347
3.334
2.451
O
N
Me
OH
0.78
1.00
0.69
3.92
0.36
5.0
ppm (t1)

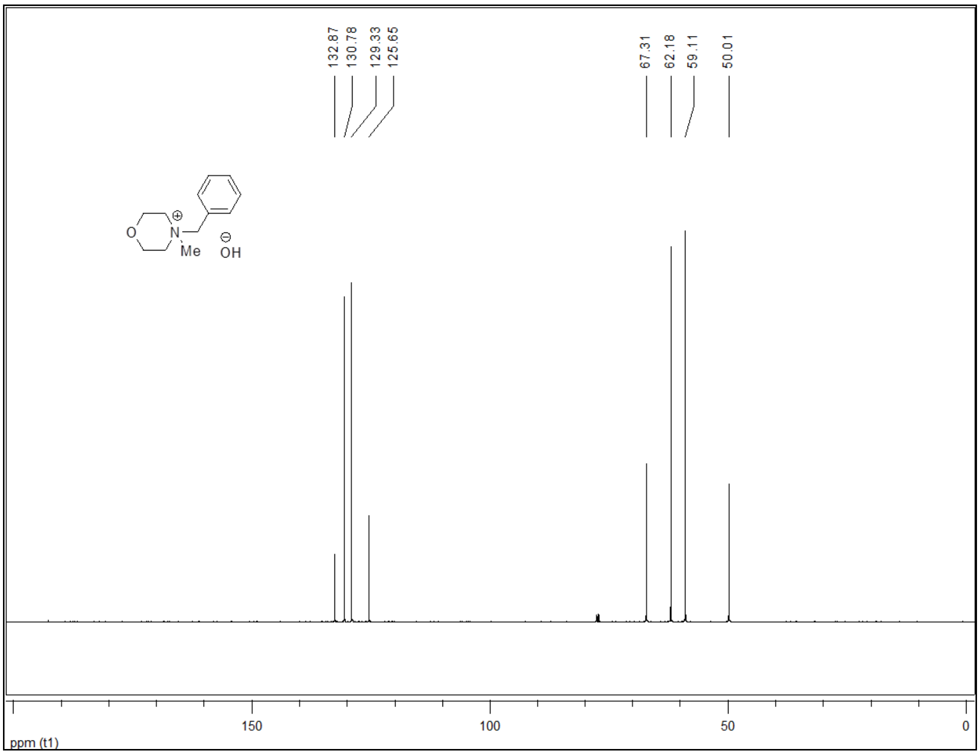
132.87
130.78
129.33
125.65
67.31
62.18
59.11
50.01
O
N
Me
OH
150
100
50
0
ppm (t1)

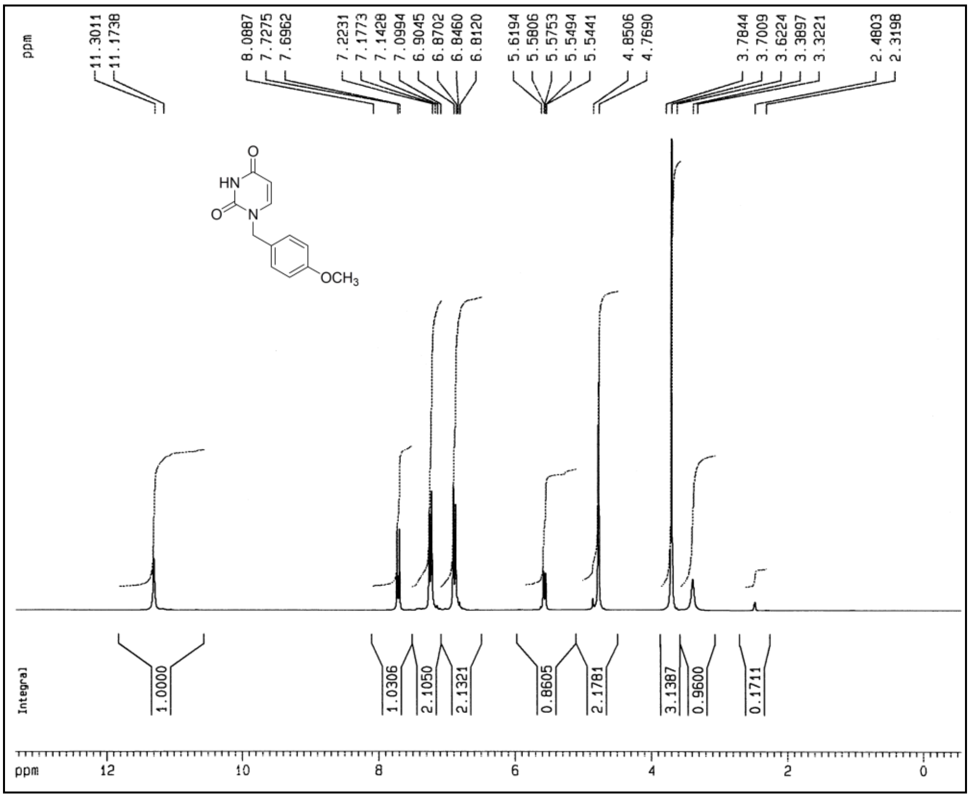
ppm
11.3011
11.1738
8.0887
7.7275
7.6962
7.2231
7.1773
7.1428
7.0994
6.9045
6.8702
6.8460
6.8120
5.6194
5.5806
5.5753
5.5494
5.5441
4.8506
4.7690
3.7844
3.7009
3.6224
3.3897
3.3221
2.4803
2.3198
HN
O
O
N
OCH3
Integral
1.0000
1.0306
2.1050
2.1321
0.8605
2.1781
3.1387
0.9600
0.1711
ppm
12
10
8
6
4
2
0

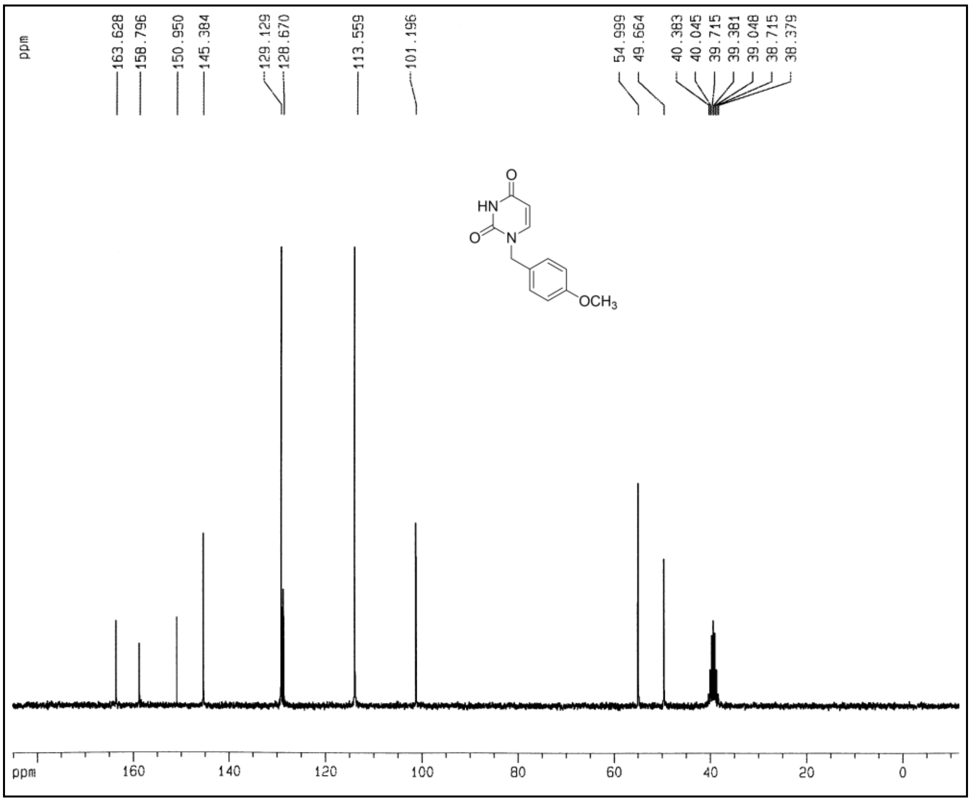
ppm
163.628
158.796
150.950
145.384
129.129
128.670
113.559
101.196
54.999
49.664
40.383
40.045
39.715
39.381
39.048
38.715
38.379
HN
O
O
N
OCH3
ppm
160
140
120
100
80
60
40
20
0

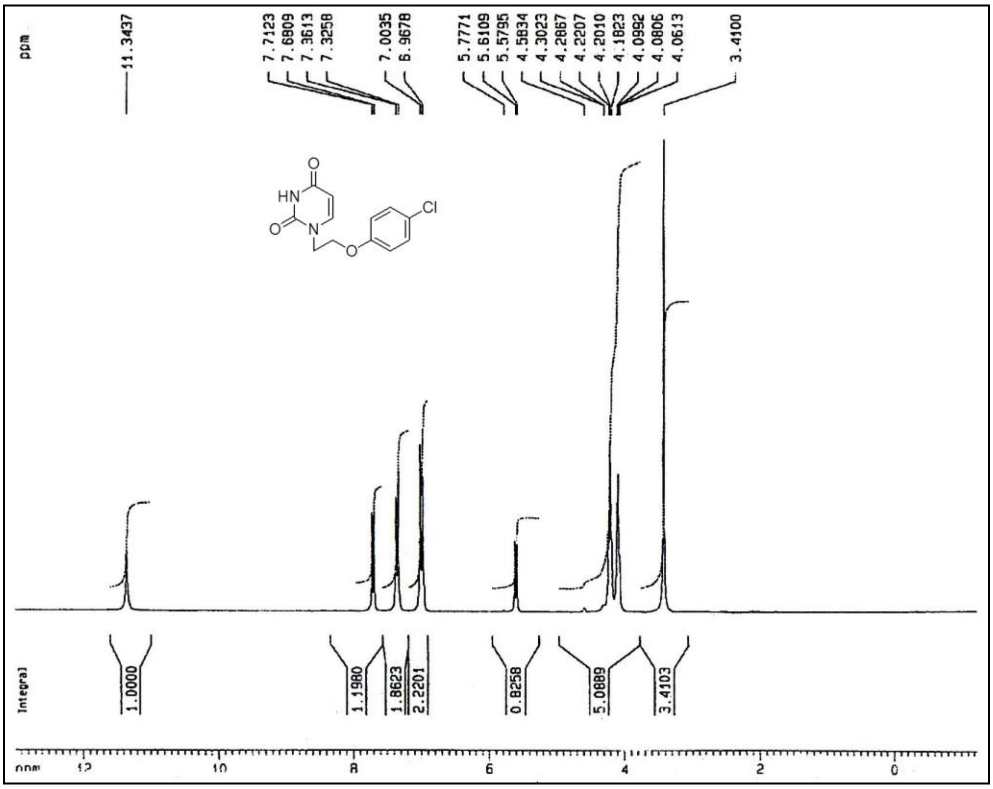
ppm
11.3437
7.7123
7.6809
7.3613
7.3258
7.0035
6.9678
5.7771
5.6109
5.5795
4.5834
4.3023
4.2867
4.2207
4.2010
4.1823
4.0982
4.0806
4.0613
3.4100
HN
O
N
O
Cl
Integral
1.0000
1.1980
1.8623
2.2201
0.8258
5.0889
3.4103
ppm
12
10
8
6
4
2
0

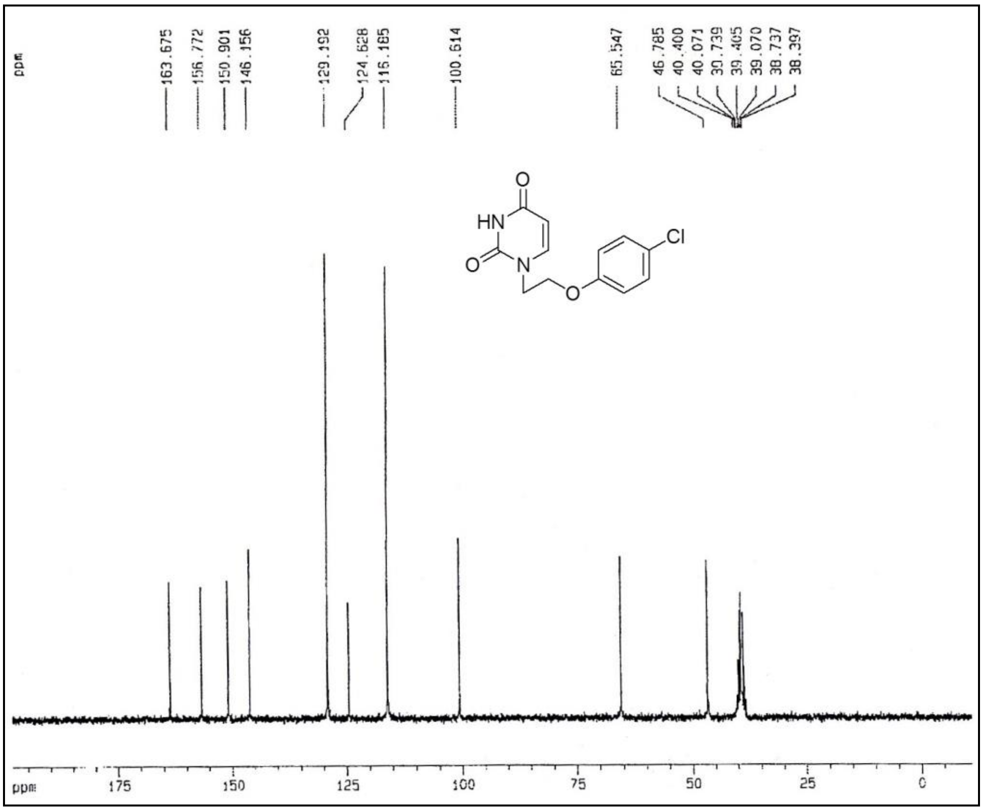
ppm
163.675
156.772
150.901
146.156
129.192
124.628
116.165
100.614
65.547
46.785
40.400
40.071
39.739
39.405
39.070
38.737
38.397
HN
O
N
O
Cl
ppm
175
150
125
100
75
50
25
0

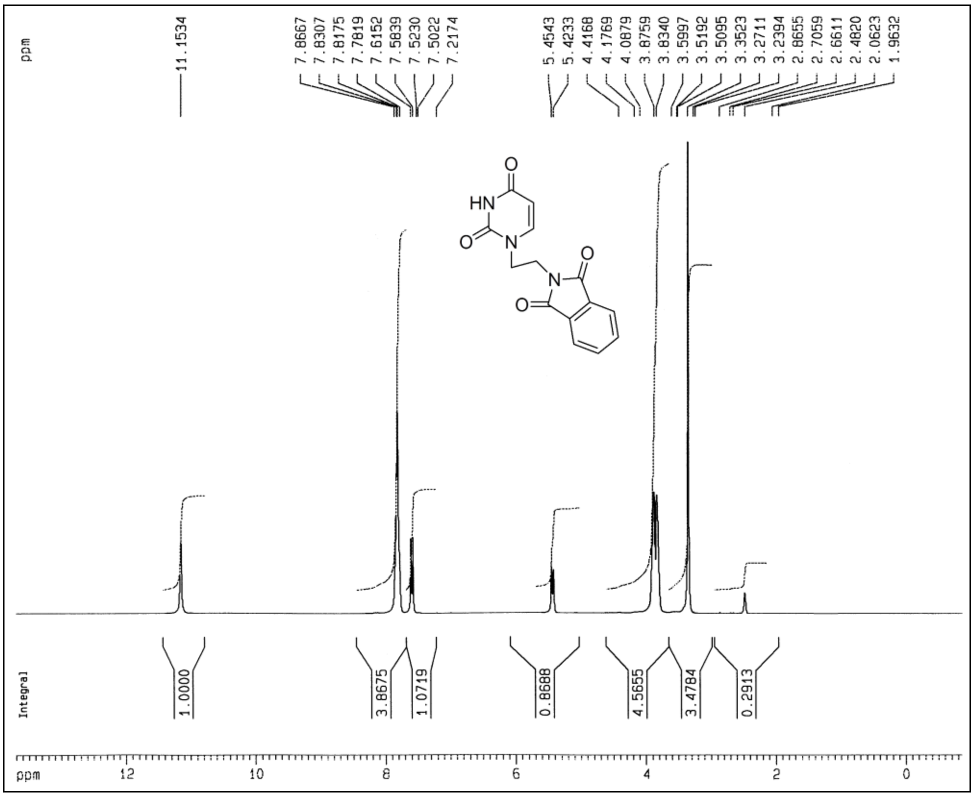
ppm
11.1534
7.8667
7.8307
7.8175
7.7819
7.6152
7.5839
7.5230
7.5022
7.2174
5.4543
5.4233
4.4168
4.1769
4.0879
3.8759
3.8340
3.5997
3.5192
3.5095
3.3523
3.2711
3.2394
2.8655
2.7059
2.6611
2.4820
2.0623
1.9632
HN
O
N
O
O
N
O
Integral
1.0000
3.8675
1.0719
0.8688
4.5655
3.4784
0.2913
ppm
12
10
8
6
4
2
0

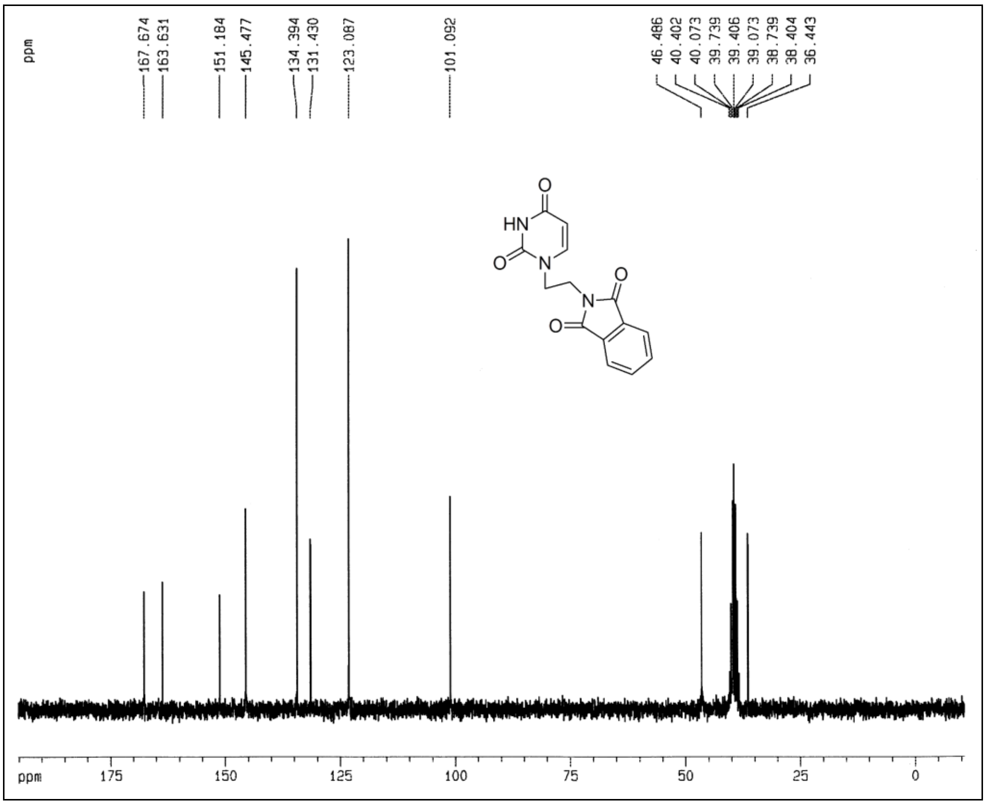
ppm
167.674
163.631
151.184
145.477
134.394
131.430
123.087
101.092
46.486
40.402
40.073
39.739
39.406
39.073
38.739
38.404
36.443
HN
O
N
O
O
N
O
ppm
175
150
125
100
75
50
25
0

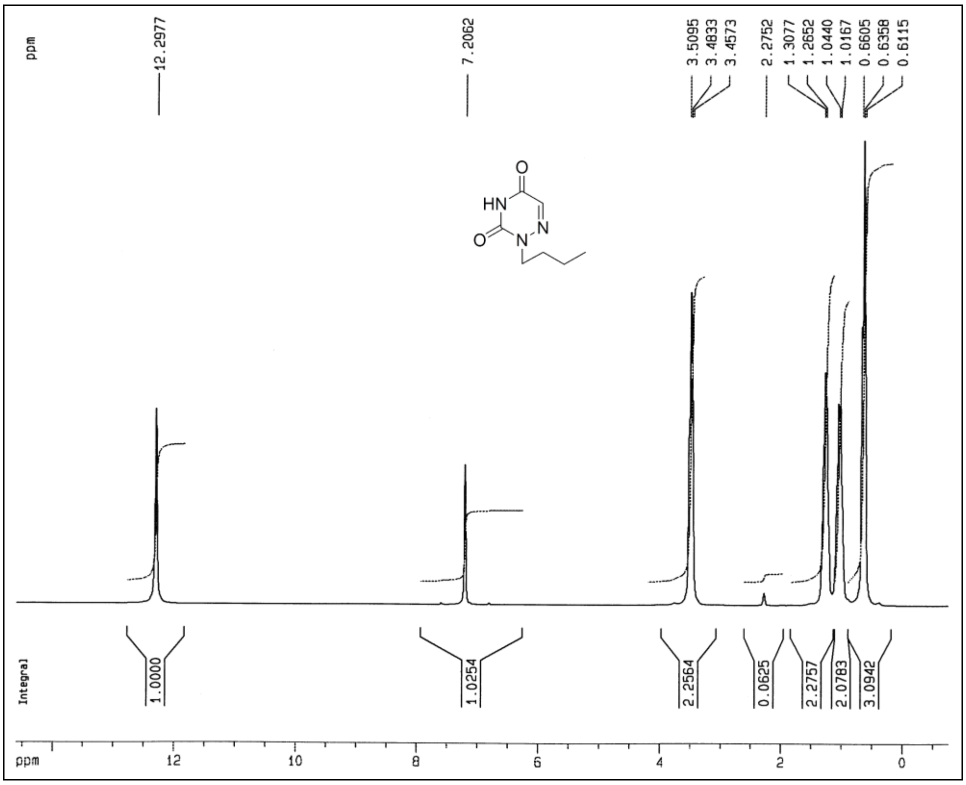
ppm
12.2977
7.2062
3.5095
3.4833
3.4573
2.2752
1.3077
1.2652
1.0440
1.0167
0.6605
0.6358
0.6115
HN
O
N
N
O
Integral
1.0000
1.0254
2.2564
0.0625
2.2757
2.0783
3.0942
ppm
12
10
8
6
4
2
0

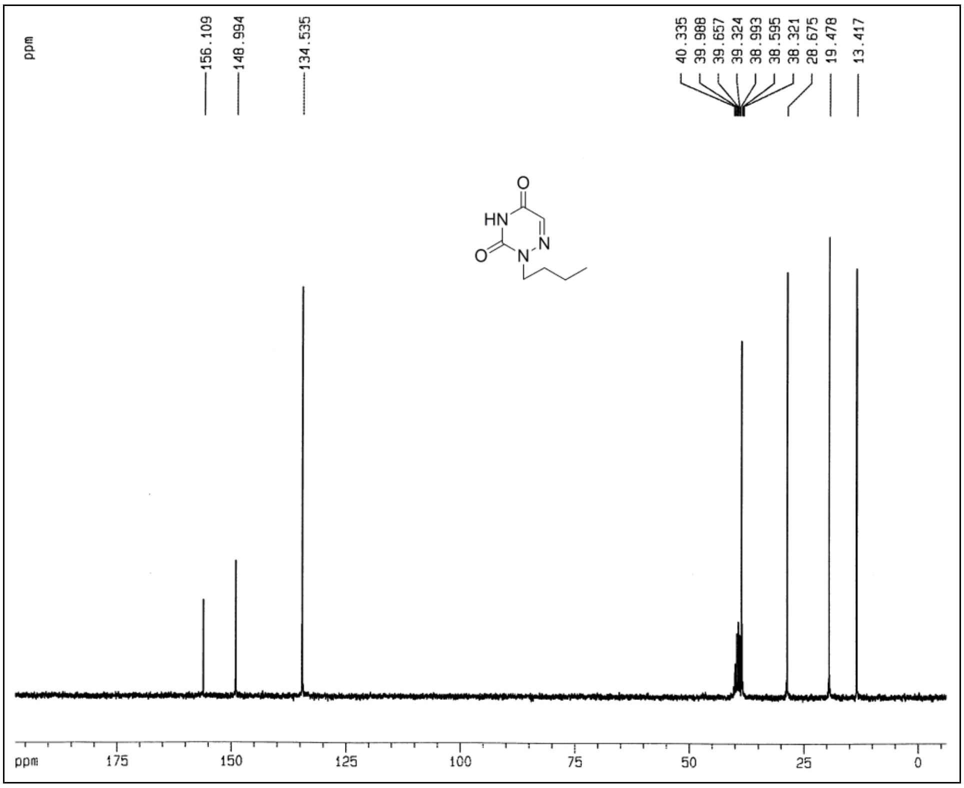
ppm
156.109
148.994
134.535
40.335
39.988
39.657
39.324
38.993
38.595
38.321
28.675
19.478
13.417
HN
O
N
N
O
ppm
175
150
125
100
75
50
25
0

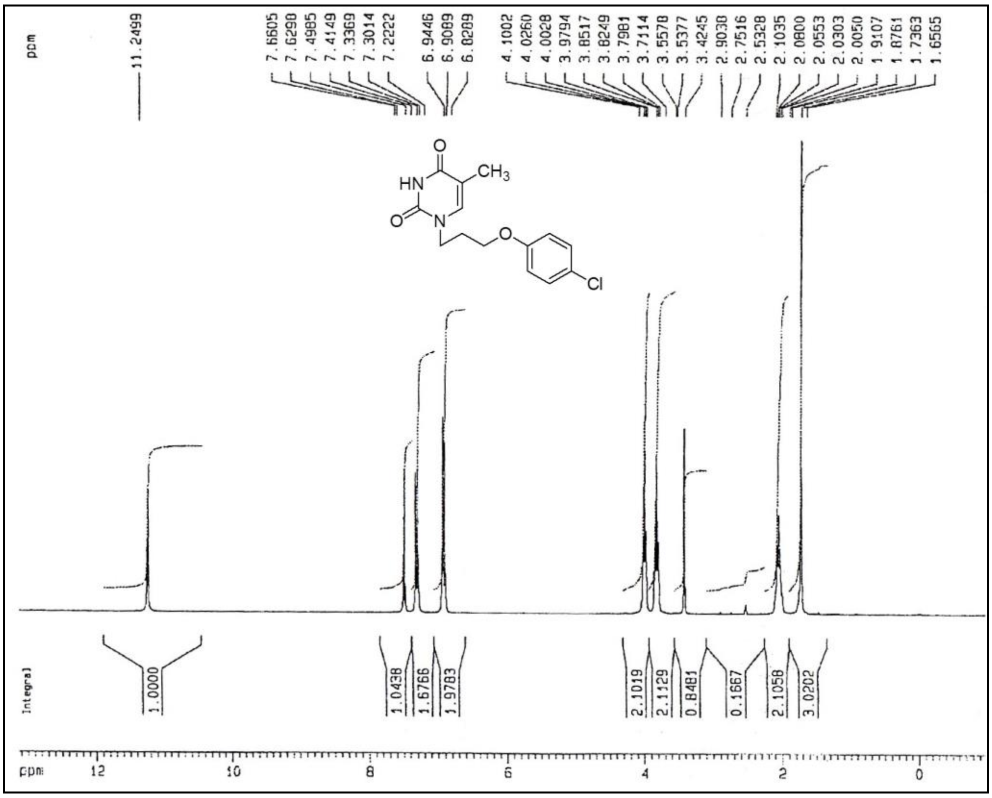
ppm
11.2499
7.6605
7.6290
7.4985
7.4149
7.3369
7.3014
7.2222
6.9446
6.9089
6.8209
4.1002
4.0260
4.0028
3.9794
3.8517
3.8249
3.7981
3.7114
3.5578
3.5377
3.4245
2.9038
2.7516
2.5328
2.1035
2.0800
2.0553
2.0303
2.0050
1.9107
1.8761
1.7363
1.6565
HN
O
O
N
CH3
O
Cl
Integral
1.0000
1.0438
1.6766
1.9783
2.1019
2.1129
0.8481
0.1667
2.1058
3.0202
ppm
12
10
8
6
4
2
0

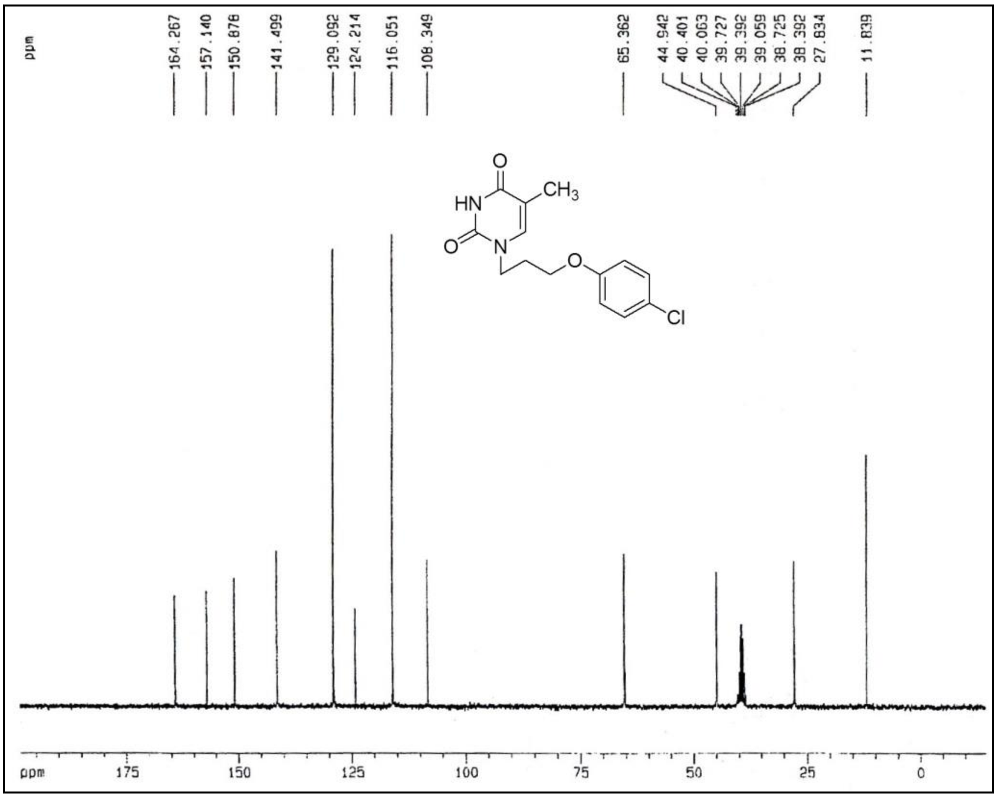
ppm
164.267
157.140
150.878
141.499
129.092
124.214
116.051
108.349
65.362
44.942
40.401
40.063
39.727
39.392
39.059
38.725
38.392
27.834
11.839
HN
O
O
N
CH3
O
Cl
ppm
175
150
125
100
75
50
25
0

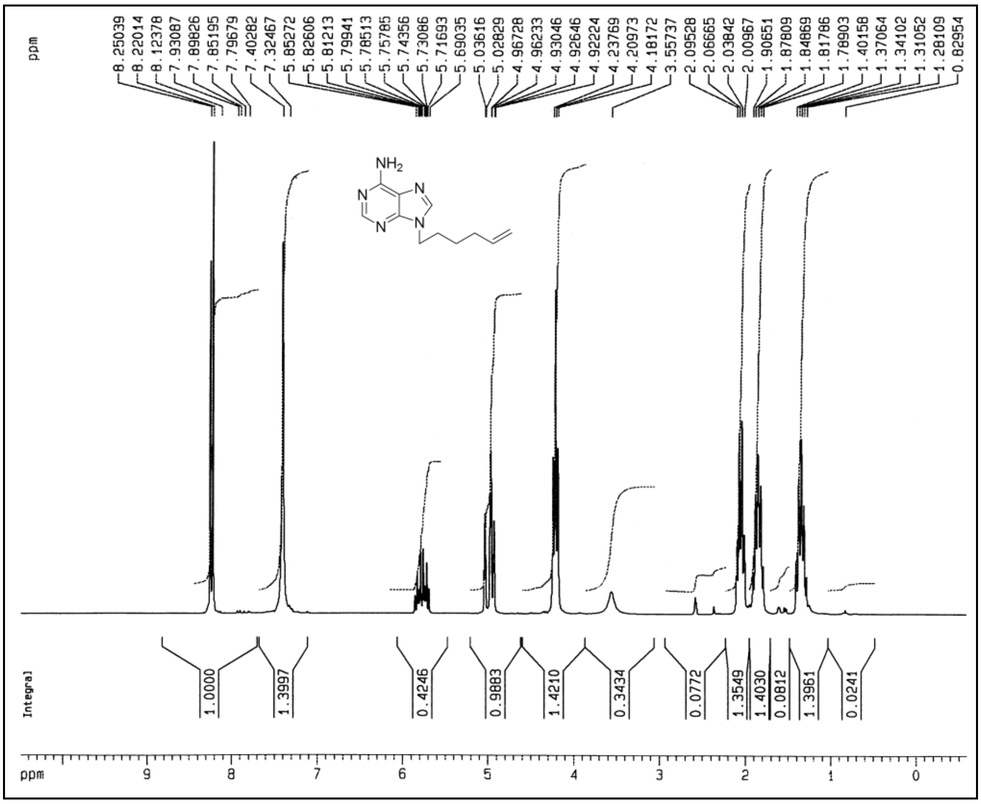
ppm
8.25039
8.22014
8.12378
7.93087
7.89826
7.85195
7.79679
7.40282
7.32467
5.85272
5.82606
5.81213
5.79941
5.78513
5.75785
5.74356
5.73086
5.71693
5.69035
5.03616
5.02829
4.96728
4.96233
4.93046
4.92646
4.92224
4.23769
4.20973
4.18172
3.55737
2.09528
2.06665
2.03842
2.00967
1.90651
1.87809
1.84869
1.81786
1.78903
1.40158
1.37064
1.34102
1.31052
1.28109
0.82954
$NH_2$
N
N
N
N
Integral
1.0000
1.3997
0.4246
0.9883
1.4210
0.3434
0.0772
1.3549
1.4030
0.0812
1.3961
0.0241
ppm 9 8 7 6 5 4 3 2 1 0

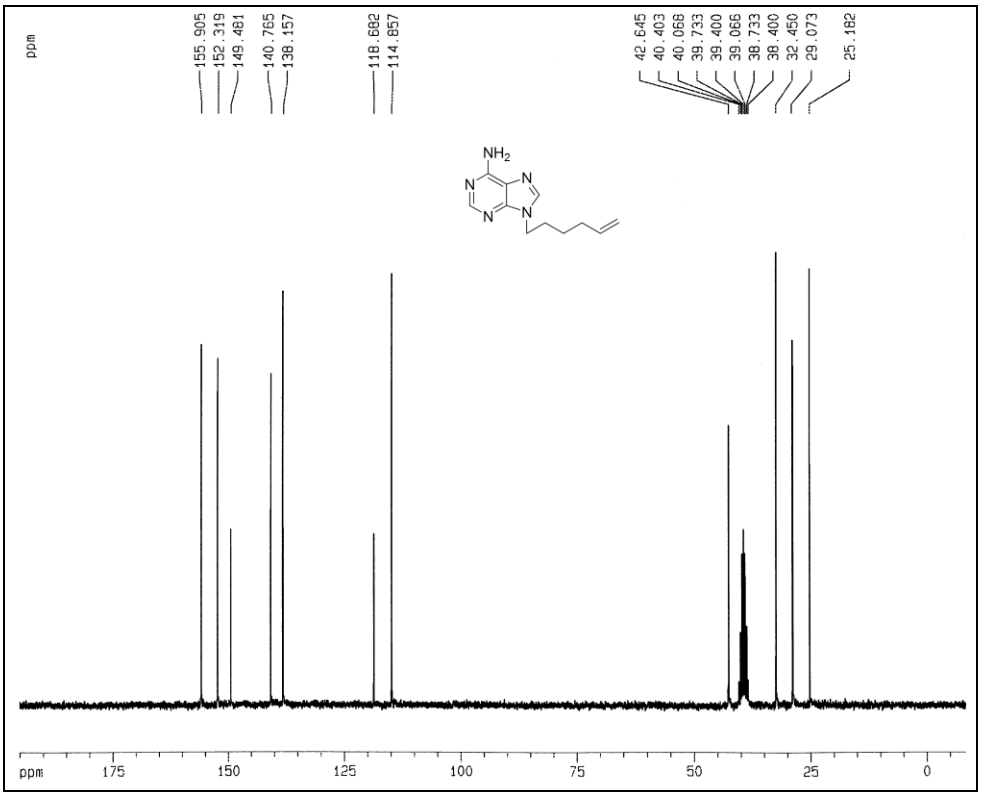
ppm
155.905
152.319
149.481
140.765
138.157
118.682
114.857
42.645
40.403
40.068
39.733
39.400
39.066
38.733
38.400
32.450
29.073
25.182
$NH_2$
N
N
N
N
ppm 175 150 125 100 75 50 25 0

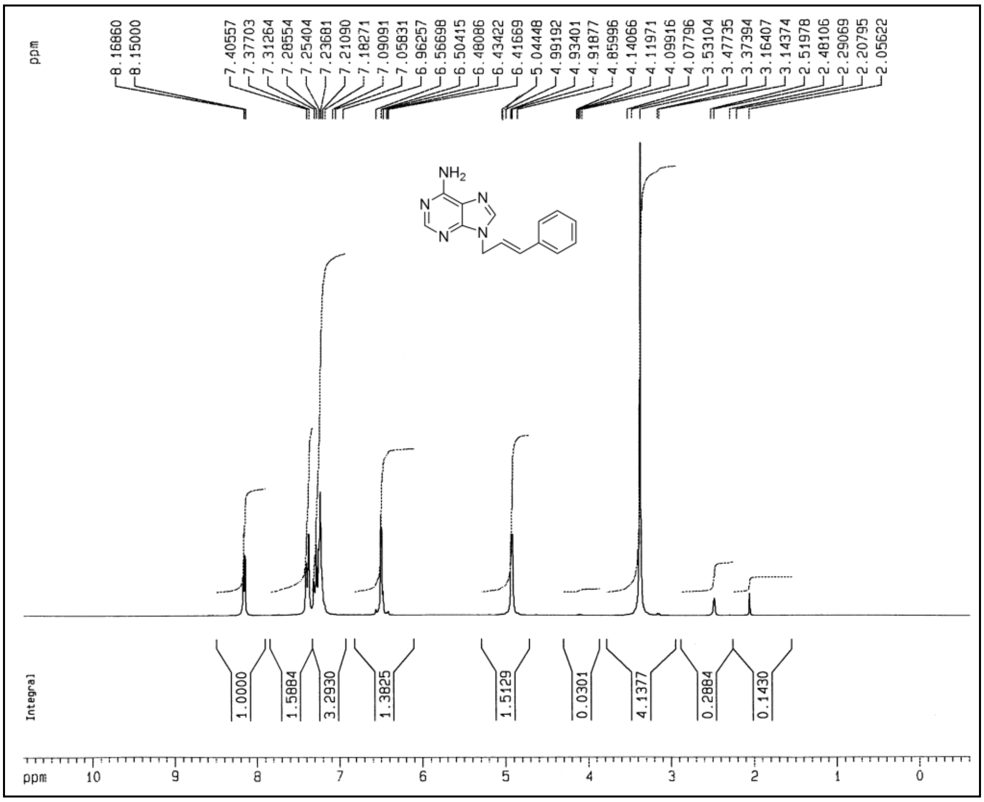

ppm
8.16860
8.15000
7.40557
7.37703
7.31264
7.28554
7.25404
7.23681
7.21090
7.18271
7.09091
7.05831
6.96257
6.56698
6.50415
6.48086
6.43422
6.41669
5.04448
4.99192
4.93401
4.91877
4.85996
4.14066
4.11971
4.09916
4.07796
3.53104
3.47735
3.37394
3.16407
3.14374
2.51978
2.48106
2.29069
2.20795
2.05622
NH2
N
Integral
1.0000
1.5884
3.2930
1.3825
1.5129
0.0301
4.1377
0.2884
0.1430
ppm 10 9 8 7 6 5 4 3 2 1 0


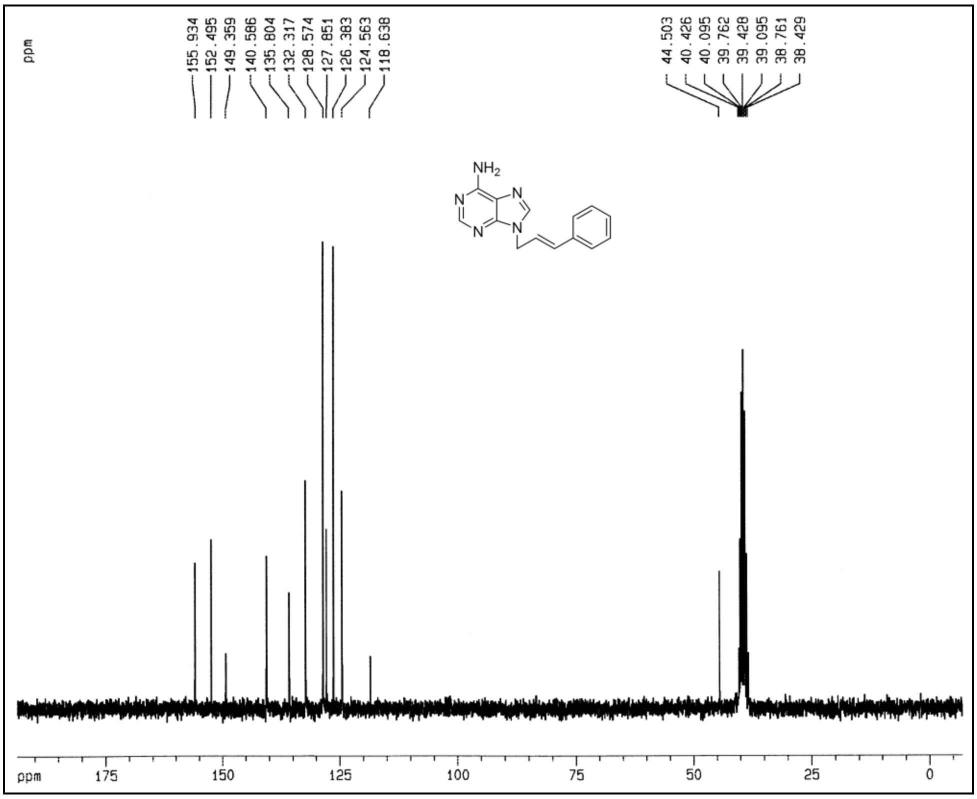

ppm
155.934
152.495
149.359
140.586
135.804
132.317
128.574
127.851
126.383
124.563
118.638
44.503
40.426
40.095
39.762
39.428
39.095
38.761
38.429
NH2
N
ppm 175 150 125 100 75 50 25 0

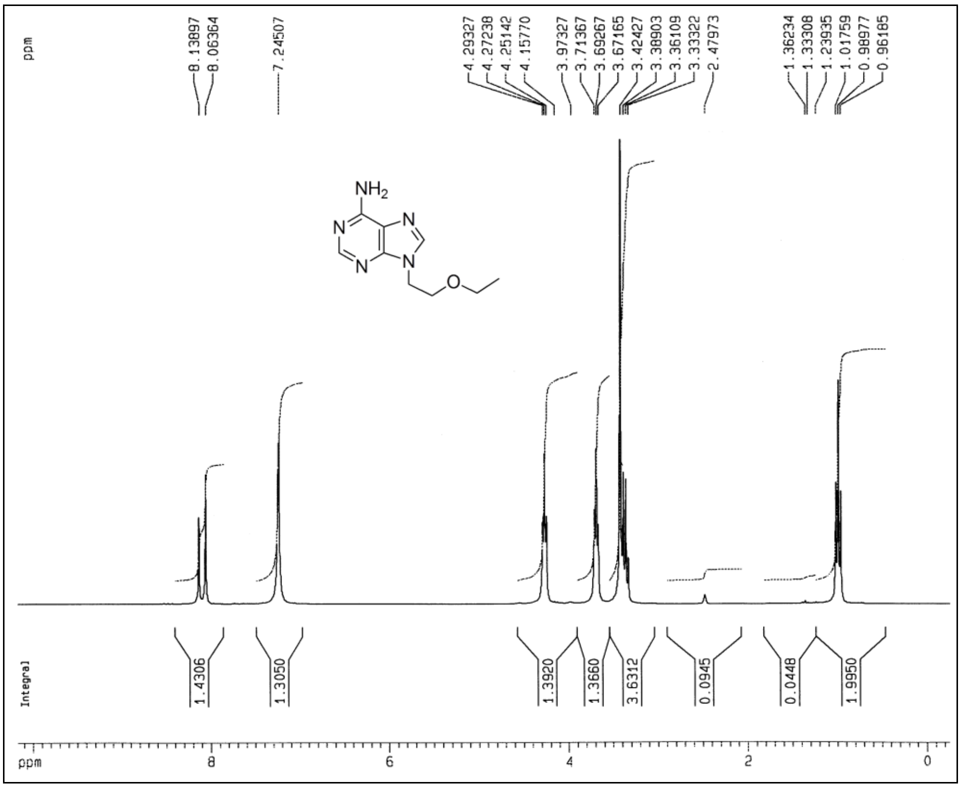
ppm
8.13897
8.06364
7.24507
4.29327
4.27238
4.25142
4.15770
3.97327
3.71367
3.69267
3.67165
3.42427
3.38903
3.36109
3.33322
2.47973
1.36234
1.33308
1.23935
1.01759
0.98977
0.96185
NH2
N
N
N
N
O
Integral
1.4306
1.3050
1.3920
1.3660
3.6312
0.0945
0.0448
1.9950
ppm
8
6
4
2
0

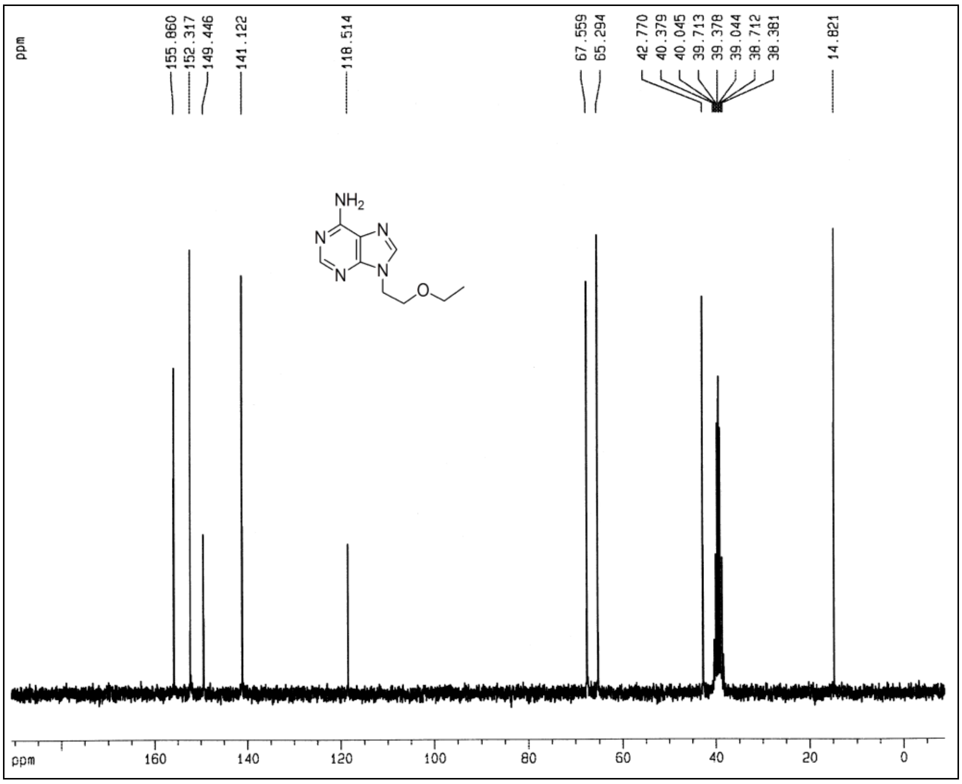
ppm
155.860
152.317
149.446
141.122
118.514
67.559
65.294
42.770
40.379
40.045
39.713
39.378
39.044
38.712
38.381
14.821
NH2
N
N
N
N
O
ppm
160
140
120
100
80
60
40
20
0

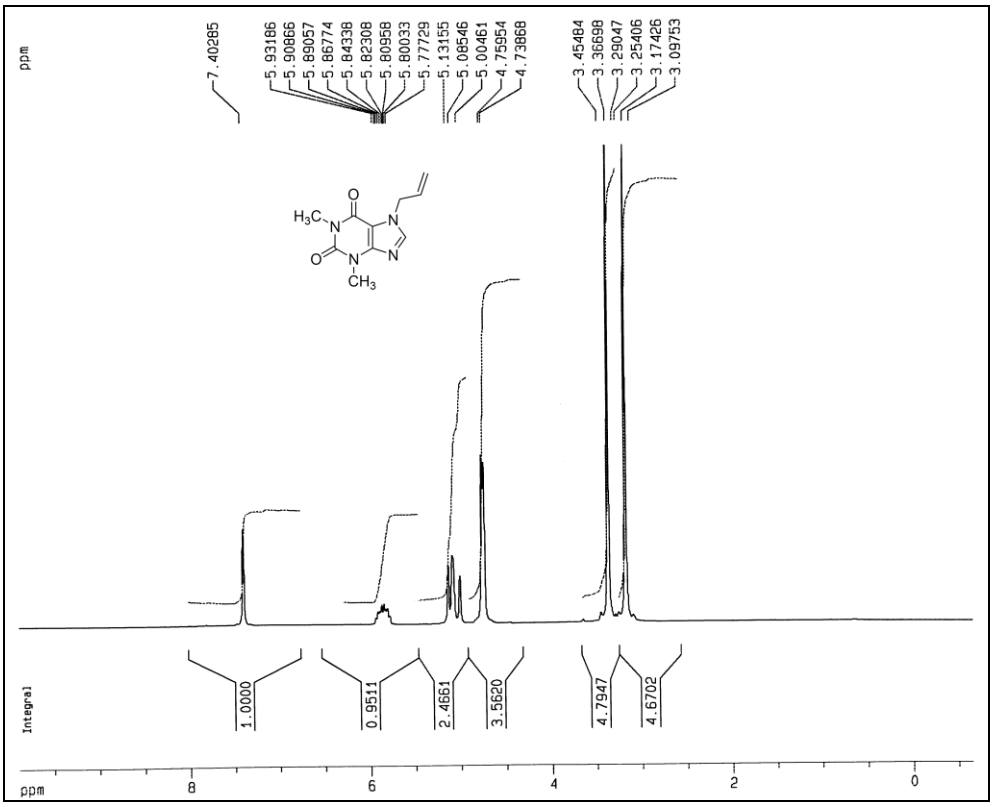
ppm
7.40285
5.93186
5.90866
5.89057
5.86774
5.84338
5.82308
5.80958
5.80033
5.77729
5.13155
5.08546
5.00461
4.75954
4.73868
3.45484
3.36698
3.29047
3.25406
3.17426
3.09753
$H_3C$
O
N
N
N
O
N
$CH_3$
Integral
1.0000
0.9511
2.4661
3.5620
4.7947
4.6702
ppm
8
6
4
2
0

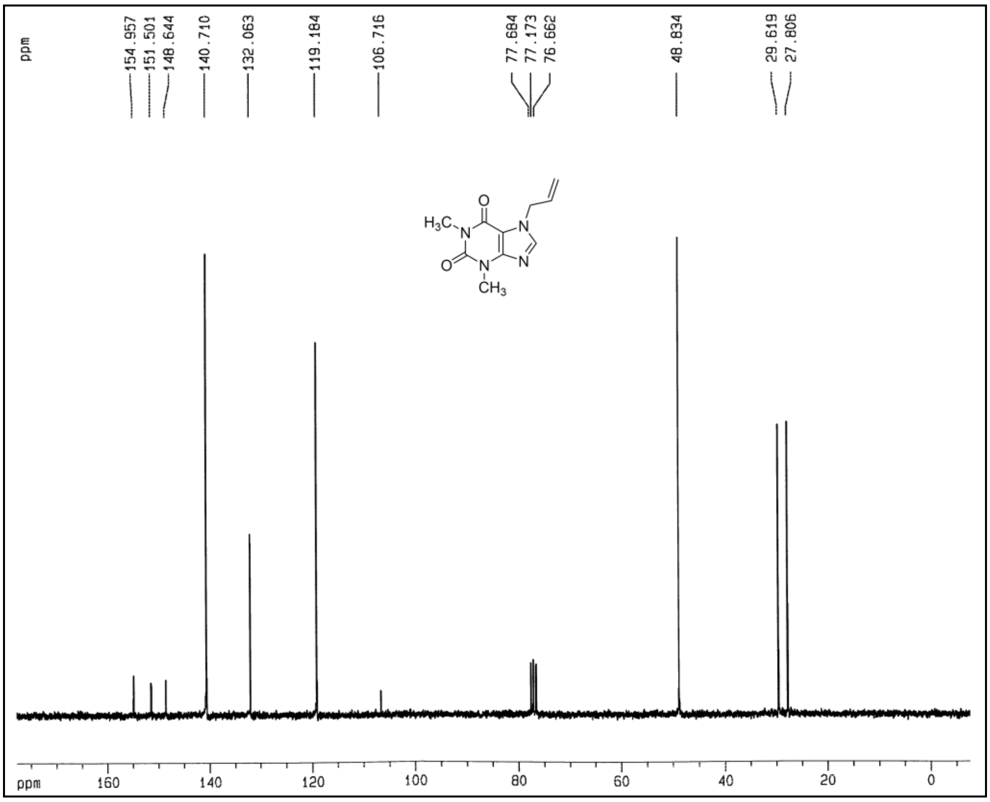
ppm
154.957
151.501
148.644
140.710
132.063
119.184
106.716
77.684
77.173
76.662
48.834
29.619
27.806
$H_3C$
O
N
N
N
O
N
$CH_3$
ppm
160
140
120
100
80
60
40
20
0

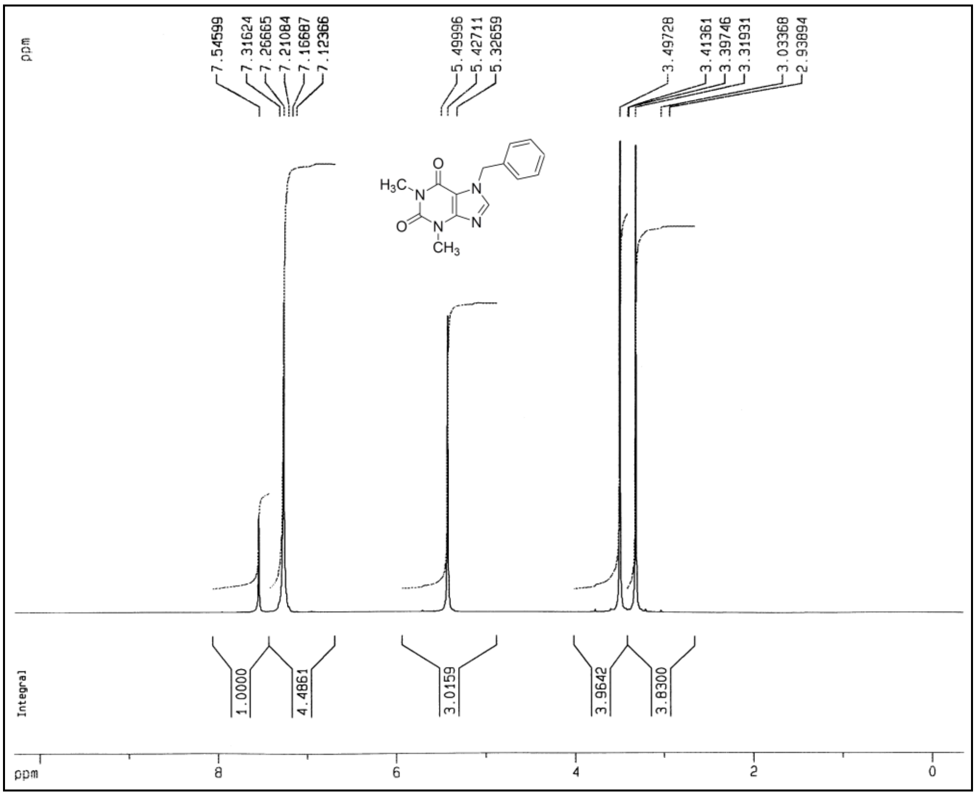
ppm
7.54599
7.31624
7.26665
7.21084
7.16687
7.12366
5.49996
5.42711
5.32659
3.49728
3.41361
3.39746
3.31931
3.03368
2.93894
Integral
1.0000
4.4861
3.0159
3.9642
3.8300
ppm
8
6
4
2
0

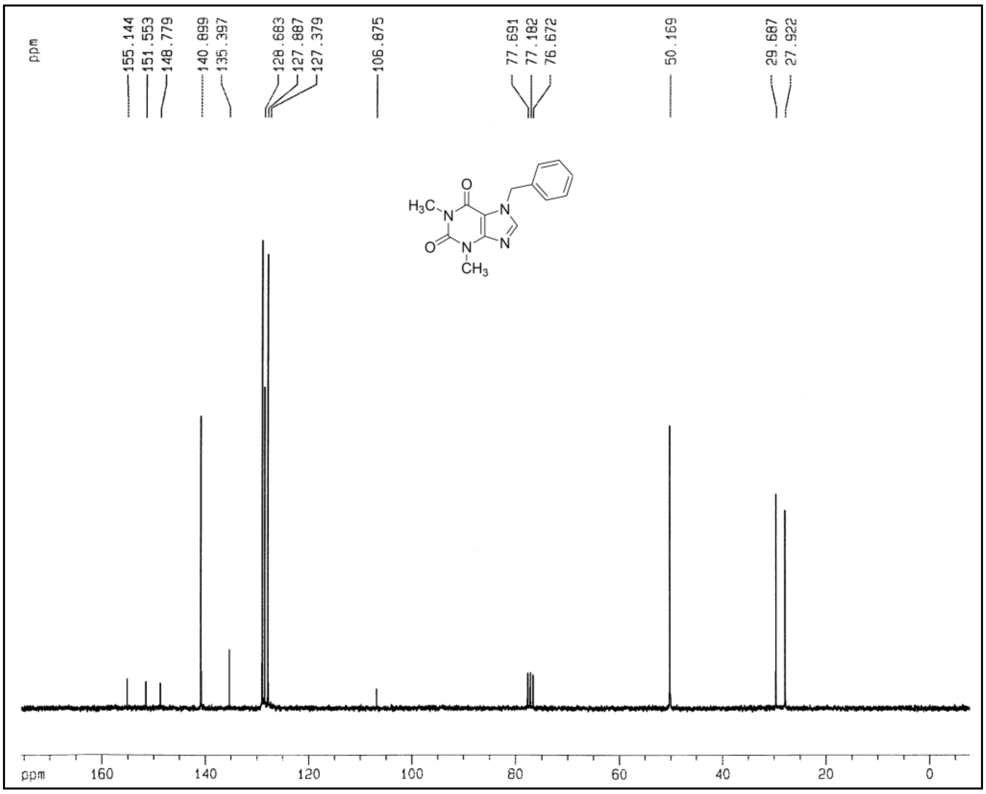
ppm
155.144
151.553
148.779
140.899
135.397
128.683
127.887
127.379
106.875
77.691
77.182
76.672
50.169
29.687
27.922
ppm
160
140
120
100
80
60
40
20
0

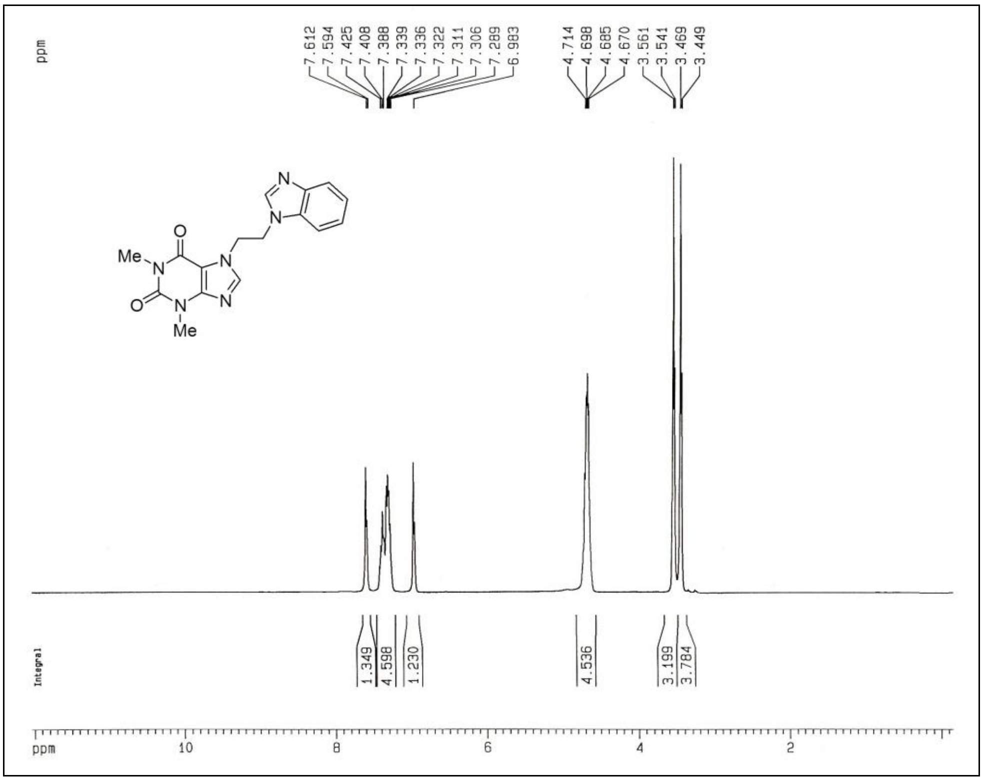

7.612
7.594
7.425
7.408
7.388
7.339
7.336
7.322
7.311
7.306
7.289
6.983
4.714
4.698
4.685
4.670
3.561
3.541
3.469
3.449
1.349
4.598
1.230
4.536
3.199
3.784


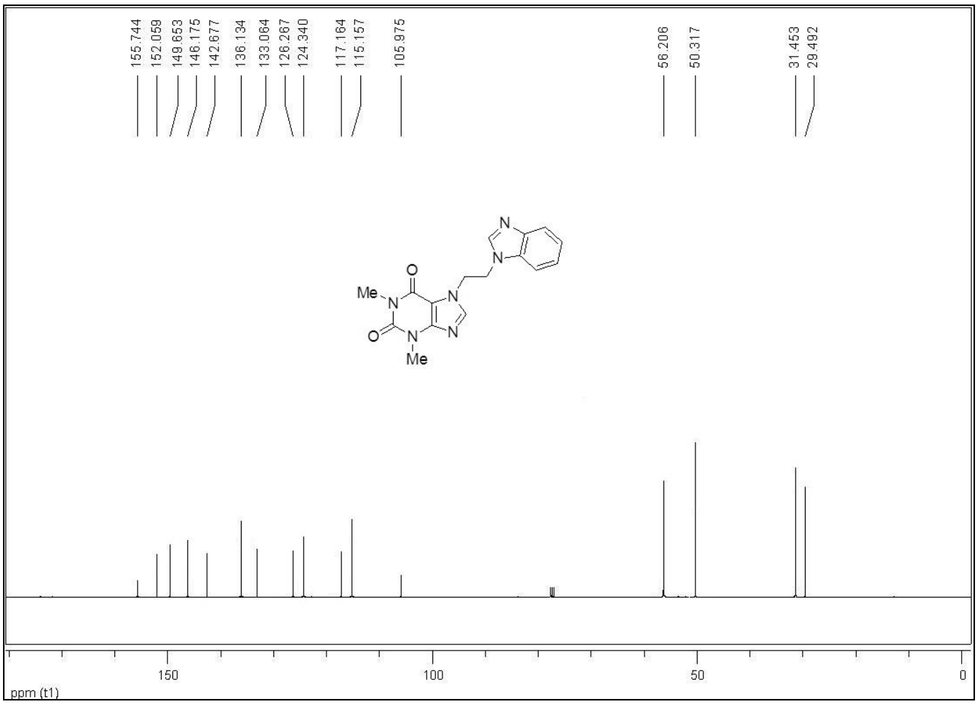

155.744
152.059
149.653
146.175
142.677
136.134
133.064
126.267
124.340
117.164
115.157
105.975
56.206
50.317
31.453
29.492

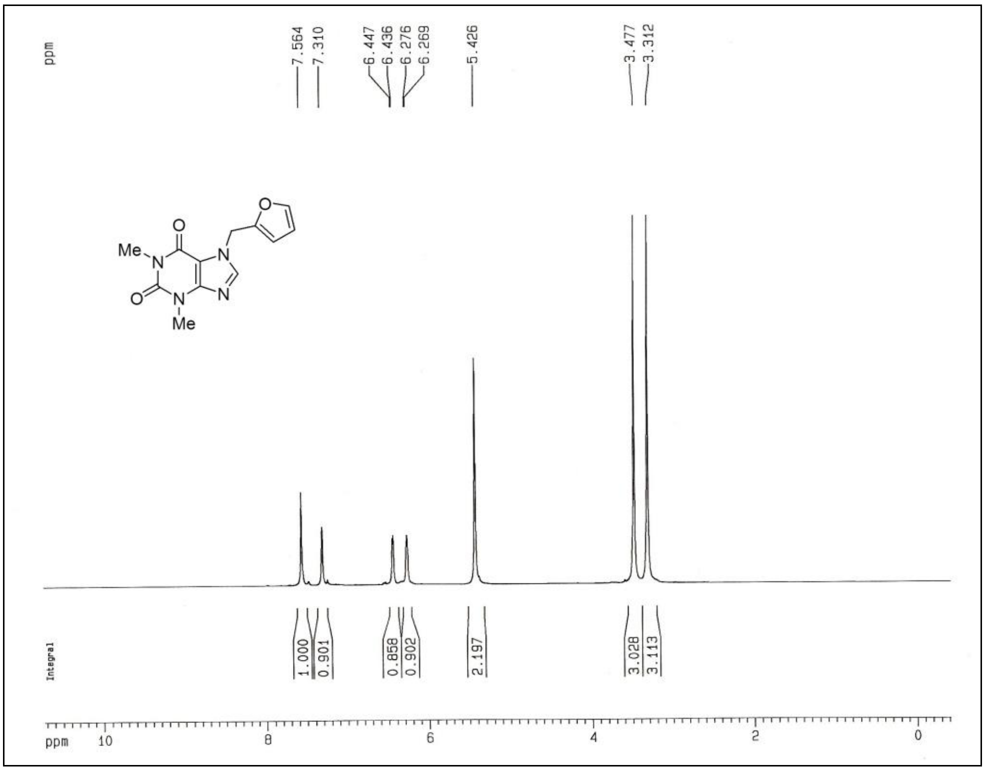
ppm
7.564
7.310
6.447
6.436
6.276
6.269
5.426
3.477
3.312
Me
N
O
N
N
N
O
Me
O
Integral
1.000
0.901
0.858
0.902
2.197
3.028
3.113
ppm
10
8
6
4
2
0

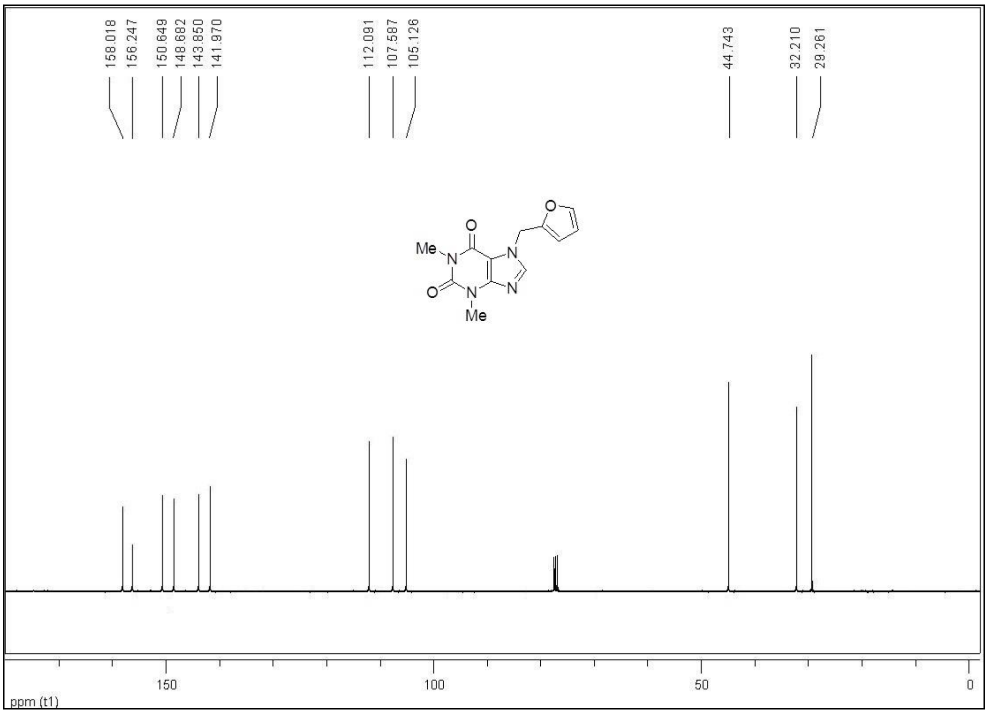
158.018
156.247
150.649
148.682
143.850
141.970
112.091
107.587
105.126
44.743
32.210
29.261
Me
N
O
N
N
N
O
Me
O
150
100
50
0
ppm (t1)

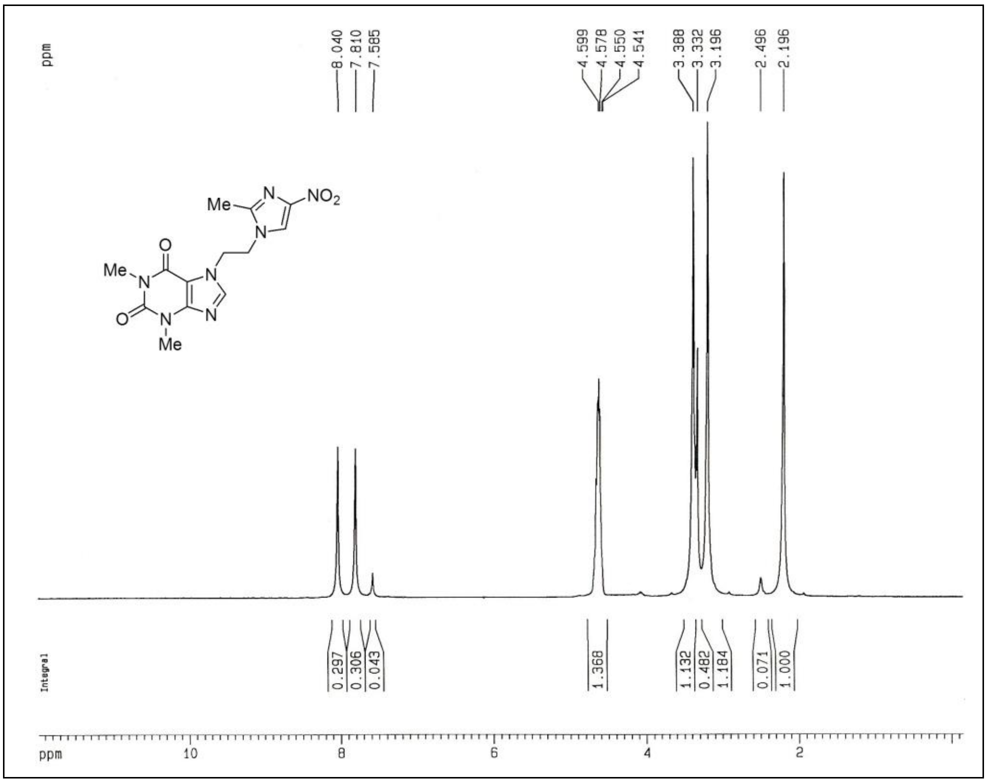
ppm
8.040
7.810
7.585
4.599
4.578
4.550
4.541
3.388
3.332
3.196
2.496
2.196
Me
NO2
O
Me
N
O
N
Me
Integral
0.297
0.306
0.043
1.368
1.132
0.482
1.184
0.071
1.000
ppm
10
8
6
4
2

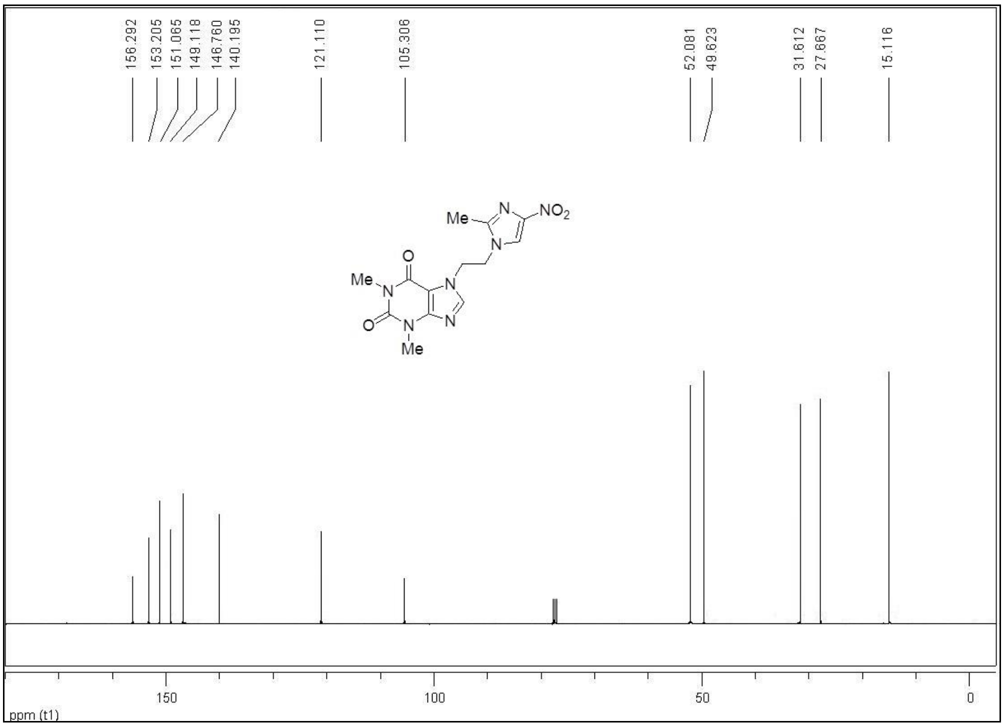
156.292
153.205
151.065
149.118
146.760
140.195
121.110
105.306
52.081
49.623
31.612
27.667
15.116
Me
NO2
O
Me
N
O
N
Me
150
100
50
0
ppm (t1)

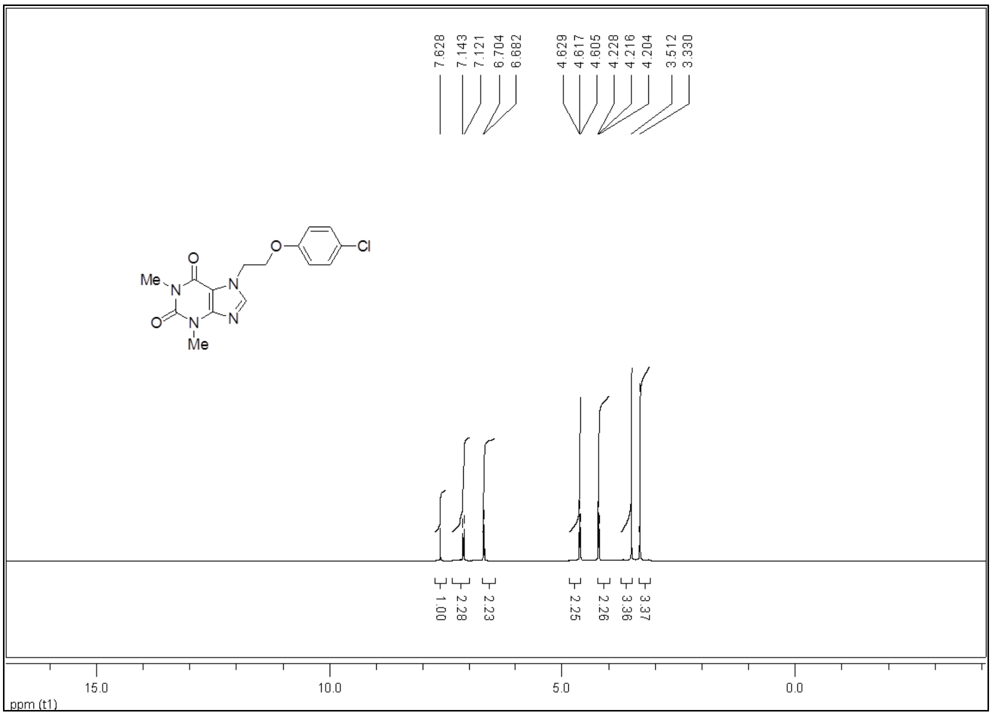
7.628
7.143
7.121
6.704
6.682
4.629
4.617
4.605
4.228
4.216
4.204
3.512
3.330
Me
N
O
O
N
N
N
Me
O
Cl
1.00
2.28
2.23
2.25
2.26
3.36
3.37
15.0
10.0
5.0
0.0
ppm (t1)

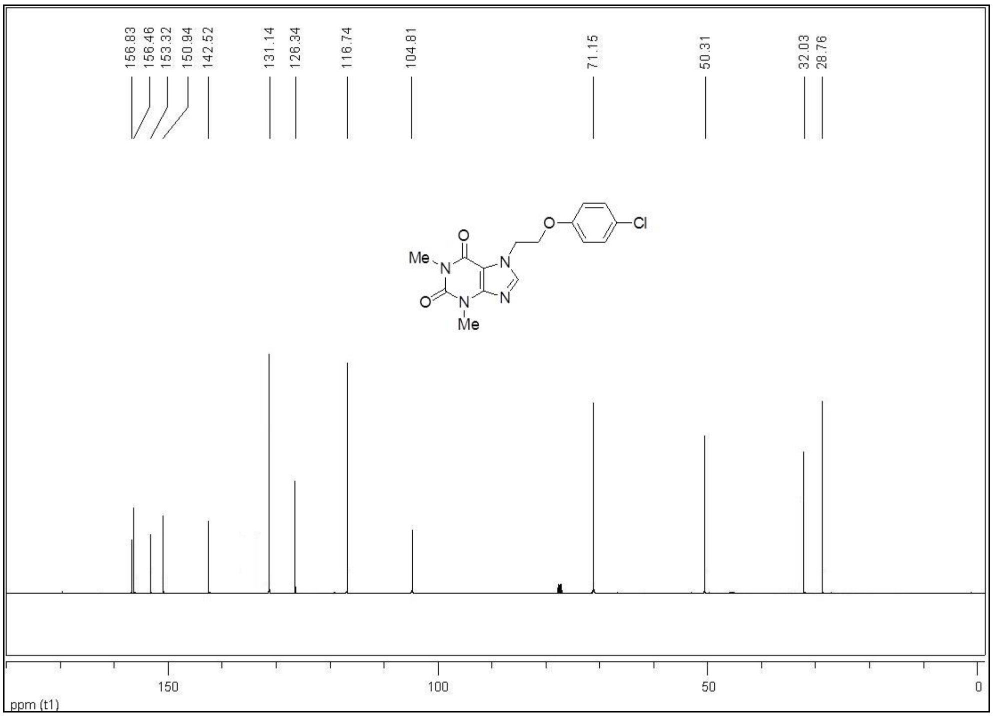
156.83
156.46
153.32
150.94
142.52
131.14
126.34
116.74
104.81
71.15
50.31
32.03
28.76
Me
N
O
O
N
N
N
Me
O
Cl
150
100
50
0
ppm (t1)

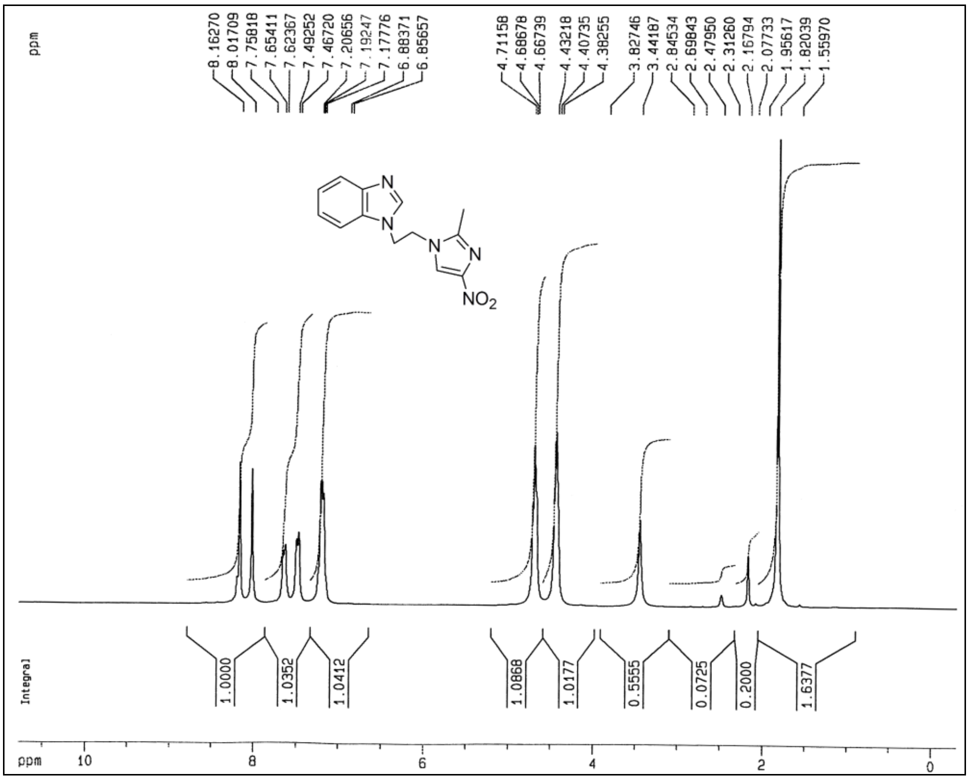
ppm
8.16270
8.01709
7.75818
7.65411
7.62367
7.49252
7.46720
7.20656
7.19247
7.17776
6.88371
6.85657
4.71158
4.68678
4.66739
4.43218
4.40735
4.38255
3.82746
3.44187
2.84534
2.69843
2.47950
2.31260
2.16794
2.07733
1.95617
1.82039
1.55970
N
N
N
N
$NO_2$
Integral
1.0000
1.0352
1.0412
1.0868
1.0177
0.5555
0.0725
0.2000
1.6377
ppm
10
8
6
4
2
0

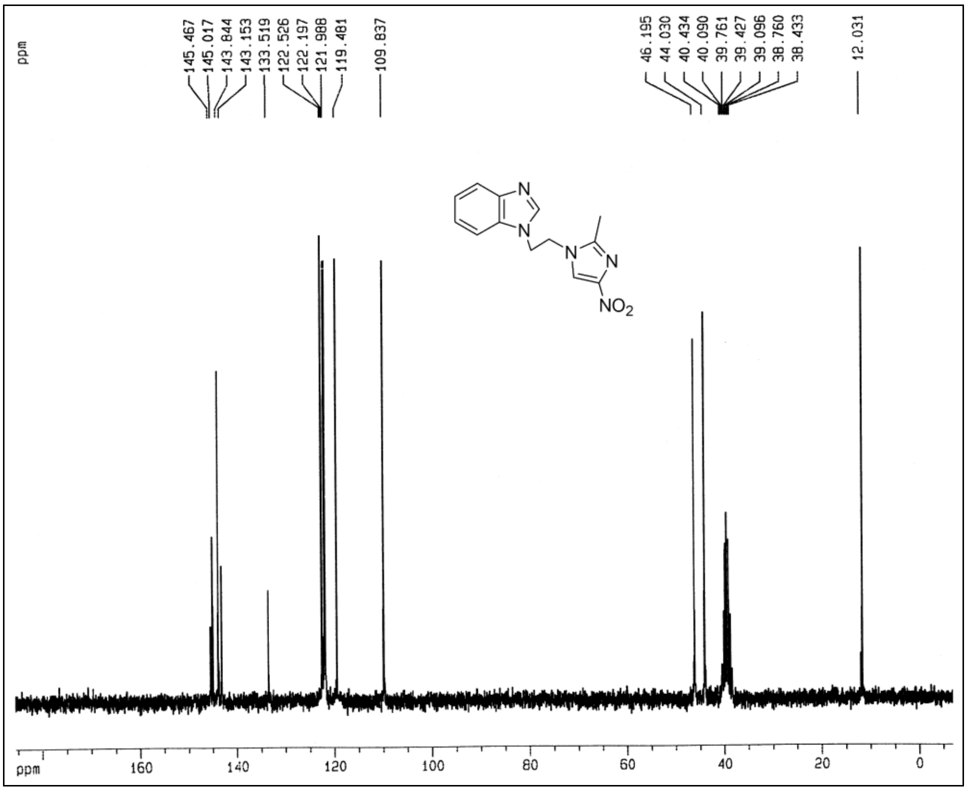
ppm
145.467
145.017
143.844
143.153
133.519
122.526
122.197
121.988
119.481
109.837
46.195
44.030
40.434
40.090
39.761
39.427
39.096
38.760
38.433
12.031
N
N
N
N
$NO_2$
ppm
160
140
120
100
80
60
40
20
0

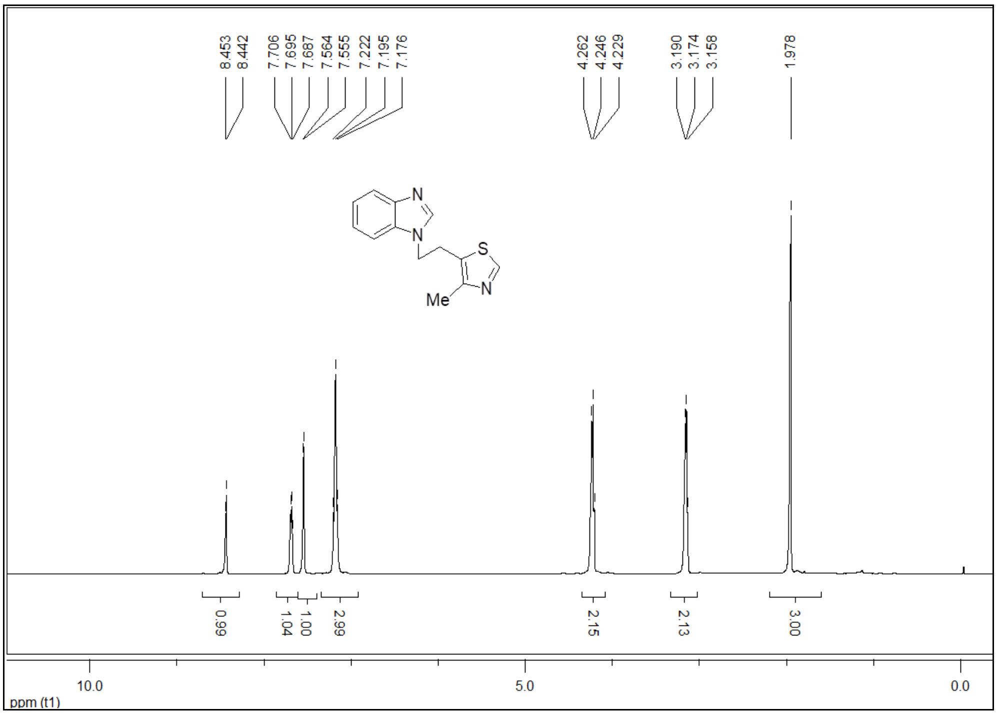
8.453
8.442
7.706
7.695
7.687
7.564
7.555
7.222
7.195
7.176
4.262
4.246
4.229
3.190
3.174
3.158
1.978
N
N
S
N
Me
0.99
1.04
1.00
2.99
2.15
2.13
3.00
10.0
5.0
0.0
ppm (t1)

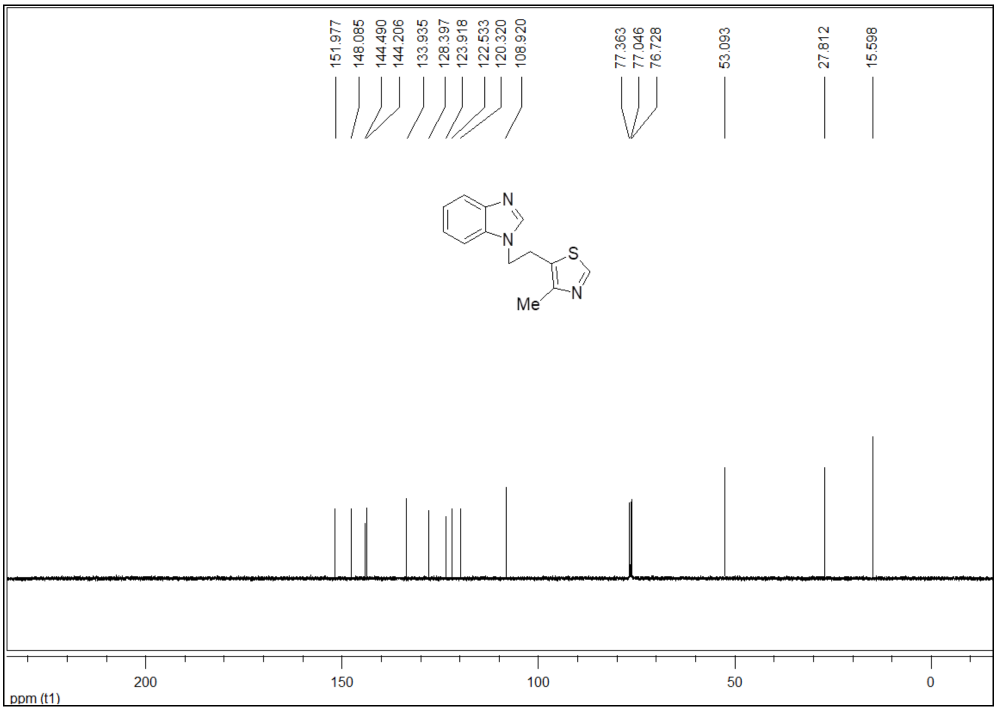
151.977
148.085
144.490
144.206
133.935
128.397
123.918
122.533
120.320
108.920
77.363
77.046
76.728
53.093
27.812
15.598
N
N
S
N
Me
200
150
100
50
0
ppm (t1)

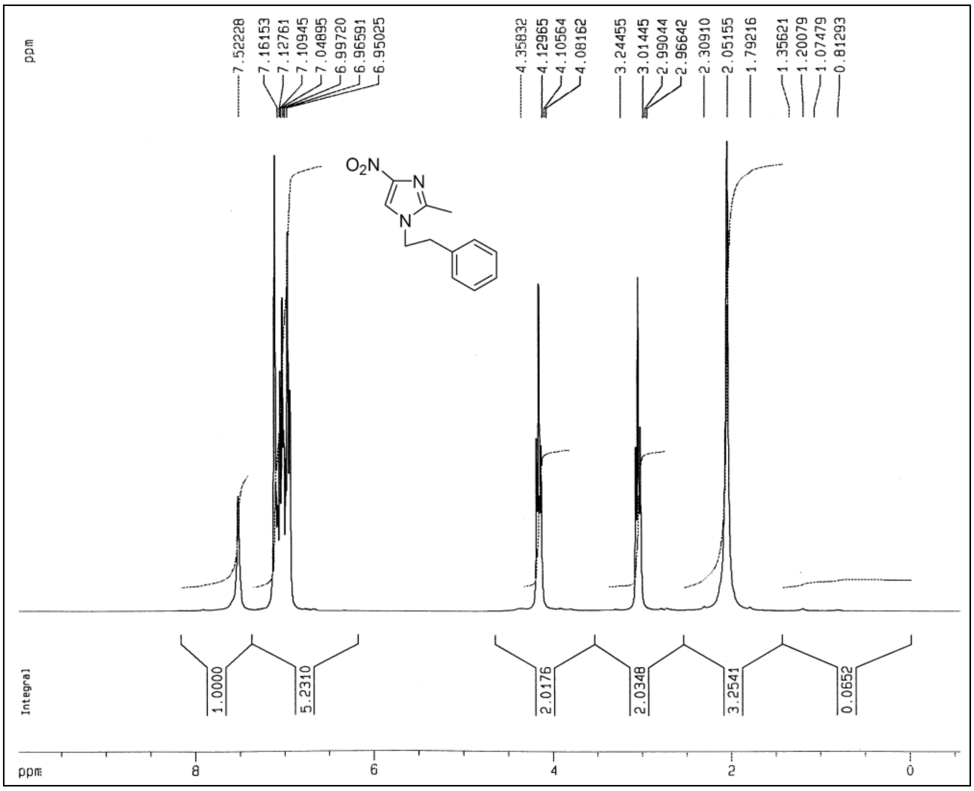
ppm
7.52228
7.16153
7.12761
7.10945
7.04895
6.99720
6.96591
6.95025
4.35832
4.12965
4.10564
4.08162
3.24455
3.01445
2.99044
2.96642
2.30910
2.05155
1.79216
1.35621
1.20079
1.07479
0.81293
$O_2N$
N
N
Integral
1.0000
5.2310
2.0176
2.0348
3.2541
0.0652
ppm
8
6
4
2
0

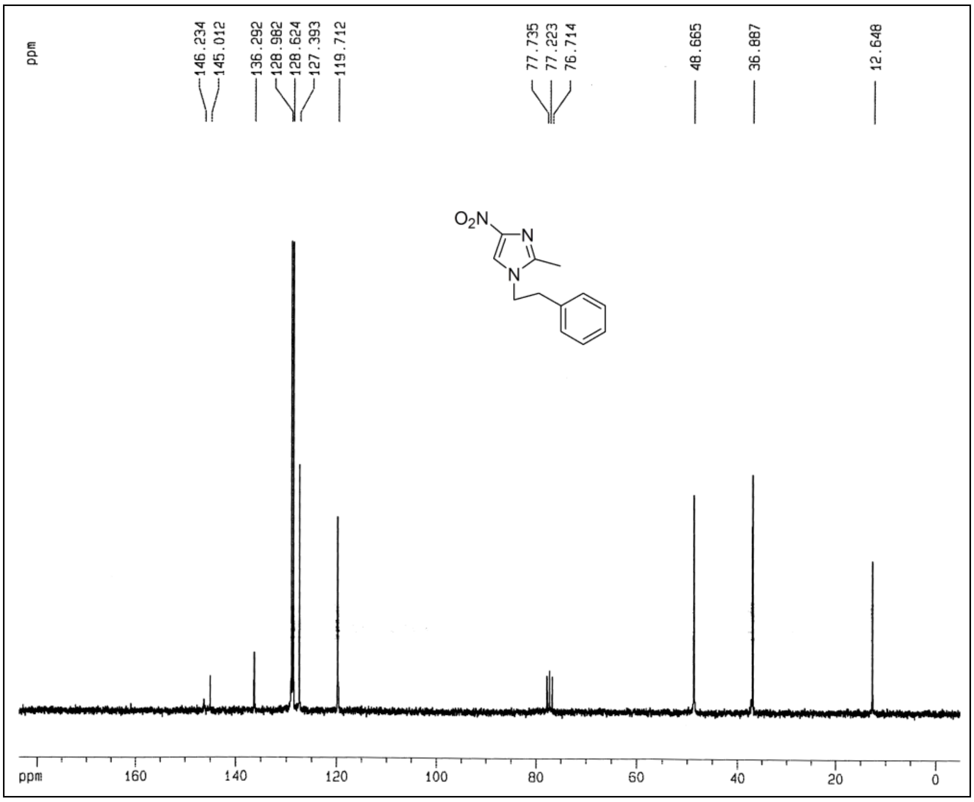
ppm
146.234
145.012
136.292
128.982
128.624
127.393
119.712
77.735
77.223
76.714
48.665
36.887
12.648
$O_2N$
N
N
ppm
160
140
120
100
80
60
40
20
0

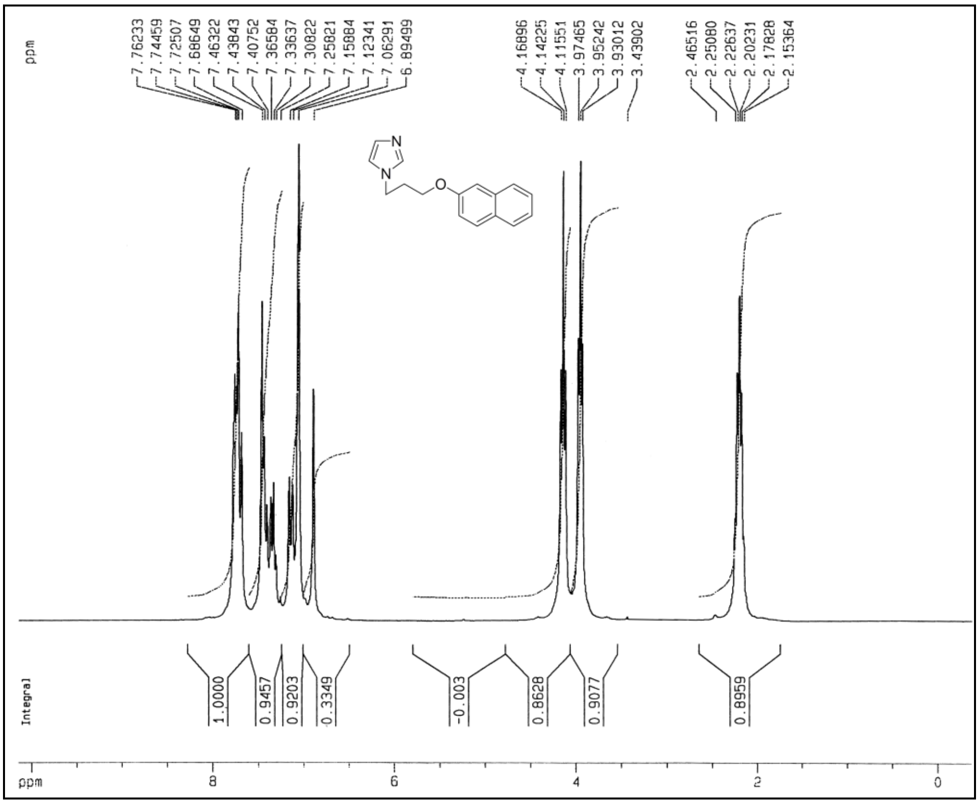
ppm
7.76233
7.74459
7.72507
7.68649
7.46322
7.43843
7.40752
7.36584
7.33637
7.30822
7.25821
7.15884
7.12341
7.06291
6.89499
4.16896
4.14225
4.11551
3.97465
3.95242
3.93012
3.43902
2.46516
2.25080
2.22637
2.20231
2.17828
2.15364
N
N
O
Integral
1.0000
0.9457
0.9203
0.3349
-0.003
0.8628
0.9077
0.8959
ppm
8
6
4
2
0

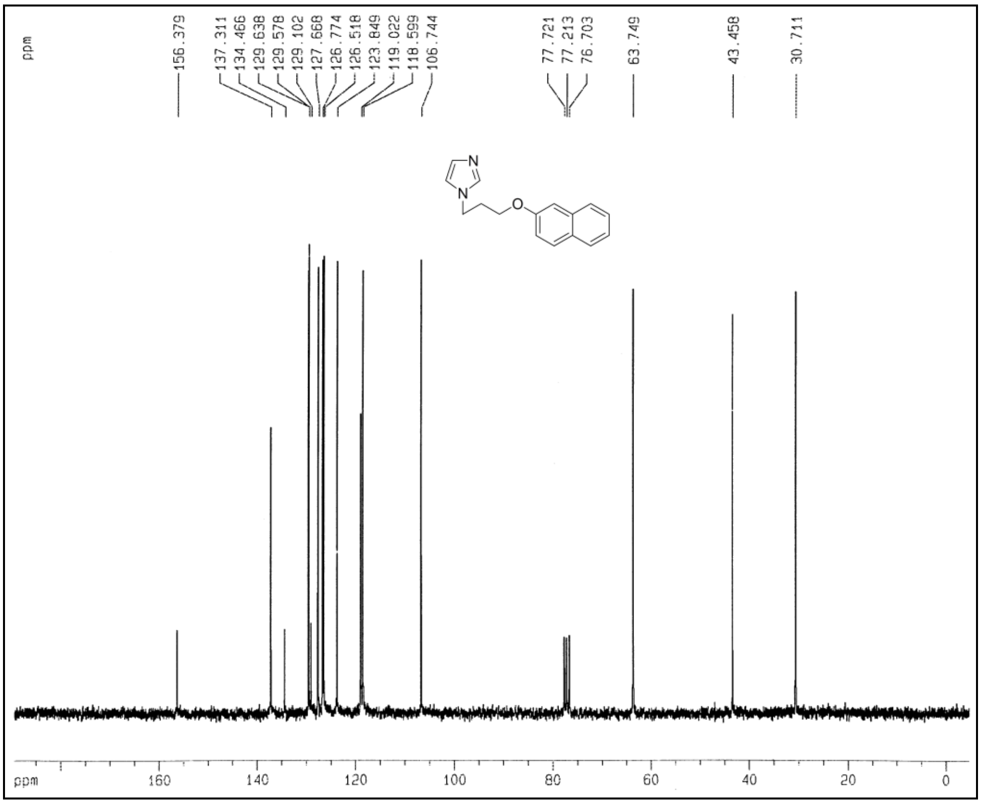
ppm
156.379
137.311
134.466
129.638
129.578
129.102
127.668
126.774
126.518
123.849
119.022
118.599
106.744
77.721
77.213
76.703
63.749
43.458
30.711
N
N
O
ppm
160
140
120
100
80
60
40
20
0

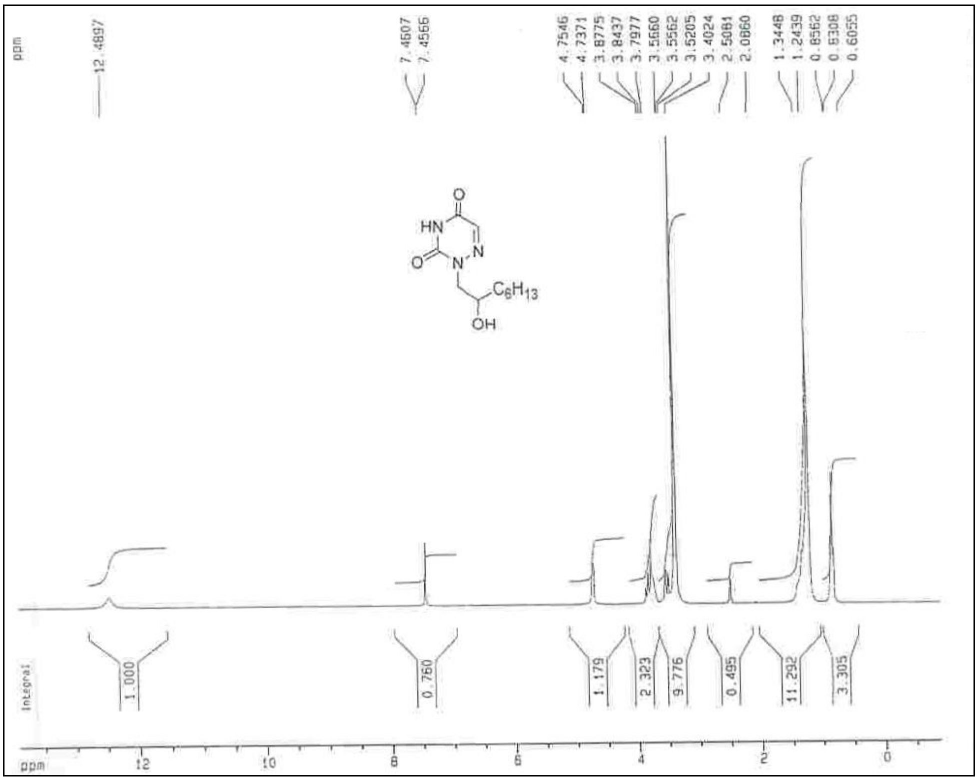

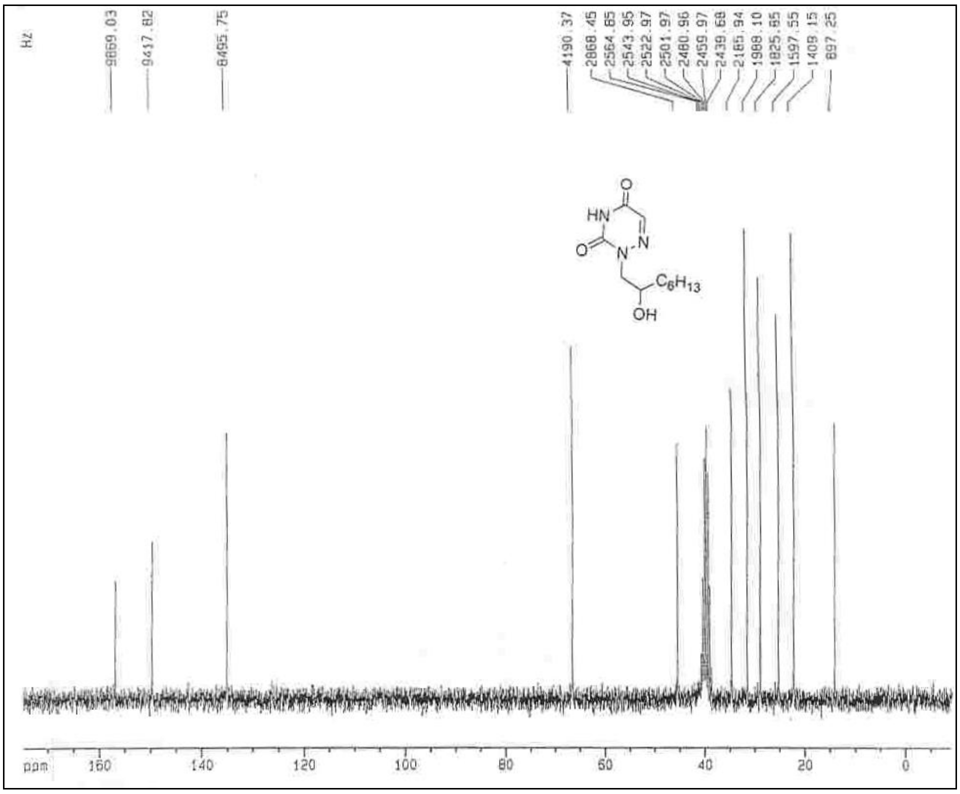

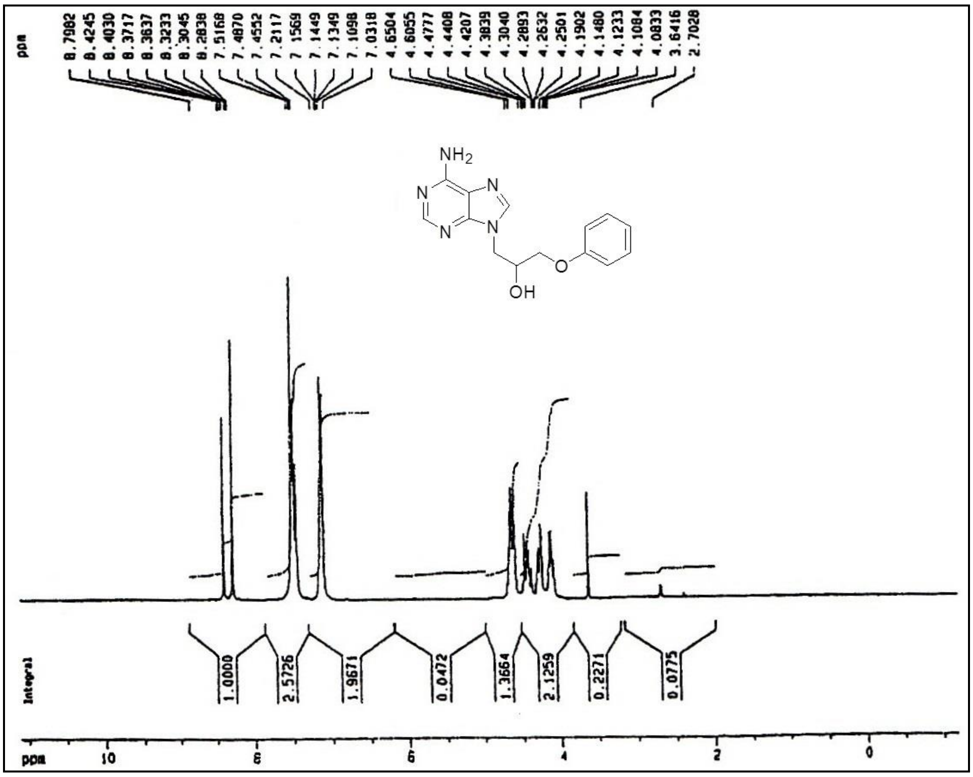

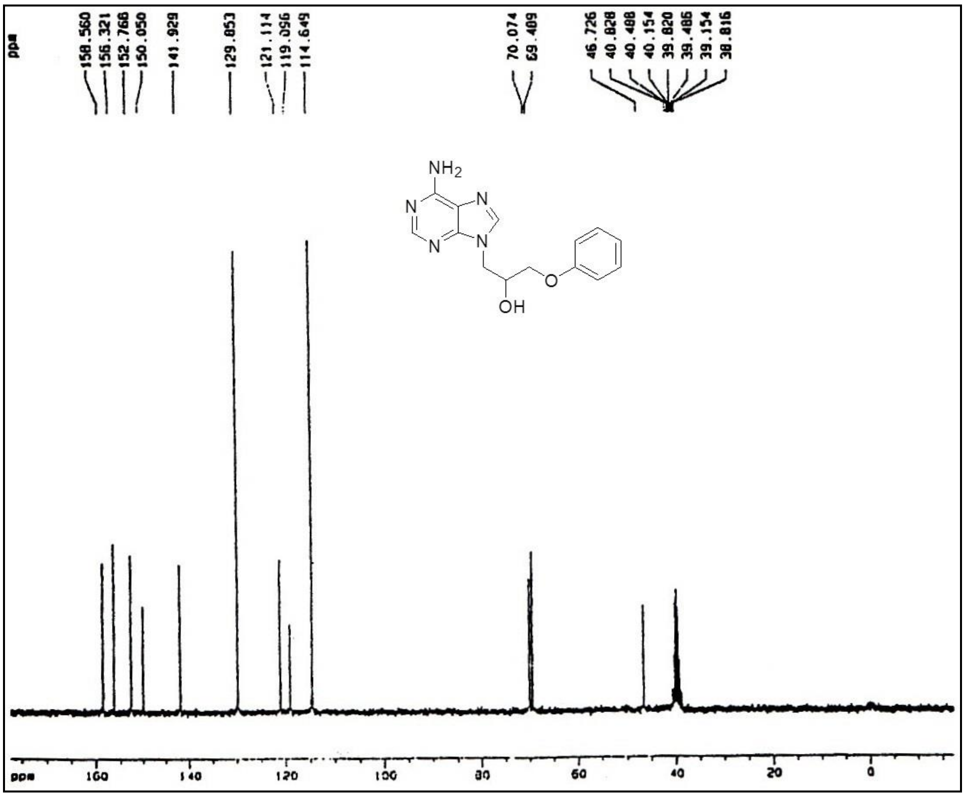

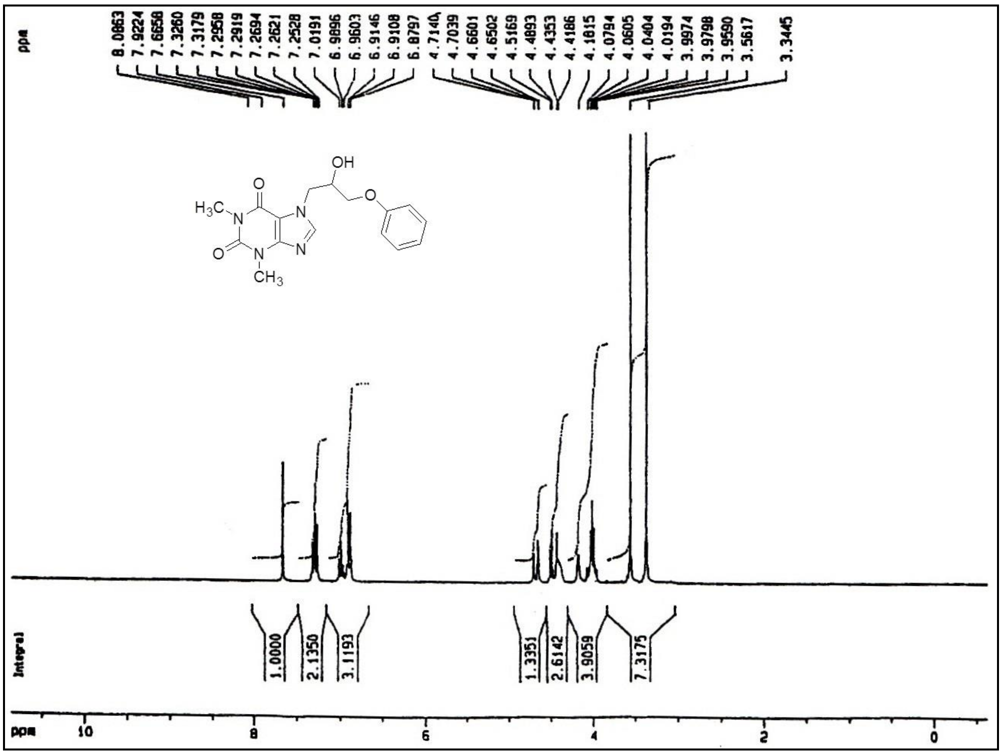

8.0863
7.9224
7.6658
7.3260
7.3179
7.2958
7.2919
7.2694
7.2621
7.2528
7.0191
6.9896
6.9603
6.9146
6.9108
6.8797
4.7140
4.7039
4.6601
4.6502
4.5169
4.4893
4.4353
4.4186
4.1815
4.0794
4.0605
4.0404
4.0194
3.9974
3.9798
3.9590
3.5617
3.3445
OH
O
$H_3C$
N
N
N
O
N
$CH_3$
O
Integral
1.0000
2.1350
3.1193
1.3351
2.6142
3.9059
7.3175
ppm
10
8
6
4
2
0


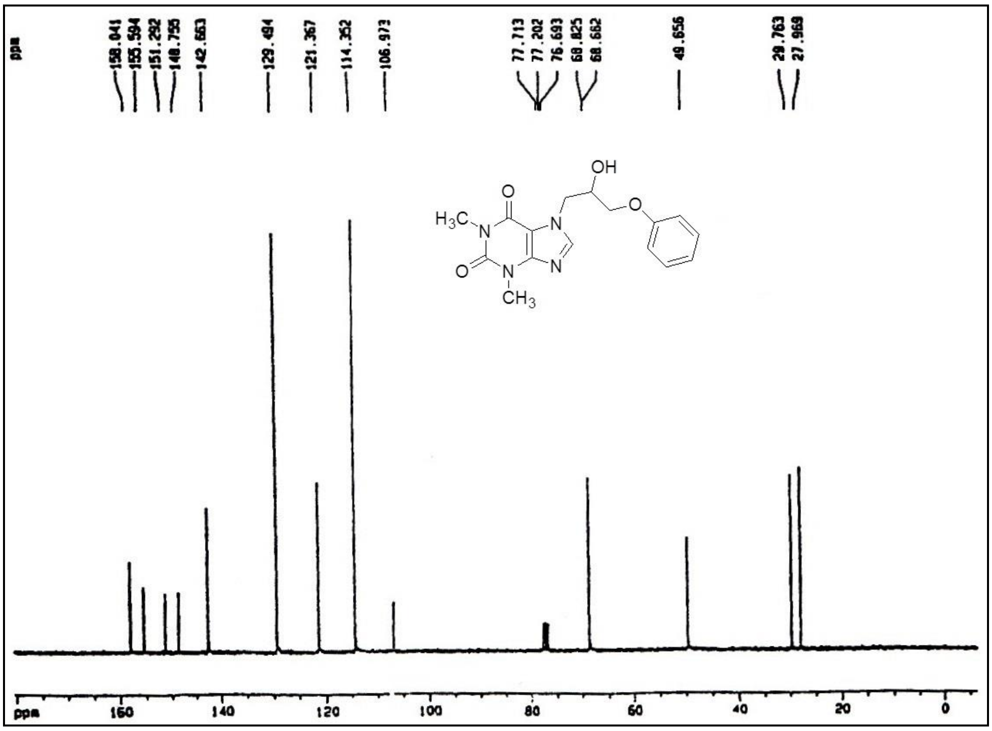

ppm
158.041
155.594
151.292
148.755
142.663
129.494
121.367
114.352
106.973
77.713
77.202
76.693
68.825
68.662
49.656
29.763
27.968
OH
O
$H_3C$
N
N
N
O
N
$CH_3$
O
160
140
120
100
80
60
40
20
0

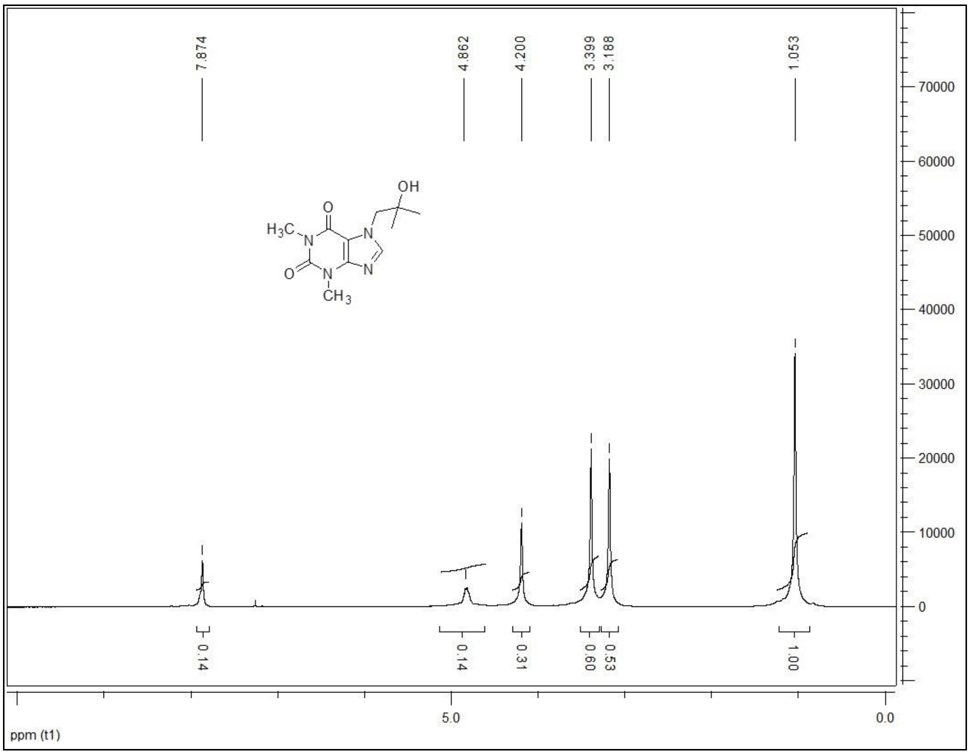

7.874
4.862
4.200
3.399
3.188
1.053
70000
60000
50000
40000
30000
20000
10000
0
0.14
0.14
0.31
0.60
0.53
1.00
5.0
0.0
ppm (t1)


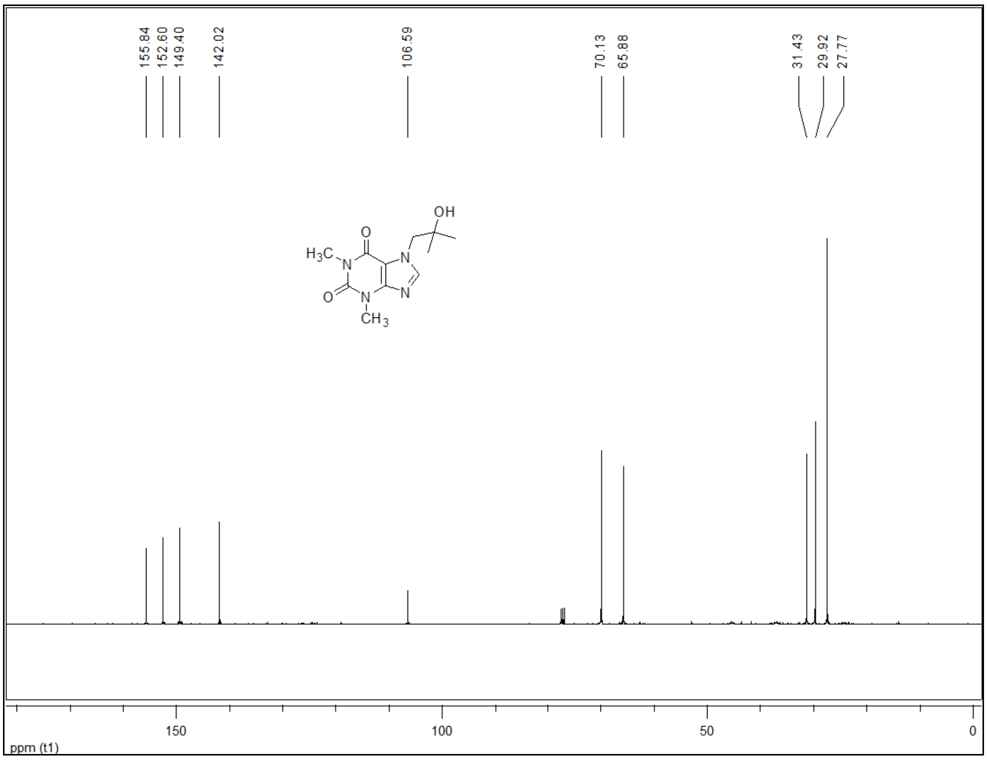

155.84
152.60
149.40
142.02
106.59
70.13
65.88
31.43
29.92
27.77
150
100
50
0
ppm (t1)

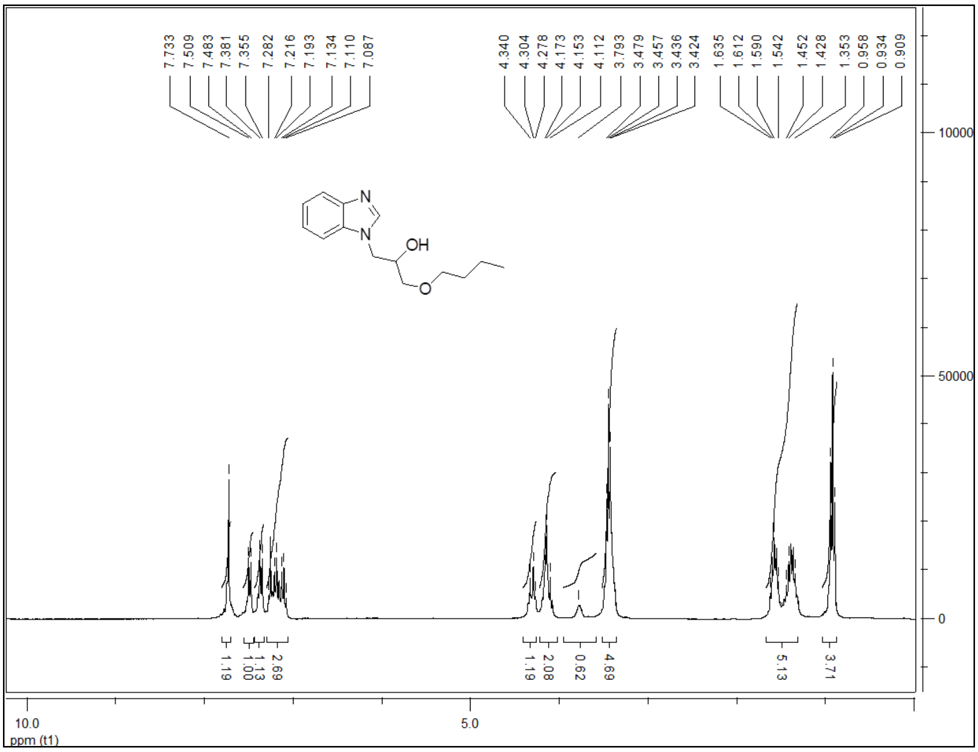

7.733
7.509
7.483
7.381
7.355
7.282
7.216
7.193
7.134
7.110
7.087
4.340
4.304
4.278
4.173
4.153
4.112
3.793
3.479
3.457
3.436
3.424
1.635
1.612
1.590
1.542
1.452
1.428
1.353
0.958
0.934
0.909
N
N
OH
O
1.19
1.00
1.13
2.69
1.19
2.08
0.62
4.69
5.13
3.71
10.0
5.0
ppm (t1)
100000
50000
0


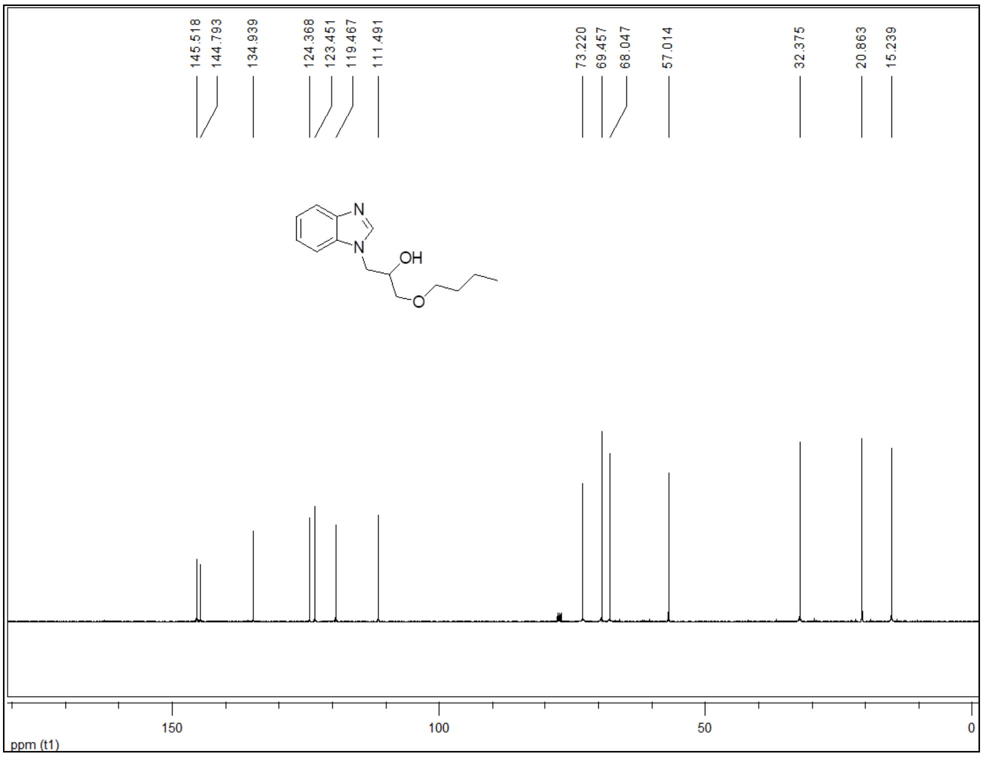

145.518
144.793
134.939
124.368
123.451
119.467
111.491
73.220
69.457
68.047
57.014
32.375
20.863
15.239
N
N
OH
O
150
100
50
0
ppm (t1)

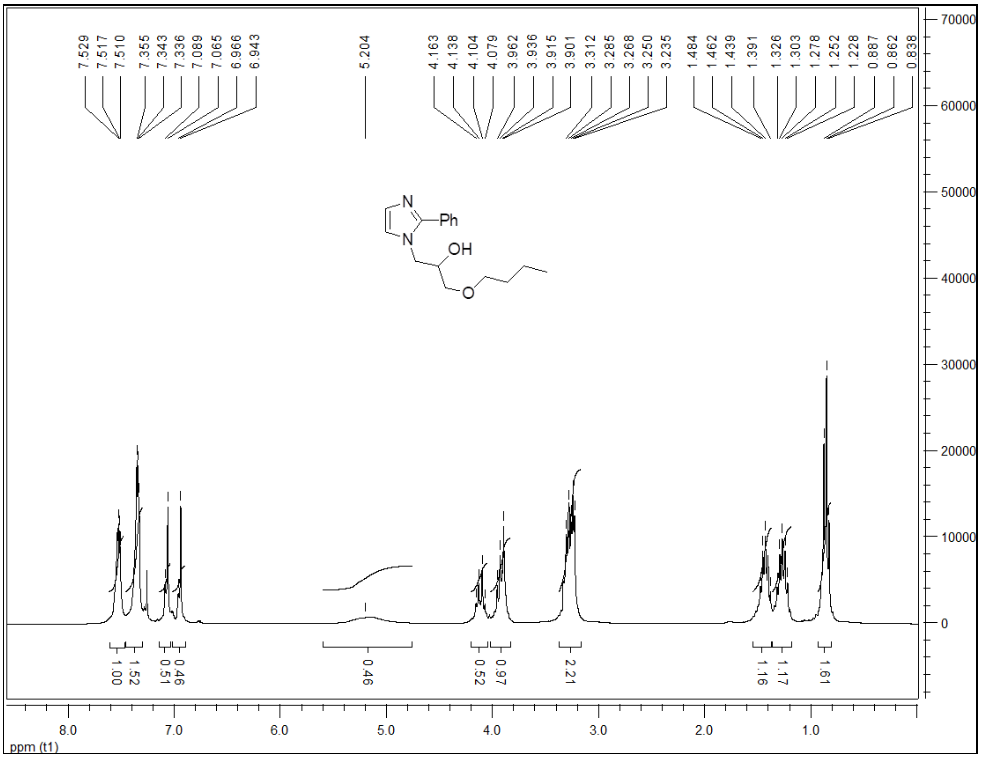
7.529
7.517
7.510
7.355
7.343
7.336
7.089
7.065
6.966
6.943
5.204
4.163
4.138
4.104
4.079
3.962
3.936
3.915
3.901
3.312
3.285
3.268
3.250
3.235
1.484
1.462
1.439
1.391
1.326
1.303
1.278
1.252
1.228
0.887
0.862
0.838
N
Ph
OH
O
1.00
1.52
0.51
0.46
0.46
0.52
0.97
2.21
1.16
1.17
1.61
70000
60000
50000
40000
30000
20000
10000
0
8.0
7.0
6.0
5.0
4.0
3.0
2.0
1.0
ppm (t1)

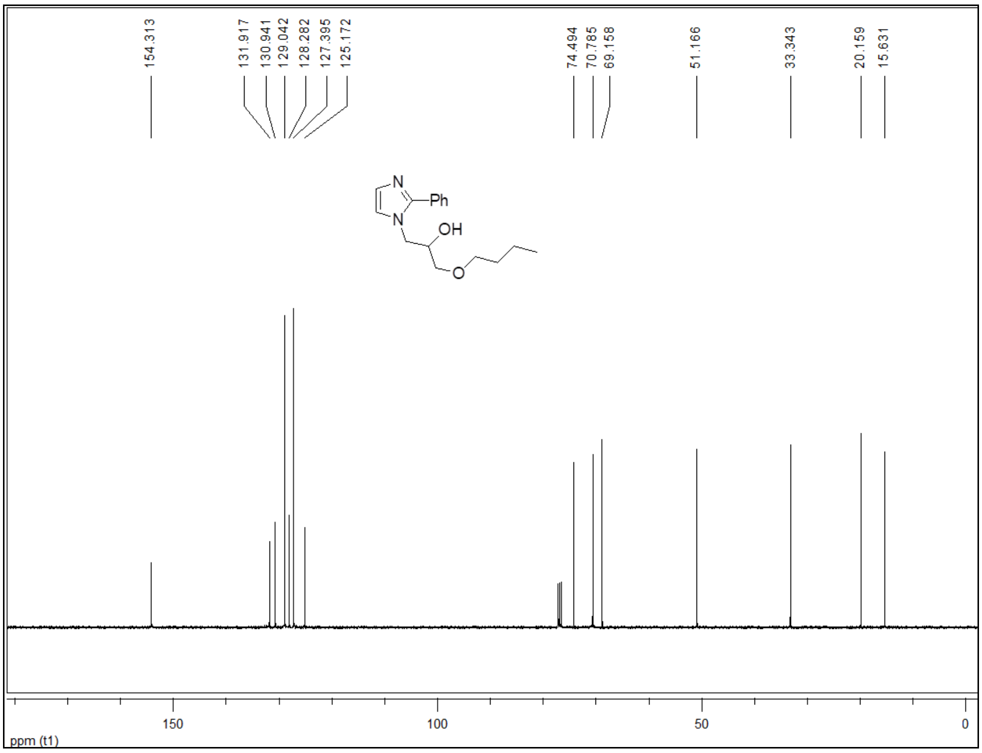
154.313
131.917
130.941
129.042
128.282
127.395
125.172
74.494
70.785
69.158
51.166
33.343
20.159
15.631
N
Ph
OH
O
150
100
50
0
ppm (t1)

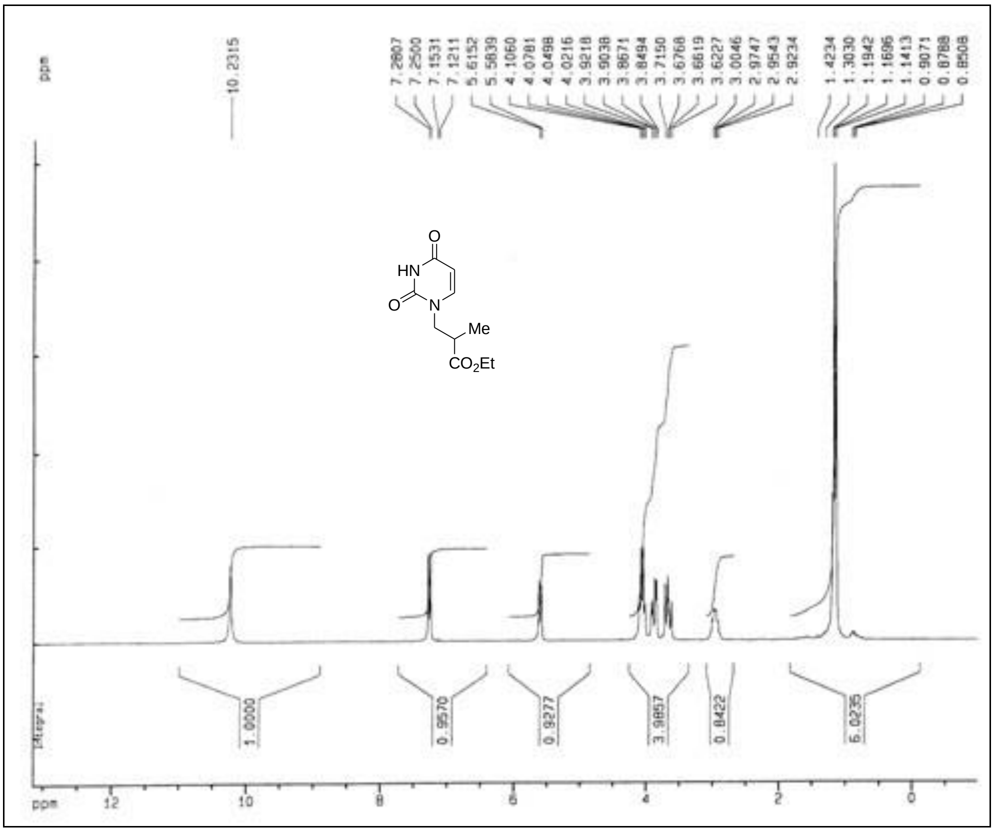

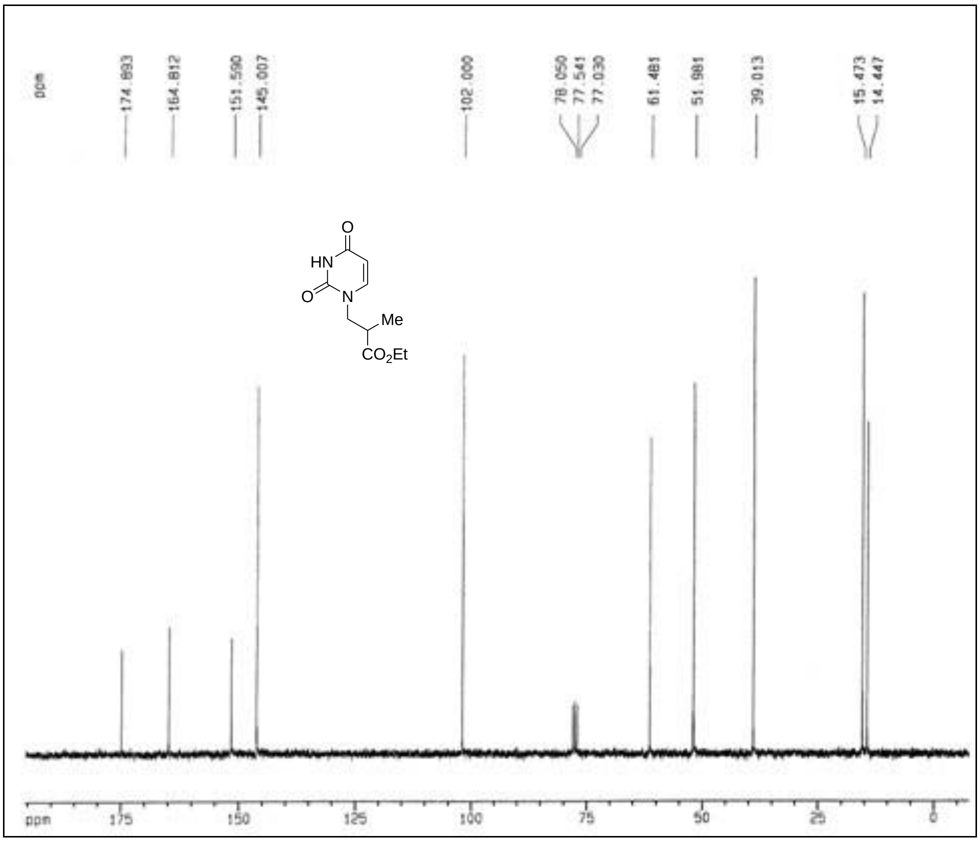

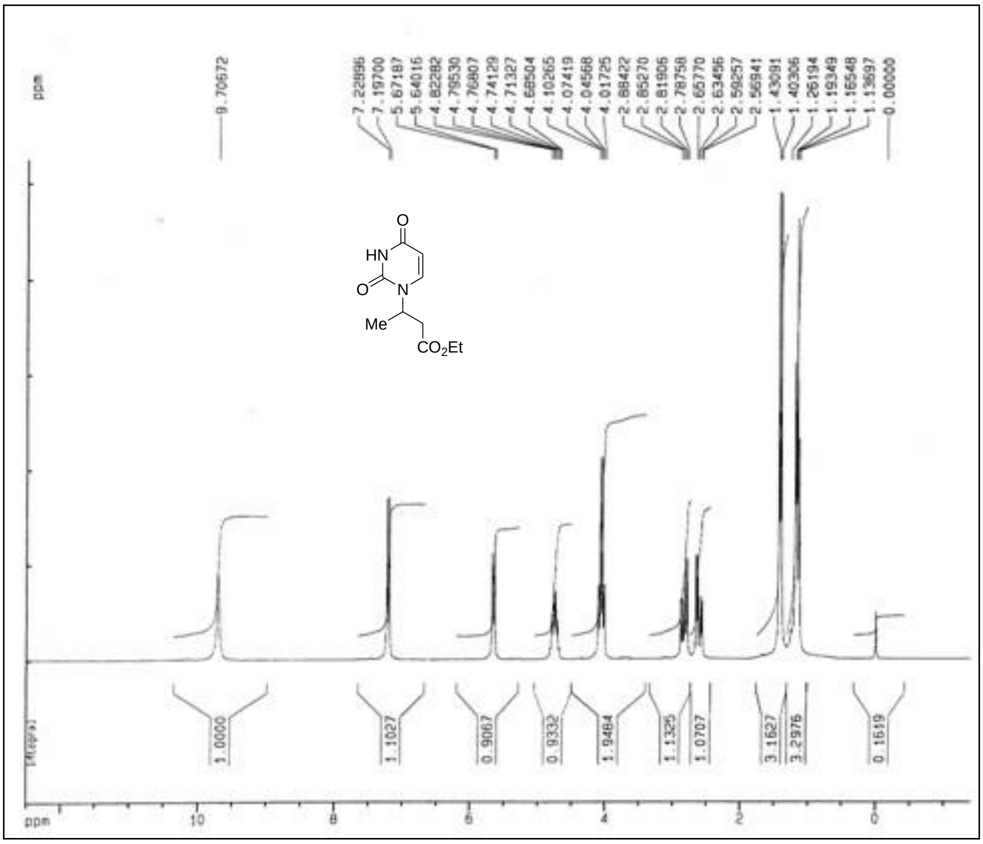
ppm
9.70672
7.22896
7.19700
5.67187
5.64016
4.82282
4.79530
4.76807
4.74129
4.71327
4.68504
4.10265
4.07419
4.04568
4.01725
2.88422
2.85270
2.81906
2.78758
2.65770
2.63456
2.59257
2.56941
1.43081
1.40306
1.26194
1.19349
1.16548
1.13697
0.00000
O
HN
O
N
Me
CO2Et
1.0000
1.1027
0.9067
0.9332
1.9484
1.1325
1.0707
3.1627
3.2976
0.1619
ppm

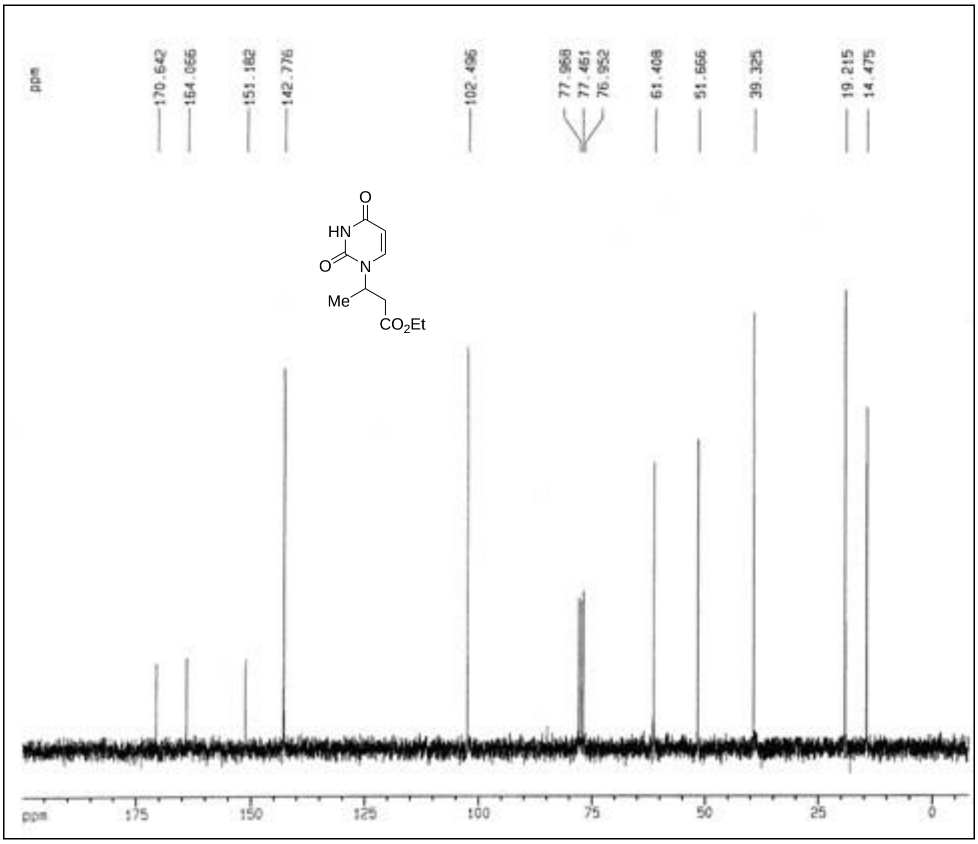
ppm
170.642
164.066
151.182
142.776
102.496
77.968
77.461
76.952
61.408
51.666
39.325
19.215
14.475
O
HN
O
N
Me
CO2Et
ppm

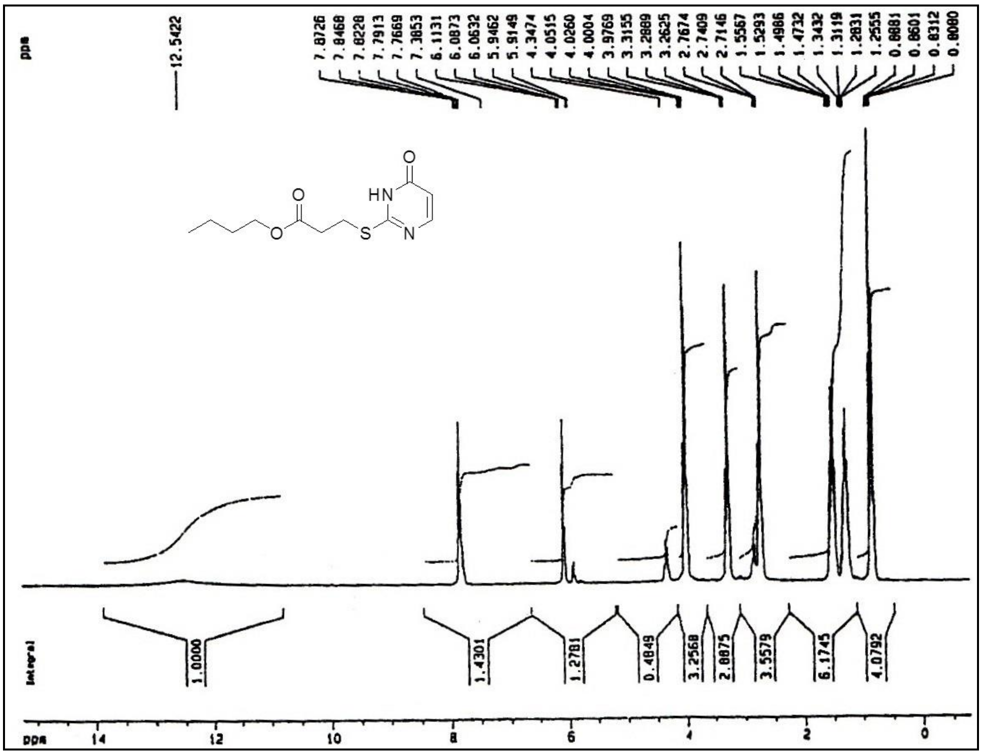
ppm
12.5422
7.8726 7.8468 7.8228 7.7913 7.7669 7.3853 6.1131 6.0873 6.0632 5.9462 5.9149 4.3474 4.0515 4.0260 4.0004 3.9769 3.3155 3.2889 3.2625 2.7674 2.7409 2.7146 1.5567 1.5293 1.4986 1.4732 1.3432 1.3119 1.2831 1.2555 0.8881 0.8601 0.8312 0.8080
O
HN
O
O
S
N
Integral
1.0000 1.4301 1.2781 0.4849 3.2568 2.8875 3.5579 6.1745 4.0792
ppm 14 12 10 8 6 4 2 0

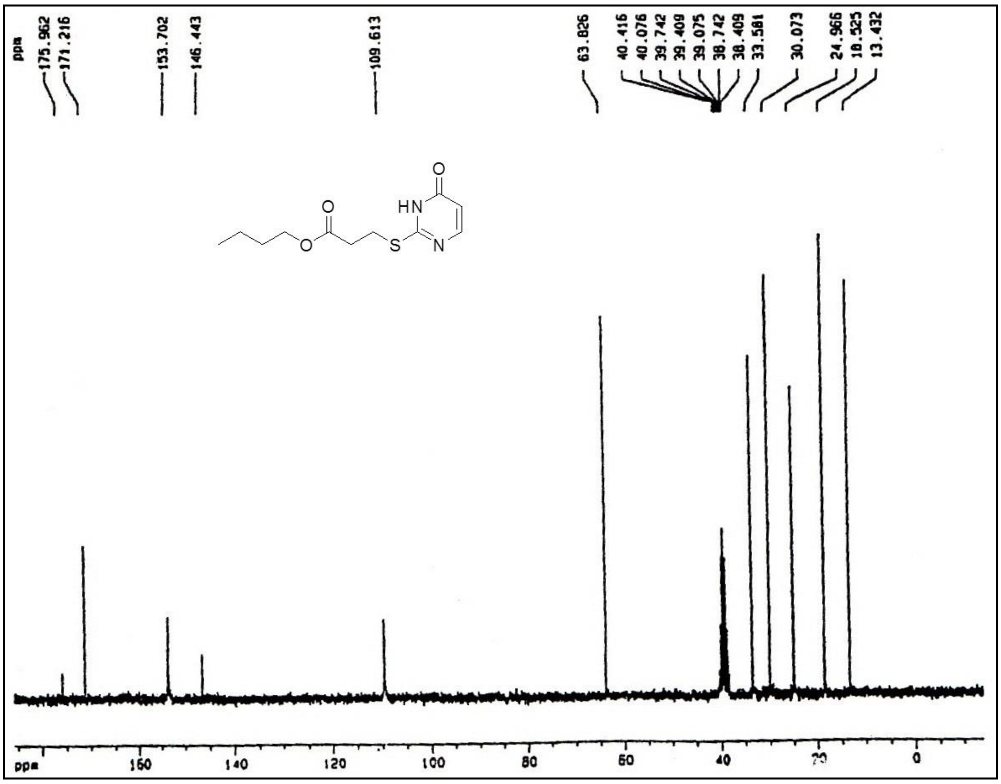
ppm
175.962 171.216 153.702 146.443 109.613 63.826 40.416 40.076 39.742 39.409 39.075 38.742 38.409 33.581 30.073 24.966 18.525 13.432
O
HN
O
O
S
N
ppm 160 140 120 100 80 60 40 20 0

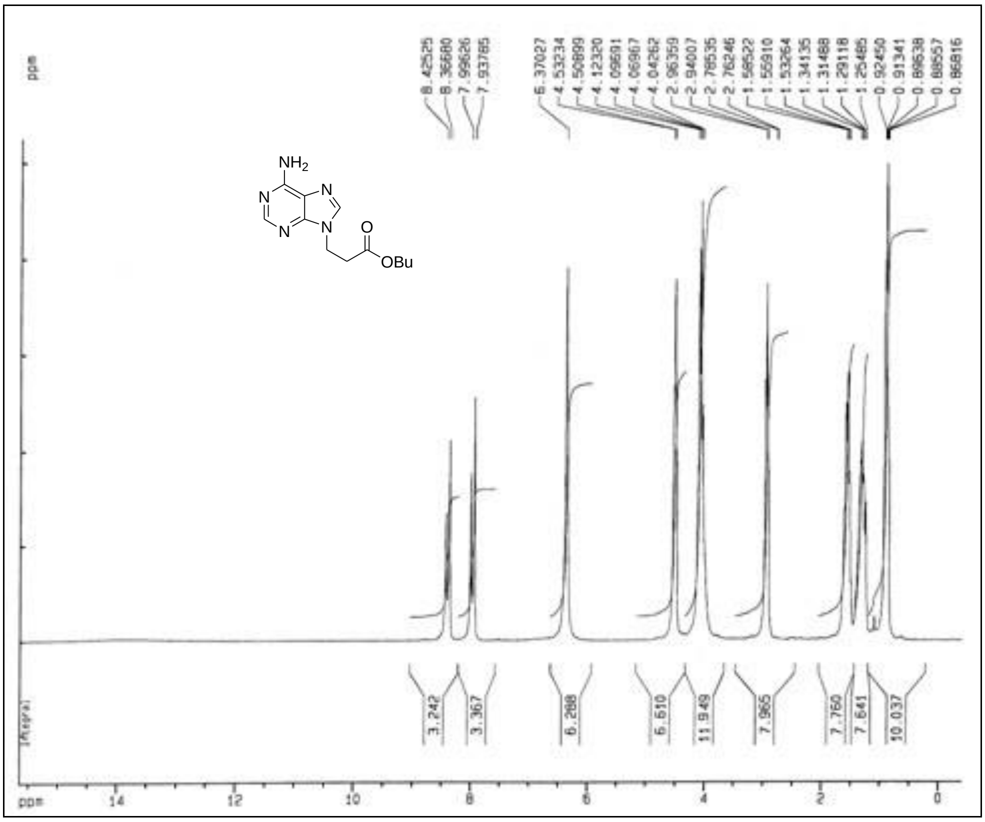
ppm
8.42525
8.36680
7.99626
7.93785
6.37027
4.53234
4.50899
4.12320
4.09691
4.06967
4.04262
2.96359
2.94007
2.78535
2.76246
1.58522
1.55910
1.53264
1.34135
1.31488
1.29118
1.25485
0.92450
0.91341
0.89638
0.88557
0.86816
NH2
N
N
N
N
O
OBu
3.242
3.367
6.288
6.610
11.949
7.965
7.760
7.641
10.037
ppm
14
12
10
8
6
4
2
0

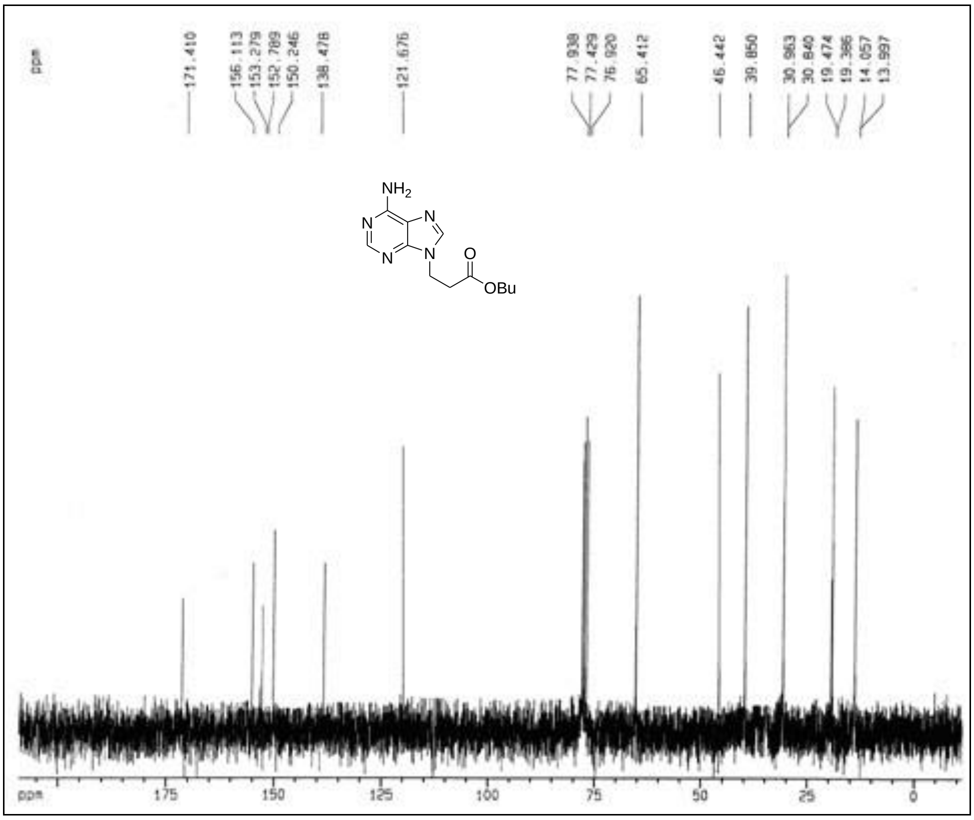
ppm
171.410
156.113
153.279
152.789
150.246
138.478
121.676
77.938
77.429
76.920
65.412
46.442
39.850
30.963
30.840
19.474
19.386
14.057
13.997
NH2
N
N
N
N
O
OBu
ppm
175
150
125
100
75
50
25
0

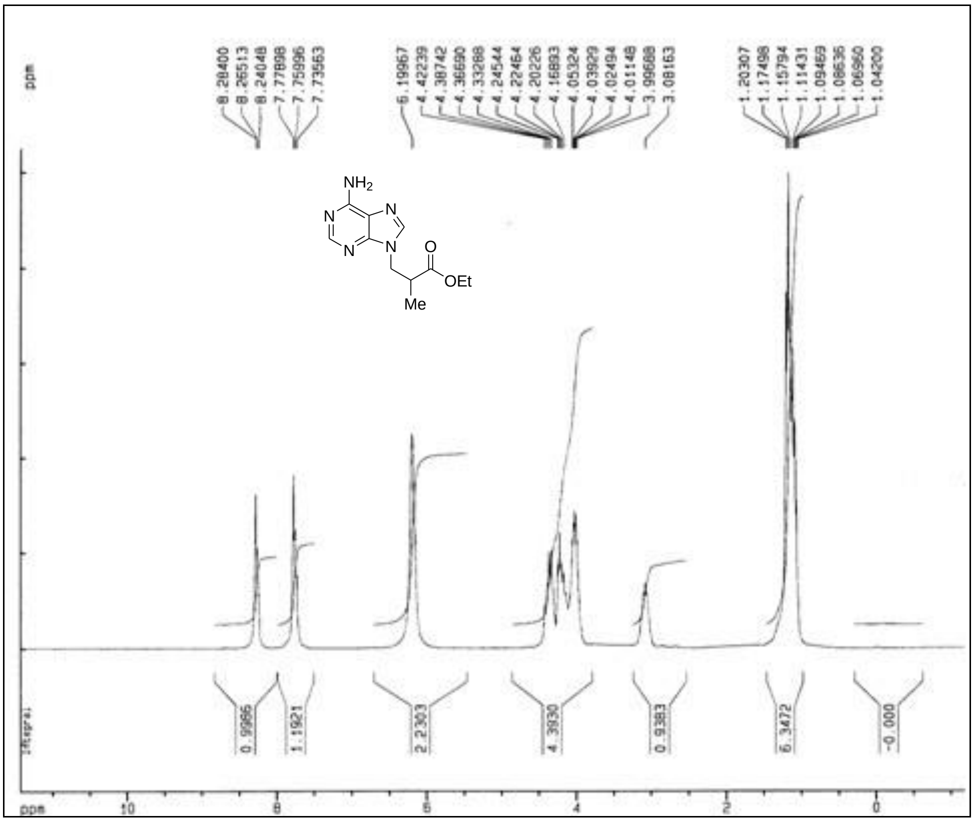

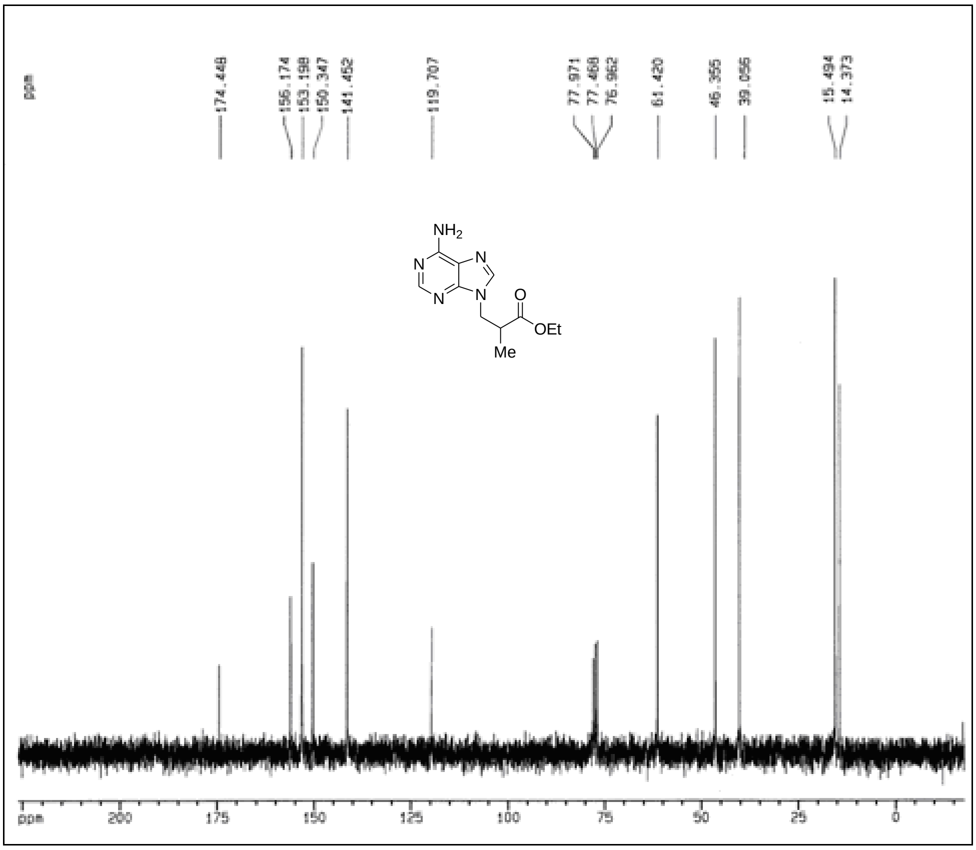

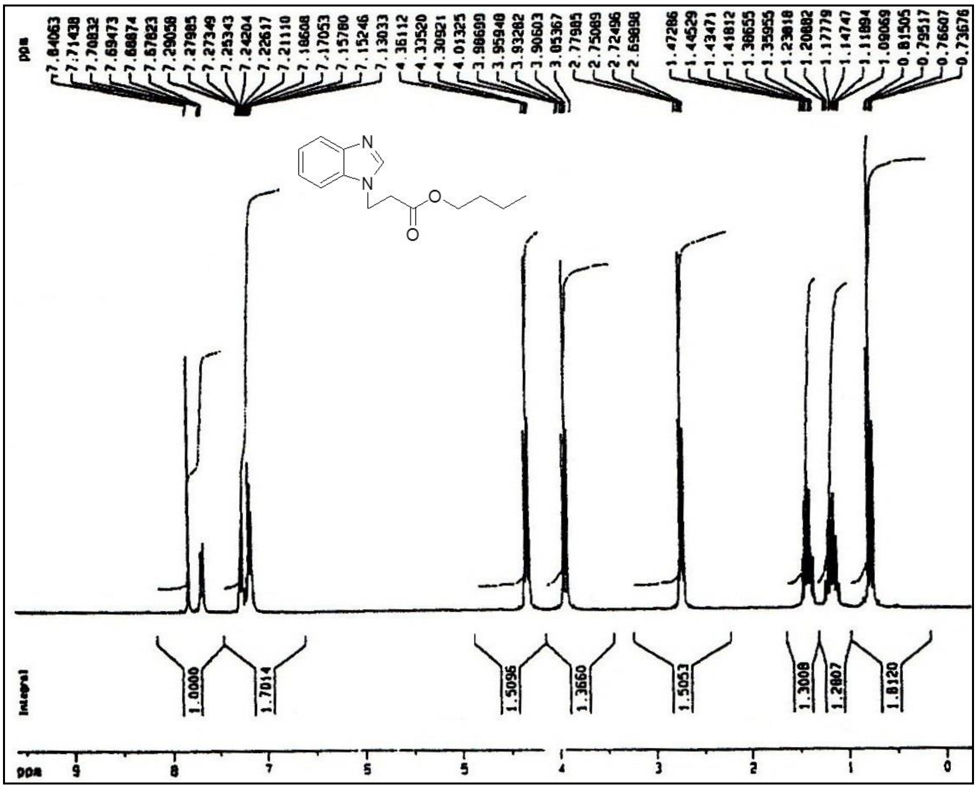

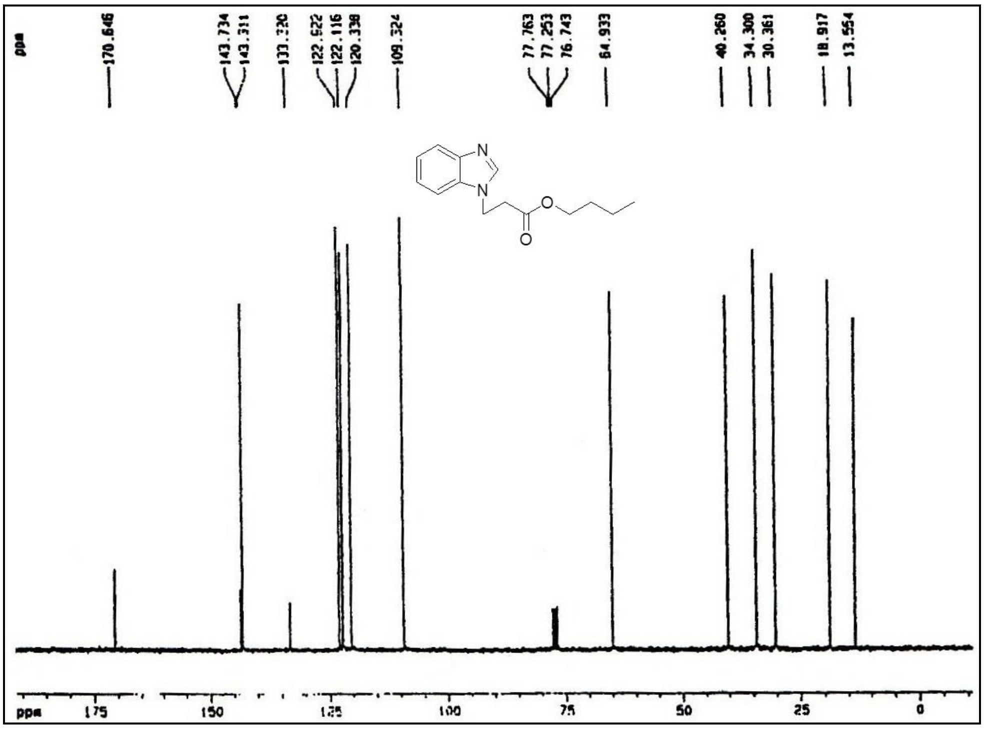

Supplement: RA-016-D5RA10042A-s001 [file RA-016-D5RA10042A-s001.pdf]
